# Functional analysis of the ScAG and ScAGL11 MADS-box transcription factors for anthocyanin biosynthesis and bicolour pattern formation in *Senecio cruentus* ray florets

**DOI:** 10.1093/hr/uhac071

**Published:** 2022-03-23

**Authors:** Fangting Qi, Yuting Liu, Yiliu Luo, Yumeng Cui, Chenfei Lu, Hao Li, He Huang, Silan Dai

**Affiliations:** Beijing Key Laboratory of Ornamental Plants Germplasm Innovation & Molecular Breeding, National Engineering Research Center for Floriculture, Beijing Laboratory of Urban and Rural Ecological Environment, Key Laboratory of Genetics and Breeding in Forest Trees and Ornamental Plants of Education Ministry, School of Landscape Architecture, Beijing Forestry University, Beijing, 100083, China; Beijing Key Laboratory of Ornamental Plants Germplasm Innovation & Molecular Breeding, National Engineering Research Center for Floriculture, Beijing Laboratory of Urban and Rural Ecological Environment, Key Laboratory of Genetics and Breeding in Forest Trees and Ornamental Plants of Education Ministry, School of Landscape Architecture, Beijing Forestry University, Beijing, 100083, China; Beijing Key Laboratory of Ornamental Plants Germplasm Innovation & Molecular Breeding, National Engineering Research Center for Floriculture, Beijing Laboratory of Urban and Rural Ecological Environment, Key Laboratory of Genetics and Breeding in Forest Trees and Ornamental Plants of Education Ministry, School of Landscape Architecture, Beijing Forestry University, Beijing, 100083, China; Beijing Key Laboratory of Ornamental Plants Germplasm Innovation & Molecular Breeding, National Engineering Research Center for Floriculture, Beijing Laboratory of Urban and Rural Ecological Environment, Key Laboratory of Genetics and Breeding in Forest Trees and Ornamental Plants of Education Ministry, School of Landscape Architecture, Beijing Forestry University, Beijing, 100083, China; Beijing Key Laboratory of Ornamental Plants Germplasm Innovation & Molecular Breeding, National Engineering Research Center for Floriculture, Beijing Laboratory of Urban and Rural Ecological Environment, Key Laboratory of Genetics and Breeding in Forest Trees and Ornamental Plants of Education Ministry, School of Landscape Architecture, Beijing Forestry University, Beijing, 100083, China; Beijing Key Laboratory of Ornamental Plants Germplasm Innovation & Molecular Breeding, National Engineering Research Center for Floriculture, Beijing Laboratory of Urban and Rural Ecological Environment, Key Laboratory of Genetics and Breeding in Forest Trees and Ornamental Plants of Education Ministry, School of Landscape Architecture, Beijing Forestry University, Beijing, 100083, China; Beijing Key Laboratory of Ornamental Plants Germplasm Innovation & Molecular Breeding, National Engineering Research Center for Floriculture, Beijing Laboratory of Urban and Rural Ecological Environment, Key Laboratory of Genetics and Breeding in Forest Trees and Ornamental Plants of Education Ministry, School of Landscape Architecture, Beijing Forestry University, Beijing, 100083, China; Beijing Key Laboratory of Ornamental Plants Germplasm Innovation & Molecular Breeding, National Engineering Research Center for Floriculture, Beijing Laboratory of Urban and Rural Ecological Environment, Key Laboratory of Genetics and Breeding in Forest Trees and Ornamental Plants of Education Ministry, School of Landscape Architecture, Beijing Forestry University, Beijing, 100083, China

## Abstract

Cineraria (*Senecio cruentus*) is an ornamental plant with pure colour and bicolour cultivars, widely used for landscaping. Anthocyanin biosynthesis influences coloration patterns in cineraria. However, how anthocyanins accumulate and distribute in cineraria is poorly understood. This study investigated the molecular mechanisms underlying anthocyanin biosynthesis and bicolour formation in cineraria using pure colour and bicolour cultivars. Transcriptome and gene expression analysis showed that five genes, *ScCHS2*, *ScF3H1*, *ScDFR3*, *ScANS*, and *ScbHLH17*, were inhibited in the white cultivar and colourless regions of bicolour cultivars. In contrast, two MADS-box genes, *ScAG* and *ScAGL11*, showed significantly higher expression in the colourless regions of bicolour cultivars. ScAG and ScAGL11 were localized in the nucleus and co-expressed with the bicolour trait. Further functional analysis verified that *ScAG* inhibits anthocyanin accumulation in tobacco (*Nicotiana tabacum*). However, virus-induced gene silencing (VIGS) experiments showed that silencing of *ScAG* and *ScAGL11* increases anthocyanin content in cineraria leaves. Similar results were observed when *ScAG* and *ScAGL11* were silenced in the cineraria capitulum, accompanied by the smaller size of the colourless region, specifically in the *ScAG*/*ScAGL11*-silenced plants. The expression of *ScCHS2*, *ScDFR3*, and *ScF3H1* increased in silenced cineraria leaves and capitulum. Furthermore, yeast two-hybrid and bimolecular fluorescence complementation experiments demonstrated that ScAG interacts with ScAGL11. Moreover, *ScAG* directly inhibited the transcription of *ScF3H1* while *ScAGL11* inhibited *ScDFR3* expression by binding to their promoters separately. The findings reported herein indicate that *ScAG* and *ScAGL11* negatively regulate anthocyanin biosynthesis in cineraria ray florets, and their differential expression in ray florets influences the bicolour pattern appearance.

## Introduction

**Figure 1:**
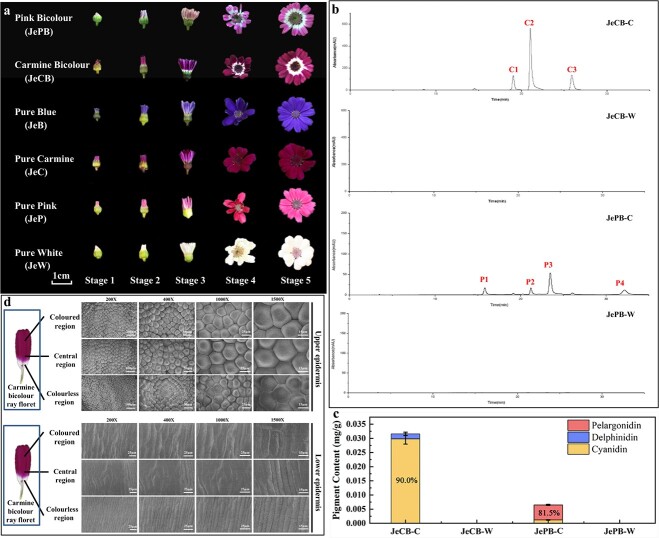
Characteristics of flower developmental stages, pigment composition, and epidermal structure in bicolour and pure colour cultivars of cineraria ‘Jester’. **a** Characteristics of bicolour (JePB and JeCB) and pure colour flowers (JeB, JeC, JeP, and JeW) at different developmental stages. Stage 1, flower bud stage; Stage 2, ray floret stretching stage; Stage 3, ray floret stretching complete stage; Stage 4, initiating blooming stage; and Stage 5, blooming stage. **b** HPLC chromatograms of anthocyanin extracts from coloured and colourless regions of JeCB and JePB at Stage 5. Supplementary Data Table S1 shows peak identification and characterization. The chromatogram was processed at 530 nm. **c** Pigment content analysis of two bicolour cultivars (JeCB and JePB). All data are presented as the mean ± standard deviation of three biological replicates. **d** SEM analysis of epidermal cell morphology in JeCB ray florets at Stage 5. Central region, region between the coloured and colourless regions; JeCB-C, coloured region of JeCB; JeCB-W, colourless region of JeCB; JePB-C, coloured region of JePB; JePB-W, colourless region of JePB.

Cineraria (*Senecio cruentus*) is an important ornamental plant with variable flower colours applied in landscaping. Our previous study identified pelargonidin, cyanidin, and delphinidin as the main pigments in pink, carmine, and blue cineraria cultivars, respectively [1]. These findings indicated cineraria as an ideal material for studying the regulation of anthocyanin metabolite branch biosynthesis. Most plant species show limited colour phenotypes due to specific anthocyanin deficiency [2, 3], negatively influencing their ornamental and economic value. However, cinerarias have many colouration patterns including pure colour and bicolour types (Fig. 1a). Most bicolour cineraria exhibit colour differences at the basal section of the ray florets near the stamens and pistils, which is more attractive for pollinators. Thus, the bicolour pattern is more biologically and evolutionarily valued than the pure colour trait. However, the transcriptional mechanisms regulating anthocyanin accumulation and bicolour formation in cineraria are unclear.

Transcription factors (TFs) are key for anthocyanin accumulation as they regulate the anthocyanin biosynthesis pathway (ABP)-related genes, including the MYB-bHLH-WD40 (MBW) complex [4]. In red-centred kiwifruit (*Actinidia chinensis*) [5], apple (*Malus* spp.) [6], *Trifolium repens* [7], monkeyflower (*Mimulus lewisii*) [8], and lily (*Lilium* spp.) [9–11], the MBW complex activates anthocyanin accumulation, and contributes to the formation of differential coloration phenotypes (spotted, blotch, and bicolour traits). Nevertheless, TFs that negatively regulate anthocyanin biosynthesis have also been reported, such as FtMYB18, PtrMYB57, and PpMYB18, which repress anthocyanin accumulation in Tartary buckwheat (*Fagopyrum tataricum*) [12], poplar (*Populus* spp.) [13], and peach (*Prunus persica*) [14], respectively. Additionally, the *Arabidopsis* ANAC032 (No Apical Meristem/NAM, Arabidopsis ATAF1/2, Cup-shaped Cotyledon2/CUC2) TF [15] and *Torenia fournieri* CYCLOIDEA (CYC)-like TF TfCYC2 [16] also negatively regulate anthocyanin biosynthesis.

In eukaryotes, the MADS-box proteins are a large TF family controlling various developmental processes [17–19], including type I and type II MADS-box TFs of higher plants. Type II MADS-box genes can be further grouped as MIKC^C^ and MIKC* type [20, 21]. Moreover, the A-class (*APETALA1*), B-class (*PISTILATA* and *AP3*), C-class (*AGAMOUS*), and E-class (*SEPALLATA*) genes of the MIKC^C^ MADS-box gene family participated in floral organogenesis following the ABCDE model [22, 23]. MADS-box TFs also regulate anthocyanin biosynthesis. In bilberry (*Vaccinium myrtillus*) [24] and lily [25], silencing the SQUAMOSA-like gene *VmTDR4* or the B-class genes *LjLFDEF*, *LjLFGLOA*, and *LjLFGLOB* inhibited anthocyanin biosynthesis and downregulated ABP gene expression. The sweet potato (*Ipomoea batatas*) SQUA subfamily gene *IbMADS10* showed expression patterns similar to those of ABP structural genes, where overexpressing *IbMADS10* induced more anthocyanin accumulation in the callus [26]. However, E-class MADS-box genes related to fruit ripening were identified in sweet cherry, while silencing *PaMADS2*, *PaMADS4*, and *PaMADS7* inhibited fruit ripening and decreased the anthocyanin content [27].

The above examples prove that MADS-box genes positively regulate anthocyanin biosynthesis. Recently, some MADS-box genes that negatively regulate ABP genes were reported. For example, overexpressing *FaMADS1a* reduced the anthocyanin content and the transcript levels of four ABP-related genes (*FaPAL6*, *FaCHS*, *FaDFR*, and *FaANS*) in strawberry (*Fragaria* × *ananassa*) fruits [28]. *AaFUL1* and *AcSVP3* inhibit anthocyanin biosynthesis in anthurium (*Anthurium andraeanum*) [29] and kiwifruit [30], respectively. Moreover, pigmentation fading and organ morphology changes occurred in the corolla after overexpressing *AaFUL1* and *AcSVP3* in tobacco (*Nicotiana tabacum*) [29, 30]. Moreover, subsequent studies in pear (*Pyrus* spp.) [31] and apple [32] demonstrated that the MADS-box genes negatively regulate anthocyanin during fruit colouration. MADS-box TFs also regulate differential colouration patterns in flowers. In *Cattleya hybrida*, the B-class *AP3-1*/*2*/*3*/*4* and *AGL6-2* genes were involved in lip identity, displaying different expression patterns between the yellow hypochile and the purple-red epichile [33].

Cineraria provides a good model for studying anthocyanin biosynthesis and bicolour pattern formation. Previous research measured the pigment composition and content in pure colour cultivars and separated several ABP genes from different cultivars [1]. The competition of *ScF3′H1* (gene encoding flavonoid 3′-hydroxylase), *ScF3′5′H* (gene encoding flavonoid 3′5′-hydroxylase), and *ScDFR1*/2 (gene encoding dihydroflavonol 4-reductase) for naringenin determined the differences in metabolic flux branching of the cyanidin, pelargonidin, and delphinidin pathways. However, *ScCHS2* (gene encoding chalcone synthase), *ScDFR3*, and *ScANS* (gene encoding anthocyanidin synthase) were highly expressed in coloured flowers [1]. Considering the TFs regulating anthocyanin biosynthesis, *ScbHLH17* (subfamily V bHLH TF) was isolated [34]. Indeed, silencing *ScbHLH17* caused loss of anthocyanin accumulation in cineraria leaves, verifying that *ScbHLH17* activated anthocyanin biosynthesis [34]. However, no other TFs were reported, and the molecular mechanism of bicolour formation in cineraria ray florets remained unknown.

We identified two MADS-box genes, *ScAG* and *ScAGL11*, which displayed higher expression levels in the white cultivar and colourless regions of bicolour cultivars. Through gene expression, phylogenetic, gene overexpression, and virus-induced gene silencing (VIGS) analyses, this study verified *ScAG* and *ScAGL11* as negative regulators of anthocyanin biosynthesis, which influence bicolour pattern formation. The regulatory mechanisms of *ScAG* and *ScAGL11* in anthocyanin biosynthesis and bicolour pattern formation are discussed herein.

## Results

### Pigment composition and epidermal structure analysis in pure colour and bicolour cineraria cultivars

The anthocyanin content and composition of four pure colour ‘Jester’ cultivars, comprising blue (JeB), carmine (JeC), pink (JeP) and white (JeW), were taken from a previous study [1]. Thus, this study conducted high-performance liquid chromatography-ion trap mass spectrometry (HPLC-MS) experiments only in two bicolour cultivars [pink bicolour (JePB) and carmine bicolour (JeCB)] ray florets at Stage (S) 5. The pigment derivatives were identified by comparing the retention time, absorption spectra, and mass spectrum characteristics from previous literature with the data of this study (Supplementary Data Table S1; Fig. 1b). The coloured region of JeCB had two cyanidin (peaks C1 and C2) and one delphinidin (peak C3) derivative (Supplementary Data Table S1; Fig. 1b). The cyanidin derivative content was 90% of the total anthocyanins (Fig. 1c). For the coloured region of JePB, three pelargonidin (peaks P1, P2, and P4) derivatives and one cyanidin (peak P3) derivative were identified (Supplementary Data Table S1; Fig. 1b). The pelargonidin derivative content was 81.5% of the total amount of anthocyanins (Fig. 1c). The composition and the content ratio of pigments in the coloured region of bicolour cultivars were consistent with previously investigated pure colour cultivars [1]. Moreover, the colourless region of bicolour cultivars had undetectable anthocyanin derivatives (Fig. 1b and c), confirming that anthocyanins only accumulated in the coloured regions of bicolour cultivars.

In some plants, such as lily, parenchymal and epidermal cell differences cause anthocyanin accumulation in the raised spots, revealing that the shape of petal epidermal cells is also connected to flower colour and bicolour formation [35]. We investigated the epidermal structure of pure colour and bicolour ray florets at S5 under an optical microscope. The JeB, JeC, JeP, JeW, coloured, and colourless regions of JeCB and JePB showed no anatomical structural differences in the upper and lower epidermis of ray florets, regardless of anthocyanin accumulation (Supplementary Data Fig. S1a and b). Then, the epidermal cells of JeCB ray florets at S5 were observed under a scanning electron microscope (SEM) to explore if cell morphology influences bicolour pattern formation. The 3D structure of ray floret cells showed no differences in cell shape between the colourless and coloured regions of the upper or lower epidermis (Fig. 1d). This observation indicates that the epidermal cell structure did not influence anthocyanin accumulation or bicolour pattern formation.

### Transcriptome sequencing and differentially expressed gene analysis

We constructed transcriptome databases of coloured and colourless regions of JePB ray florets at S2 with three biological replicates. The study quantified transcript accumulation levels to identify the genes involved in anthocyanin accumulation and bicolour formation in cineraria. Reads from the coloured and colourless regions were assembled into 213 951 transcripts obtained from *de novo* assembly using Trinity (Supplementary Data Tables S2 and S3). A total of 65 144 unigenes from two regions were annotated to different functions according to 13 annotated databases (Supplementary Data Table S4, Supplementary Data Fig. S2a–c). Then, 2246 differentially expressed genes (DEGs) between two regions were identified, with 949 downregulated genes and 1297 upregulated genes in the colourless region (Supplementary Data Fig. S2d–f). Moreover, 2246 DEGs were annotated to at least one database out of the NR, TmHMM, SignalP, Prot, RNAMMER, BLASTP, Map, BLASTX, eggNOG, Map, PFAM, and BLASTX databases (Supplementary Data Fig. S2g–h). Next, 386 of the 2246 DEGs were considered key since accumulation of their transcripts between the colourless and coloured regions exceeded 10-fold (Supplementary Data Table S5).

Four ABP structural genes (*ScCHS2*, *ScF3H1*, *ScDFR3*, and *ScAN*S) were selected from the 386 key DEGs as their transcripts highly accumulated in the coloured regions (Table 1). However, none of the MYB genes important in ABP was differentially expressed. Besides, only a bHLH TF ScbHLH17 (TRINITY_DN9621_c1_g1) was highly expressed in the coloured regions (Table 1). Interestingly, two annotated MADS-box genes, TRINITY_DN18674_c1_g1 (designated *ScAG*) and TRINITY_DN20125_c0_g1 (designated *ScAGL11*), identified from the 386 key DEGs, were significantly expressed in the colourless region (Table 1). Subfamilies 4, 5, 6, and 7 of MYB TFs and subfamilies II and V of the bHLH TFs are mainly related to flavonoid synthesis [4]. A previous study isolated eight *ScMYB*s in subfamilies 4, 5, 6, and 7, three *ScbHLH*s (subfamilies II and V), and nine ABP structural genes [1]*.* Therefore, we chose all 27 genes (13 ABP structural genes, *ScAG*, *ScAGL11*, 8 *ScMYB*s and 4 *ScbHLH*s) for selecting the candidate genes.

**Table 1:** Key genes with different transcript accumulation levels in coloured and colourless regions.

		FPKM		Log_2_		
Gene	Unigene	JePB-W	JePB-C	fold change	Up/down	BLAST top hit
*ScCHS2*	TRINITY_DN11487_c0_g1	187	10 632	−5.885	Down	Chalcone synthase type 4 (*Dahlia pinnata*)
*ScF3H1*	TRINITY_DN15133_c1_g1	464	13 445	−4.858	Down	Flavanone 3-hydroxylase (*Gynura bicolor*)
*ScDFR3*	TRINITY_DN17756_c4_g5	84	3567	−5.464	Down	Dihydroflavonol reductase (*Gynura bicolor*)
*ScANS*	TRINITY_DN19492_c0_g2	36	2245	−5.061	Down	Anthocyanidin synthase (*Pericallis cruenta*)
*ScbHLH17*	TRINITY_DN9621_c1_g1	165	1932	−3.550	Down	bHLH transcription factor 2 (*Chrysanthemum* × *morifolium*)
*ScAG*	TRINITY_DN18674_c1_g1	265	1	8.050	Up	Floral homeotic protein AGAMOUS (*Lactuca sativa*)
*ScAGL11*	TRINITY_DN20125_c0_g1	33	0.08	8.688	Up	Floral homeotic protein AGAMOUS-like isoform X1 (*L. sativa*)

JePB-W, colourless region of JePB; JePB-C, coloured region of JePB. Fold change, ratio of gene FPKM values in JePB-W compared with JePB-C. The expression levels of the genes listed above were at least 10 times different between colourless and coloured regions (|Log_2_FoldChange| ≥ Log_2_10). All data are presented as the mean from three biological replicates.

Ray florets of pure colour and bicolour cultivars at S1 and S2 were used as materials for reverse transcription PCR (RT–PCR) to measure the expression levels of the above 27 genes. The results showed that among the 13 ABP structural genes, *ScCHS2*, *ScF3H1*, *ScDFR3*, and *ScANS* exhibited low expression levels in the colourless regions of JePB, JeCB, and JeW, and higher expression in the coloured regions of JeCB, JePB, JeB, JeC, and JeP (Fig. 2a). Only one bHLH gene (*ScbHLH17*) showed a high expression level in the coloured regions of two bicolour cultivars and three pure coloured cultivars (Fig. 2a). Additionally, *ScAG* was expressed in the colourless regions of JePB, JeCB (S1 and S2), and JeW (Stage 2), while *ScAGL11* was highly expressed in the colourless regions of JePB and JeCB at Stages 1 and 2. The transcriptional levels of *ScAG* and *ScAGL11* were very low in the coloured regions of JeCB, JePB, JeB, JeC, and JeP (Fig. 2b).

**Figure 2:**
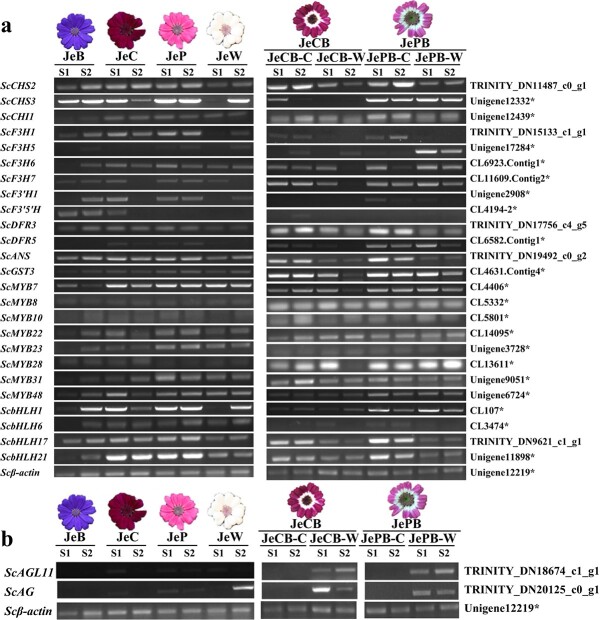
Expression of candidate genes for anthocyanin biosynthesis and bicolour formation. **a** RT–PCR expression of 13 structural genes, 8 *ScMYB*s (subfamilies 4, 5, 6, and 7), and 4 *ScbHLH*s (subfamilies II and V) in pure colour cultivars and the coloured and colourless regions of bicolour cultivars. **b** RT–PCR expression of *ScAG* and *ScAGL11* in pure colour cultivars and the coloured and colourless regions of bicolour cultivars. *Scβ-actin* was used to normalize gene expression. *These genes were isolated from a previous study [1]. **c** qRT–PCR analysis of key DEGs (*ScCHS2*, *ScF3H1*, *ScDFR3*, *ScANS*, *ScbHLH17*, *ScAG*, and *ScAGL11*) in coloured and colourless regions of JeCB and JePB at five stages. All data are mean ± standard deviation of three biological replicates. ^*^P < .05, ^**^P < .01; Student’s *t*-test. JePB-C, coloured region of JePB; JePB-W, colourless region of JePB; JeCB-C, coloured region of JeCB; JeCB-W, colourless region of JeCB.

**Figure 2:**
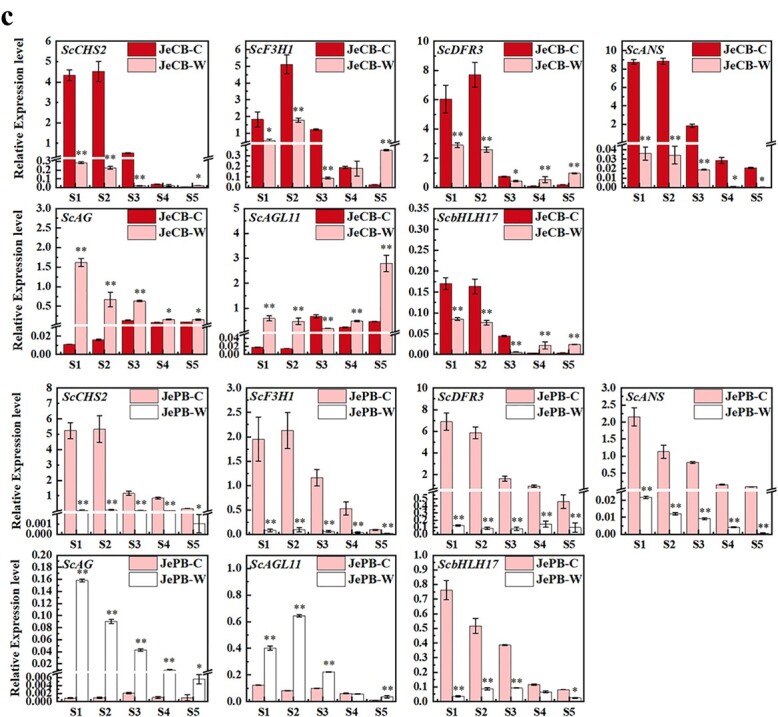
Continued.

We further checked the qRT–PCR expression of the above candidate genes (*ScCHS2*, *ScF3H1*, *ScDFR3*, *ScANS*, *ScbHLH17*, *ScAG*, and *ScAGL11*) in ray florets from the five stages of bicolour cultivars (JeCB and JePB). *ScCHS2*, *ScF3H1*, *ScDFR3*, *ScANS*, and *ScbHLH17* were highly expressed in the coloured region at different stages. Specifically, *ScCHS2*, *ScF3H1*, *ScDFR3*, and *ScbHLH17* were highly expressed at S1–S3 and *ScANS* at all stages (Fig. 2c). On the contrary, *ScAG* and *ScAGL11* showed higher expression in the colourless region, especially at S1 and S2 (Fig. 2c). Moreover, the expression of these seven genes in JePB at S2 was consistent with the DEG data (Supplementary Data Fig. S3), indicating that the gene expression analysis and high-throughput sequencing technology were both relevant, repeatable, and reliable.

### Hereditary analysis and expression verification of *ScAG* and *ScAGL11* in the hybrid (*F*_1_) population

The study analysed the flower phenotype of the *F*_1_ population derived from JeW × JeCB crosses to explore the hereditary characteristics of the bicolour trait and pure colour trait in cineraria. Forty-seven of the 65 *F*_1_ plants developed carmine bicolour phenotypes, and the remaining 18 *F*_1_ plants (Supplementary Data Fig. S4) exhibited pure carmine phenotypes. The *F*_1_ plants with bicolour phenotypes or pure colour phenotypes showed a 2.61:1 segregation ratio, failing to fit the 1:1 ratio but close to the 3:1 segregation ratio, indicating that at least two independent genes control the bicolour trait.

**Figure 3:**
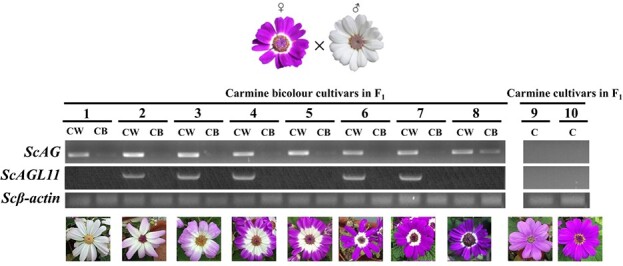
Expression of *ScAG* and *ScAGL11* in coloured and colourless regions of 10 *F*_1_ plants. *F*_1_ plants were derived from a cross between JeW and JeCB. Carmine bicolour flowers with different colourless areas chosen from the *F*_1_ plants are numbered 1–8, and pure carmine flowers are numbered 9 and 10. *Scβ-actin* was used to normalize gene expression. The pictures present the phenotype of cineraria capitulum at Stage 5. The ray florets of cineraria at Stage 2 were used for RT–PCR analysis. CW, colourless region in ray florets; CB, coloured region in ray florets; C, pure carmine ray florets.

We randomly selected 10 *F*_1_ plants, including 8 carmine bicolour progenies and 2 pure carmine progenies, to check the expression of *ScAG* and *ScAGL11* and verify the correlation between the colouration pattern and candidate gene expression in *F*_1_ plants. The results showed that *ScAG* and *ScAGL11* were only expressed in the colourless regions of bicolour progenies and not in pure carmine progenies (Fig. 3). Thus, *ScAG* and *ScAGL11* are closely associated with the bicolour trait.

### Sequence alignment and bioinformatics analysis of proteins ScAG and ScAGL11

**Figure 4:**
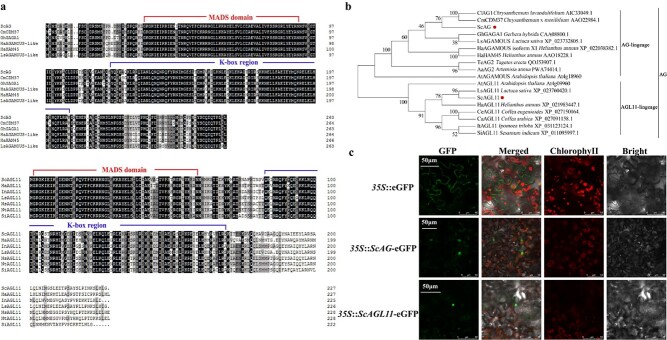
*ScAG* and *ScAGL11* sequence alignment and bioinformatics analysis. **a** ScAG and ScAGL11 amino acid sequence alignments and motif analysis. The MADS domain and the K-box region are highlighted with red and blue lines. Sc, *S. cruentus*; Cm, *Chrysanthemum* × *morifolium*; Gh, *Gerbera hybrida*; Ha, *Helianthus annuus*; Ls, *Lactuca sativa*; In, *Ipomoea nil*; Ns, *Nicotiana sylvestris*; Nt, *Nicotiana tomentosiformis*; Si, *Sesamum indicum*. **b** Amino acid sequences of ScAG and ScAGL11 and 16 MADS-box genes were used to build a phylogenetic tree based on maximum likelihood. The red circle indicates ScAG and ScAGL11. **c** ScAG and ScAGL11 subcellular localization in *N. benthamiana* leaves. A microscope imaged the green GFP fluorescence signal 48 hours after injection. GFP, GFP channel; Merged, merged image of GFP and bright-field channels; ChlorophyII, image of red chloroplast autofluorescence; Bright, light microscopy image.

We isolated full-length cDNA sequences of *ScAG* and *ScAGL11* in the JeCB and JeC ray florets. The *ScAG* open reading frame (ORF) was 795 bp, encoding a 264 amino acid protein, and *ScAGL11* had a 684-bp ORF encoding a 227 amino acid protein. No differences were observed in the ScAG or ScAGL11 amino acid sequences of the JeCB and JeC ray florets (Supplementary Data Fig. S5). Multiple sequence alignment revealed that ScAG and ScAGL11 have two conserved motifs, the MADS domain and the K-box region (Fig. 4a). The former controls sequence-specific DNA binding and MADS-box protein dimerization, while the latter controls protein molecular interactions, thus identifying the genes as MIKC-type MADS-box TFs (Fig. 4a). Furthermore, ScAG and ScAGL11 were used to construct a phylogenetic tree with 39 *Arabidopsis* MIKC-type MADS-box TFs (Supplementary Data Table S6). Consequently, ScAG and ScAGL11 clustered with the *Arabidopsis* AG subfamily (C-class MADS-box) (Supplementary Data Fig. S6). Another phylogenetic tree constructed using 16 MADS-box-related TFs derived from other species with ScAG and ScAGL11 indicated that ScAG clusters in the AG lineage with high similarity to CmCDM37 (*Chrysanthemum* × *morifolium*) and ClAG1 (*Chrysanthemum lavandulifolium*). ScAGL11 clustered into the AGL11 lineage with high similarity to *Lactuca sativa* LsAGL11 (Fig. 4b).

We detected the subcellular localization of the ScAG and ScAGL11 proteins to confirm the putative protein function of the two TFs. Two recombinant vectors, *35S*::*ScAG*-eGFP and *35S*::*ScAGL11*-eGFP, were constructed and separately introduced into *Nicotiana benthamiana* leaves with the empty *35S*::eGFP vector serving as the negative control. The results showed that the GFP fluorescence signals of *35S*::*ScAG*-eGFP and *35S*::*ScAGL11*-eGFP were strongly detected in the nucleus of *N. benthamiana* cells (Fig. 4c). In contrast, the signal was distributed in the nucleus and cytomembrane of the negative control (Fig. 4c), indicating that ScAG and ScAGL11 were nucleus-localized proteins.

### *ScAG* and *ScAGL11* regulate anthocyanin biosynthesis in tobacco flowers and cineraria leaves.

**Figure 5:**
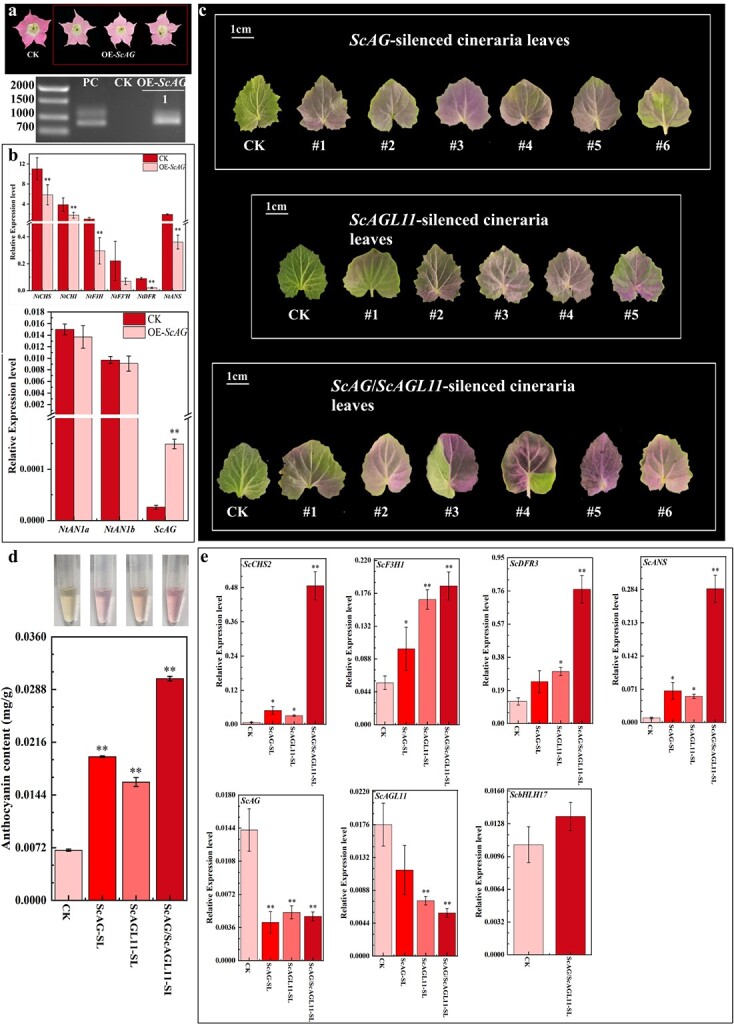
Functional verification of *ScAG* and *ScAGL11* involved in anthocyanin biosynthesis in tobacco flowers and cineraria leaves. **a**, **b** Overexpression of *ScAG* in tobacco. **a** Flower phenotypes and gene (*ScAG*) expression analysis in CK and OE-*ScAG* lines. **b** qRT–PCR analysis for detecting the expression of ABP structural (*NtCHS*, *NtCHI*, *NtF3H*, *NtF3′H*, *NtDFR*, and *NtANS*) and regulatory (*NtAN1a*, *NtAN1b*, and *ScAG*) genes in CK and OE-*ScAG* tobacco corolla. CK, transgenic empty pBI121 vector tobacco; OE-*ScAG*, *ScAG*-overexpressing tobacco; PC, positive control, *Agrobacterium tumefaciens* strain GV3101 containing *35S*::*ScAG*-pBI121 vector. **c**–**e** Silencing *ScAG* and *ScAGL11* in JeP leaves by the VIGS system. **c** Phenotypes of CK and *ScAG*-, *ScAGL11*-, and *ScAG*/*ScAGL11*-silenced cineraria leaves. **d** Anthocyanin content of CK and *ScAG*-, *ScAGL11*-, and *ScAG*/*ScAGL11*-silenced cineraria leaves. **e** qRT–PCR analysis for detecting the expression of ABP genes (*ScCHS2*, *ScF3H1*, *ScDFR3*, *ScANS*, *ScbHLH17*, *ScAG*, and *ScAGL11*) in CK and *ScAG*-, *ScAGL11*-, and *ScAG*/*ScAGL11*-silenced cineraria leaves. CK, leaves infiltrated with empty pTRV1 and pTRV2 vectors; *ScAG*-SL, *ScAG*-silenced cineraria leaves; *ScAGL11*-SL, *ScAGL11*-silenced cineraria leaves; *ScAG*/*ScAGL11*-SL, leaves of *ScAG* and *ScAGL11* were silenced together. All data are mean ± standard deviation from three biological replicates. ^*^P < .05, ^**^P < .01; Student’s *t*-test.

*ScAG* and *ScAGL11* were overexpressed in tobacco to determine whether *ScAG* and *ScAGL11* are involved in the ABP. The corolla of OE-*ScAG* tobacco lines changed to pale pink from red (Fig. 5a), while the corolla colour of OE-*ScAGL11* lines changed negligibly (Supplementary Data Fig. S7). Furthermore, gene expression analysis of the OE-*ScAG* corolla verified that tobacco ABP structural genes *NtCHS*, *NtCHI*, *NtF3H*, *NtDFR*, and *NtANS* decreased significantly compared with control (CK, transgenic empty pBI121 vector tobacco) lines (Fig. 5b). In the previous study, *ScbHLH17* regulated anthocyanin accumulation in cineraria by influencing the expression of ABP genes [34]. Since two bHLH TFs, NtAN1a and NtAN1b, were proved to enhance anthocyanin accumulation in tobacco [36], we measured the expression of *NtAN1a* and *NtAN1b* in OE-*ScAG* tobacco lines to explore if *ScAG* and *ScAGL11* regulate anthocyanin accumulation through bHLH TFs. However, the transcript levels of two bHLH genes *NtAN1a* and *NtAN1b* exhibited no significant change between the OE-*ScAG* and CK (Fig. 5b) lines. Thus, it appeared that *ScAG* might negatively influence anthocyanin accumulation; but since the colour of the OE-*ScAG* tobacco corolla only turned pale but did not disappear completely, more substantial evidence is needed to support the above suggestion.

**Figure 6:**
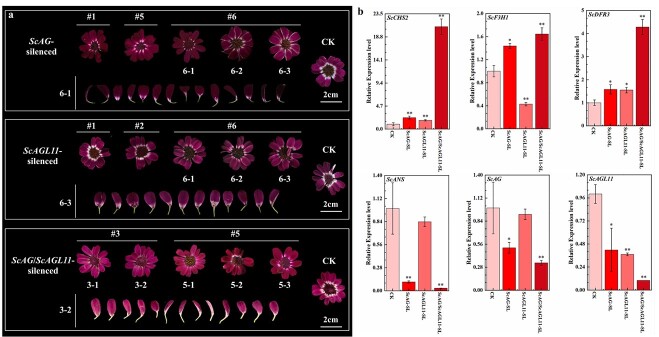
Functional verification of *ScAG* and *ScAGL11* in the JeCB capitulum. **a** Phenotypes of CK and *ScAG*-, *ScAGL11*-, and *ScAG*/*ScAGL11*-silenced capitulum. **b** qRT–PCR detection of the ABP genes (*ScCHS2*, *ScF3H1*, *ScDFR3*, *ScANS*, *ScAG*, and *ScAGL11*) in CK and *ScAG*-, *ScAGL11*-, and *ScAG*/*ScAGL11*-silenced cineraria ray florets. CK, capitulum infiltrated with empty pTRV1 and pTRV2 vectors; *ScAG*-SL, *ScAG*-silenced capitulum; *ScAGL11*-SL, *ScAGL11*-silenced capitulum; *ScAG*/*ScAGL11*-SL, capitulum in which *ScAG* and *ScAGL11* were silenced together. The data are mean ± standard deviation from three biological replicates. ^*^P < .05, ^**^P < .01; Student’s *t*-test.

The VIGS system was used to silence *ScAG* and *ScAGL11* in JeP leaves at the two-leaf stage to further identify the functions regulating anthocyanin biosynthesis in cineraria. First, 178- and 179-bp fragments from *ScAG* and *ScAGL11* non-conserved regions near the 3*′-*UTR were cloned and inserted into Tobacco rattle virus (TRV) constructs (Supplementary Data Figs S8 and S11c). Finally, we obtained *ScAG*-, *ScAGL11*- and *ScAG*/*ScAGL11*-silenced (*ScAG* and *ScAGL11* silenced together) cineraria lines (Fig. 5c, Supplementary Data Fig. S9a and b). The leaves of CK (leaves infiltrated with empty pTRV1 and pTRV2 vectors) lines remained green, while the leaves of silenced lines turned purple, especially the *ScAG*/*ScAGL11*-silenced leaves (Fig. 5c). The anthocyanin content of the *ScAG*- (2.88-fold), *ScAGL11*- (2.375-fold) and *ScAG*/*ScAGL11*-silenced (4.41-fold) leaves increased significantly, especially the *ScAG*/*ScAGL11*-silenced lines compared with CK (Fig. 5d).

qRT–PCR methods were used to determine the expression of anthocyanin biosynthesis-related genes in silenced leaves. *ScAG* in *ScAG*-silenced leaves was 70% downregulated compared with CK. In *ScAGL11-*silenced leaves, *ScAGL11* was 56% downregulated. In *ScAG/ScAGL11-*silenced leaves, *ScAG* and *ScAGL11* were downregulated by 67 and 68%, respectively (Fig. 5e), confirming that the target genes were successfully silenced in cineraria leaves. The expression of four key ABP structural genes (*ScCHS2*, *ScF3H1*, *ScDFR3* and *ScANS*) was significantly increased in *ScAG*-, *ScAGL11*- and *ScAG*/*ScAGL11*-silenced leaves compared with CK lines, particularly the *ScAG*/*ScAGL11*-silenced leaves (Fig. 5e). However, *ScbHLH17* showed an insignificant difference (Fig. 5e). Therefore, *ScAG* and *ScAGL11* probably inhibit anthocyanin accumulation and the expression of ABP structural genes, while *ScbHLH17* is possibly not an *ScAG* and *ScAGL11* downstream gene.

### *ScAG* and *ScAGL11* are involved in bicolour pattern formation in the cineraria capitulum

The VIGS experiment produced *ScAG*-, *ScAGL11*-, and *ScAG*/*ScAGL11*-silenced (*ScAG* and *ScAGL11* silenced together) capitulum to confirm the role of *ScAG* and *ScAGL11* in the cineraria bicolour pattern formation (Fig. 6a; Supplementary Data Fig. S9c and d). The colourless regions of *ScAG*-, *ScAGL11*-, and *ScAG*/*ScAGL11*-silenced ray florets decreased (silencing effect) to different degrees (Supplementary Data Fig. S10). Moreover, the silencing effect appeared more strongly in *ScAG*- than *ScAGL11*-silenced ray florets, but *ScAG*/*ScAGL11*-silenced ray florets showed the most obvious silencing phenotype as the colourless region significantly decreased (Fig. 6a; Supplementary Data Fig. S10). We then detected the expression of anthocyanin biosynthesis-related genes in all silenced plants. In the *ScAG-*silenced ray florets, *ScAG* expression was 51% downregulated compared with CK (capitulum infiltrated with empty pTRV1 and pTRV2 vectors) lines. In the *ScAGL11*-silenced ray florets, *ScAGL11* expression was 78.5% downregulated. In *ScAG*/*ScAGL11*-silenced ray florets, the expression of *ScAG* and *ScAGL11* was downregulated by 66.7 and 90%, respectively (Fig. 6b). The expression of the structural genes *ScCHS2* and *ScDFR3* was significantly upregulated in the *ScAG*- (2.34- and 1.58-fold), *ScAGL11*- (1.79- and 1.55-fold), and *ScAG*/*ScAGL11*- (20.89- and 4.28-fold) silenced ray florets compared with CK, especially in the *ScAG*/*ScAGL11*-silenced ray florets. In contrast, the expression of *ScF3H1* dramatically increased in the *ScAG*- and *ScAG*/*ScAGL11*-silenced ray florets compared with the CK line (Fig. 6b). The above findings showed that *ScAG* and *ScAGL11* probably control bicolour pattern formation and negatively regulate the expression of *ScCHS2* and *ScDFR3*. In addition, *ScAG* might inhibit the expression of *ScF3H1* independently.

### Protein interaction relationship between ScAG and ScAGL11

**Figure 7:**
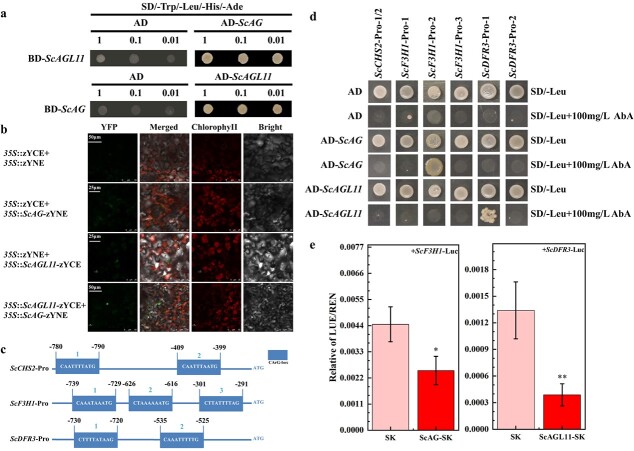
ScAG interacted with ScAGL11 and directly regulated the expression of *ScF3H1* and *ScDFR3* by binding to their promoters. **a** Y2H assay of protein interaction between ScAG and ScAGL11. Numbers 1, 0.1, and 0.01 indicate dilution multiples of the bacterial liquid for 1×, 10× and 100×, respectively. AD, pGADT7 vector. **b** A BiFC assay confirmed the interaction between ScAG and ScAGL11 in *N. benthamiana* leaves. A microscope imaged the YFP fluorescence signal 48 hours after injection. **c** Sequences and positions of the CArG boxes in the *ScCHS2*, *ScF3H1*, and *ScDFR3* promoters. Different CArG boxes are numbered 1, 2, or 3, and the black numbers indicate respective fragment positions from the ATG start codon. **d** Combination of *ScAG*/*ScAGL11* with *ScCHS2*, *ScF3H1*, and *ScDFR3* promoters in the Y1H system. The prey vector AD-*ScAG* or AD-*ScAGL11* was transformed into Y1H Gold cells harbouring pAbAi recombination vectors, which contained the shortened promoter fragments. *ScCHS2*-pro1/2 means the pAbAi vector contained fragments 1 and 2 of the CArG box in the *ScCHS2* promoter. **e** The LUC assay showed that *ScAG* inhibited *ScF3H1* promoter activity, and *ScAGL11* inhibited *ScDFR3* promoter activity. SK, leaves infiltrated with empty SK vector. All data are mean ± standard deviation from three biological replicates. ^*^P < .05, ^**^P < .01; Student’s *t*-test.

The above results showed that the silencing effects were more evident in the *ScAG*-/*ScAGL11*- than in the *ScAG*- and *ScAGL11*-silenced lines. Considering MADS-box TFs function as dimer proteins [19], we performed yeast two-hybrid (Y2H) and bimolecular fluorescence complementation (BiFC) assays to test whether ScAG interacts with ScAGL11 *in vivo*. In the Y2H assay, the ORFs of *ScAG* and *ScAGL11* were inserted into the pGBKT7 (BD) and pGADT7 (AD) vectors. We observed that yeast cells co-transformed with the AD-*ScAGL11*/BD-*ScAG* or AD-*ScAG*/BD-*ScAGL11* recombinant vectors grew on SD/−Trp/−Leu/-His/−Ade screening medium, whereas yeast cells co-transformed with AD/BD-*ScAG* or AD/BD-*ScAGL11* could not grow (Fig. 7a), suggesting that ScAG interacts with ScAGL11 in the yeast system.

The BiFC assay was conducted to confirm the interaction between ScAG and ScAGL11. The *ScAG* and *ScAGL11* ORFs (without the stop codon) were separately linked to the N-terminal or C-terminal regions of yellow fluorescent protein (YFP). The YFP fluorescence signal was observed in *N. benthamiana* leaves co-transformed with *35S*::*ScAGL11*-zYCE and *35S*::*ScAG*-zYNE recombinant vectors (Fig. 7b). However, no signal was detected in the negative control leaves co-transformed with *35S*::zYCE/*35S*::zYNE, *35S*::zYCE/*35S*::*ScAG*-zYNE, and *35S*::zYNE/*35S*::*ScAGL11*-zYCE (Fig. 7b). The results of BiFC and Y2H suggested an interaction between ScAG and ScAGL11 *in vivo*.

### *ScAG* and *ScAGL11* directly regulate anthocyanin-related structural genes

Because the transcript accumulation of *ScCHS2*, *ScF3H1*, and *ScDFR3* significantly increased in the silenced cineraria leaves and capitulum, the study explored whether *ScAG* and *ScAGL11* directly regulate *ScCHS2*, *ScF3H1*, and *ScDFR3* transcription. Since MADS-box proteins regulate gene expression by binding the CArG box in the promoters [28], the promoter sequences of *ScCHS2*, *ScF3H1*, and *ScDFR3* were cloned from the DNA of JeCB ray florets (Supplementary Data Table S7). The *cis*-elements in promoters were analysed using the PlantCARE database and we found that the *ScCHS2* (2 CArG boxes), *ScF3H1* (3 CArG boxes), and *ScDFR3* (2 CArG boxes) promoters had several CArG boxes (Fig. 7c; Supplementary Data Tables S8–S10). Shortened fragments containing one or two CArG boxes of the *ScCHS2*, *ScF3H1*, and *ScDFR3* promoters were separately inserted into the pAbAi vectors (Supplementary Data Fig. S11f). The recombination vectors were used for yeast one-hybrid (Y1H) transformation. The Y1H assay results showed that only yeast cells containing the AD-*ScAG*/pAbAi-*ScF3H1*-pro-2 and AD-*ScAGL11*/pAbAi-*ScDFR3*-pro-1 vectors grew on the SD/−Leu/100 mg L^−1^ AbA screening medium, In contrast, the control groups (yeast cells containing empty pGADT7 vector with pAbAi recombination vectors) failed to grow (Fig. 7d). These results showed that the ScAG protein binds to the CArG box in the *ScF3H1* promoter, and the ScAGL11 protein binds to the CArG box in the *ScDFR3* promoter.

A dual-luciferase (LUC) transient expression assay was performed in *N. benthamiana* leaves against the control group. The LUC/REN (*Renilla* luciferase) ratio in *N. benthamiana* leaves co-transformed with SK-*ScAG*/Luc-*ScF3H1* and SK-*ScAGL11*/Luc-*ScDFR3* significantly decreased (Fig. 7e). Y1H and LUC transient expression assays revealed that *ScAG* inhibits *ScF3H1* transcription by binding to its promoter, and *ScAGL11* inhibits *ScDFR3* transcription in the same way.

## Discussion

Flower colour formation is usually due to the expression of the anthocyanin-related MBW complex [4]. However, this study performed a comparative transcriptome analysis of two selected MADS-box genes, *ScAG* and *ScAGL11*, which were highly abundant in the colourless region and low in the coloured region. Moreover, independently inhibiting *ScAG* and *ScAGL11* expression or silencing both genes together in cineraria leaves and capitulum enhanced the anthocyanin content and the disappearance of the bicolour phenotype. Besides, the increased expression of ABP structural genes reveals the critical role of *ScAG* and *ScAGL11* in the regulatory mechanism of anthocyanin biosynthesis and bicolour pattern formation in cineraria. Several plants, including strawberry [28], anthurium [29], and kiwifruit [30], have MADS-box genes that negatively regulate anthocyanin accumulation.

Moreover, the Y2H and BiFC results of this study demonstrated that ScAG interacts with ScAGL11. Some studies showed that MADS-box TFs function as heterodimers to co-regulate anthocyanin or carotenoid biosynthesis. For example, CsMADS5 interacts with CsMADS6 to enhance carotenoid accumulation in citrus (*Citrus* spp.) [37, 38]. Two C-class MADS-box genes, *pMADS3* and *FBP6*, were characterized in petunia (*Petunia hybrida*) when *pMADS3* and *FBP6* were independently silenced. The result showed few changes in the flower organs, but silencing *pMADS3* and *FBP6* together caused double flower formation and pigmentation fading [39]. In *Gerbera hybrida*, silencing the SEP subfamily (*GRCD5*) of ray florets inhibited anthocyanin biosynthesis, and when *GRCD4* and *GRCD5* were silenced together anthocyanins did not accumulate in ray florets [40]. We hypothesized that ScAGL11 synergistically enhances the complex with ScAG, since silencing *ScAG* and *ScAGL11* together caused stronger phenotypic changes in both leaves and capitulum of cineraria. Moreover, silencing *ScAGL11* in cineraria affected *ScAG* expression as in lily because suppressing the B-class MADS-box gene *LFDEF* decreased the expression of two other B-class MADS-box genes, *LFGLOA* and *LFGLOB* [25]. Whether these results are due to the protein interaction or the autoregulatory feedback system of MADS-box genes requires further study.

The MBW complex is a major part of the regulatory network determining anthocyanin biosynthesis [41]. Previous studies showed that MADS-box TFs regulate anthocyanin biosynthesis by influencing MYB gene transcription. In bilberry fruits, silencing *VmTDR4* resulted in lower anthocyanin biosynthesis-related R2R3 MYB genes [24]. However, overexpressing the SVP-like MADS-box gene *AcSVP3* in kiwifruit and tobacco flowers significantly downregulated the transcription of the R2R3 MYB genes *AcMYB110a* and *NtAN2* accompanied by the flower fading phenotype [30]. These observations indicate that MADS-box genes are upstream regulatory factors of the MYB gene.

Nevertheless, no MYB gene from this study displayed a differential expression pattern in the coloured and colourless regions. The transcript level of *ScbHLH17*, which correlates with anthocyanin accumulation [34], showed no significant change between *ScAG*/*ScAGL11*-silenced leaves and the CK lines. Recent studies established that MADS-box genes directly regulate the expression of the ABP structural gene instead of going through the MBW complex. Moreover, overexpressing *FcMADS9* upregulates *MdCHS*, *MdCHI*, *MdDFR*, *MdANS*, and *MdUFGT* expression in apple [42]. *FaCHS*, *FaDFR*, and *FaANS* showed lower expression and pigment accumulation in *FaMADS1a*-overexpressed fruits than in control fruits of strawberry [28]. In cherry fruit, silencing *PaMADS7* enhanced the transcription of *PaCHS*, *PaANS*, and *PaDFR* [27]. A regulatory model where TFs directly regulate structural genes has been confirmed in strawberry [43], tomato (*Solanum lycopersicum*) [44], and lychee (*Litchi chinensis*) [45]. A similar regulatory mechanism was observed in this study, as the expression of the ABP structural genes, *ScCHS2* and *ScDFR3*, were upregulated in the *ScAGL11*-silenced capitulum. Besides, the expression of *ScCHS2*, *ScDFR3*, and *ScF3H1* was upregulated in the *ScAG*- and *ScAG*/*ScAGL11*-silenced capitulum. The Y1H and dual-luciferase assays demonstrated that *ScAG* and *ScAGL11* separately inhibit the transcriptional activity of *ScF3H1* and *ScDFR3* by recognizing and binding to the CArG elements in the promoters, hence reducing anthocyanin accumulation.

Interestingly, besides negatively regulating anthocyanin biosynthesis, *ScAG* and *ScAGL11* also influence bicolour pattern formation in cineraria ray florets. Flower colour spots form many ornamental patterns, such as splatter spots [9], blotch [46], and bicolour flowers [47], and can be divided into regular and irregular types. Studies have found that transposon, DNA methylation, RNA interference, and virus infection possibly cause irregular flower spots in petunia [48], *Oncidium hybridum* [49], peach [50], and *Dahlia pinnata* [51], respectively. The irregular types were variable, and their heredity did not follow Mendel’s law of inheritance. Regular types can be stably inherited across generations through genes. In recent years, hybrid experiments in lily [35], *Clarkia gracilis* [52], and monkeyflower [53] showed that the genetic segregation of hybrid progenies presents a classic Mendelian segregation ratio, indicating that a single gene controls regular colour traits. Through heredity analysis, the segregation ratio of bicolour and pure colour progenies was 2.6:1, suggesting that the cineraria bicolour trait was regular and might be controlled by at least two genes. Further expression analysis showed that *ScAG* and *ScAGL11* were co-expressed with the bicolour trait. Silencing *ScAG* and *ScAGL11* in cineraria caused a disappearance of the colourless region, and transformed the bicolour flower into a pure-colour flower.

Recent studies reported MYB genes for determining the differential colouration formation [54]. *LhMYB12*-*Lat*, a novel allele of *LhMYB12* in lily, co-segregated with the splatter phenotype, indicating its function in splatter-type spot formation [10]. Nonetheless, *cis*-regulatory changes in the promoters of *CgMyb1* allele produced different locations of spots [52, 55]. However, no novel *ScAG* and *ScAGL11* alleles were identified in bicolour cineraria. The *ScAG* and *ScAGL11* amino acid sequences were identical between bicolour and pure colour ray florets. Therefore, other factors probably impact this trait besides the sequence difference in the gene-coding region that determines bicolour formation. For example, bicolour pattern formation in lily is due to the transcript-derived small interfering RNA (siRNA) miR828 accumulating in the colourless region and suppressing *MYB12* transcription [56]. Further studies of siRNA or other regulators influencing *ScAG* and *ScAGL11* transcript accumulation are needed to illustrate the molecular mechanism of bicolour pattern formation in cineraria.

Phylogenetic analysis showed that *ScAG* and *ScAGL11* belonged to the AGAMOUS MADS-box subfamily. The original function of this subfamily is to regulate stamen and pistil differentiation, a critical role in the multiple flower formation of ornamental plants [57]. For example, the gerbera *GhGAGA* gene, having a close evolutionary relationship with *ScAG*, controls carpel and stamen identity [58]. However, in this study, *ScAG* and *ScAGL11* were involved in the anthocyanin biosynthesis pathway, but no ectopic organ identity was discovered in *ScAG*- and *ScAGL11*-overexpressing or -silenced lines. We propose that the main reason behind this trait is the functional divergence of the AGAMOUS family. The AGAMOUS family has functional diversification via duplication events [59]. For example, it regulates pollen development (*Antirrhinum majus*) [60], silique dehiscence (*Arabidopsis*) [61], and fruit ripening (*Vitis vinifera*, pears, and tomatoes) [62–64]. The *ScAG* orthologous gene in tomato, *TAGL1*, showed the dual function of the AGAMOUS family in pigmentation regulation and organ identity [65, 66]. However, overexpressing AGAMOUS-like family genes from some species did not result in homeotic transformation but caused a colour change in transgenic plants. In petunia, the AGAMOUS-like family developed two lineage genes, *PLE* and *euAG*, and overexpressing the *PLE* lineage gene *FBP6* produced only smaller petals, but with no homeotic transformations of sepals and petals [67]. In *Prunus mume*, overexpressing the AGAMOUS homologous gene *PmAG* in transgenic tobacco caused the flower colour to fade with no phenotypic changes in the floral organs [68], a very similar observation to this study’s results. *ScAG* and *ScAGL11* probably lose their original functions as C-class genes related to organ identity and gain novel functions in the new biological process.

In summary, two MADS-box genes *ScAG* and *ScAGL11*, belonging to the AGAMOUS subfamily, were identified as inhibitors of anthocyanin accumulation and independently or cooperatively regulated anthocyanin biosynthesis. *ScAG* and *ScAGL11* separately inhibited the expression of ABP structural genes *ScF3H1* and *ScDFR3* by directly binding to their promoters, which influenced anthocyanin accumulation and bicolour pattern formation.

## Materials and methods

### Plant materials and growth conditions

Bicolour and pure colour cultivars of cineraria ‘Jester’, including carmine bicolour, pink bicolour, pure white, pure carmine, pure pink, and pure blue cultivars (named JeCB, JePB, JeW, JeC, JeP and JeB, respectively, in this study; Fig. 1a), were included in this study. Ray florets of the above cultivars were collected at five developmental stages: Stage 1 (S1) flower bud stage; Stage 2 (S2) ray floret stretching stage; Stage 3 (S3) ray floret stretching complete stage; Stage 4 (S4) initiating blooming stage; and Stage 5 (S5) blooming stage. The collected materials were immediately frozen in liquid nitrogen and stored at −80°C for further analysis. Ray florets of JeCB and JePB at S5 were used for HPLC–MS analysis. Ray florets of JePB at S2 (with removed ovaries) were used to build the transcriptome database. For gene expression analysis, ray florets (without ovaries) of JeCB, JePB, JeW, JeC, JeP, and JeB at S1–S5 were used. However, the VIGS experiments used JeP seedlings at the two-leaf stage and JeCB capitulum at S1 or S2. *N. tabacum* ‘NC89’ was used for the transgenic tobacco experiment and *N*. *benthamiana* for subcellular localization and BiFC and LUC reporter assays. All cineraria plants were grown under 8 hours day/16 hours night, and tobacco was cultivated under 16 hours day/8 hours night at 22°C in an artificial climate chamber.

### Light microscopy and scanning electron microscope analysis for morphological observation of ray florets

Fresh tissues of JeW, JeC, JeP, and JeB and coloured and colourless regions of JeCB and JePB ray florets at S5 were used to prepare plant slices. The upper and lower epidermises of ray florets were manually cut into small squares (~10 mm × 5 mm) using shaver blades and forceps and placed on a slide for observation with an Olympus light microscope (Tokyo, Japan). Photographs were taken using an attached camera (Tokyo, Japan).

The SEM was used to determine the 3D intracellular structures of JeCB ray florets. Essentially, fresh ray florets were divided into three sections, including the colourless, coloured, and central (the area between the colourless and coloured areas) regions. Tissues (each 5 mm × 5 mm) were fixed in 2% glutaraldehyde under a vacuum environment at 0.8 kg/cm^2^ for 6–8 hours. The tissues were dehydrated once in a graded ethanol series of 30, 50, 70, 90, and 95%, and twice in 100% ethanol, each for 10 min. Then, tissues were further treated with a graded *tert*-butyl alcohol series of 30, 50, 70, and 100%, each for 5 min, and dried in a FreeZone 4.5-L vacuum freeze dryer (Labconco, MO, USA) for 4 hours. The dried specimens were sputter-coated (Hitachi E1010, Tokyo, Japan) with platinum and examined using an SEM (Hitachi SU8010, Tokyo, Japan) at 10 kV accelerating voltage under high vacuum.

### HPLC–MS analysis

At S5, tissues from the coloured and colourless regions of JeCB and JePB ray florets were sampled for HPLC–MS anthocyanin component and content analysis as previously described [1]. Briefly, 250 mg of the petal was ground in liquid nitrogen, dipped in extract solution (methanol:ultrapure water:formic acid:trifluoroacetate = 70:27:2:1, v/v/v/v) for 24 hours at 4°C, and centrifuged at 12 000 × g for 5 min. The supernatant was filtered through a 0.22-μm membrane. An Agilent-1100 HPLC/MS Trap VL system (Agilent Technologies, CA, USA) was used for anthocyanin quantification. A reversed-phase C18 column (ODS-80Ts QA, Tosoh Corporation, Tokyo, Japan) connected to a C18 guard column (Shanghai ANPEL Scientific Instrument, Shanghai, China) was used as the chromatographic column. A linear gradient of %A (ultrapure water:methane acid:trifluoroacetic acid = 97.9:2:0.1, v/v/v) in %B (acetonitrile:methane acid:trifluoroacetic acid = 62.9:35:2:0.1, v/v/v/v) generated the chromatogram results, which were read at 530 nm for anthocyanins. The standard was cyanin chloride (Sigma, MO, USA). Finally, the pigment component mass spectrographic results were compared with those for pure colour cineraria from a previous study [1].

### Transcriptome sequencing and analysis

The samples of coloured and colourless regions of JePB ray florets at S2 were used to construct six libraries with three biological replicates for transcriptome analysis as described previously [1, 69]. For each sample, ray florets were collected at the same time from three independent JePB plants from batches that budded at the same time. Briefly, total RNA was extracted using the Plant RNA Rapid Extraction Kit (HUAYUEYANG Biotechnology, Beijing, China), and the quality was evaluated with a NanoDrop 2000 (Thermo Fisher Scientific, MA, USA). The mRNA was enriched using oligo(dT) magnetic beads, and short fragments were obtained by adding fragmentation buffer to mRNA, and these were used as templates for the first strand of cDNA syntheses. The subsequent steps included second-strand cDNA synthesis using the two-strand synthesis system, purification of short fragments using the QiaQuick PCR extraction kit (Qiagen, Hilden, Germany), end repair, and addition of poly(A). The short fragments were then connected to sequencing adapters, and suitable-sized fragments were selected for PCR amplification library construction. The cDNA was evaluated for quality and sequenced using an Illumina HiSeq™ 2000 (Illumina, CA, USA).

Low-quality sequences were removed from raw reads, and the remaining clean reads were assembled using SOAPdenovo software with the parameters set to –K29, −M2, and –L50. First, the sequences were assembled into contigs without gaps, then the gaps between different scaffolds were filled using double-end sequencing to obtain unigenes. The unigenes were annotated using databases including NR, TmHMM, SignalP, Prot, RNAMMER, BLASTP, Map, BLASTX, eggNOG, Map, PFAM, and BLASTX. Then, we calculated the fragments per kilobase of transcript per million mapped (FPKM) reads for each unigene for transcript abundance analysis. The Benjamini–Hochberg method was used to correct the *P*-value [70]. Finally, the DEGs were identified using the threshold for the false discovery rate (FDR) significance score, *P* <.05, and |Log_2_FoldChange| ≥ 1. The key DEGs (genes whose expression levels differed at least 10-fold between colourless and coloured regions) were selected from DEGs using the threshold for |Log_2_FoldChange| ≥ log_2_10. Furthermore, a qRT–PCR analysis validated the transcriptome data by detecting the expression of seven DEGs (TRINITY_DN11487_c0_g1, TRINITY_DN15133_c1_g1, TRINITY_DN17756_c4_g5, TRINITY_DN19492_c0_g2, TRINITY_DN9621_c1_g1, TRINITY_DN18674_c1_g1, and TRINITY_DN20125_c0_g1) associated with the ABP in JePB at S2. qRT–PCR was performed with a SYBR Premix Ex Taq kit (Takara, Kusatsu-Shiga, Japan) with three replicates on a Mini Opticon Real-time PCR System (Bio-Rad, CA, USA). All primers used in this study are listed in Supplementary Data Table S11.

### Gene expression analysis

Total RNA was extracted from ray florets of JeCB, JePB, JeW, JeC, JeP, and JeB at S1–S5 using the Plant RNA Extraction Kit (HUAYUEYANG Biotechnology, Beijing, China). The cDNA was synthesized using a Reverse Transcription System (Promega, WI, USA). The RT–PCR and qRT–PCR expression of the DEGs was conducted as described previously [1]. RT–PCR was used to detect the expression of the DEGs in the ray florets of JeCB, JePB, JeW, JeC, JeP, and JeB at S1–S2. However, the qRT–PCR analysis determined DEG expression in the coloured and colourless regions of the JeCB and JePB ray florets at S1–S5. All experiments were conducted with three biological replicates, and all primers used in this study are listed in Supplementary Data Table S11.

### Hereditary and expression analysis of candidate genes in the hybrid (*F*_1_) population

JeCB and JeW cultivars were used as female and male parents to analyse the hereditary characteristics of the bicolour pattern. The stamens were removed before spreading pollen to avoid selfing, and the ray florets of JeCB were cut short, exposing stigmas. The capitulum was bagged after pollination. One month later, the *F*_1_ seeds were harvested and germinated in the substrate (turf:vermiculite = 1:1) and later placed under 8 hours day/16 hours night at 22°C in an artificial climate chamber. The *F*_1_ flower phenotypes were documented for analysis of the inheritance of the cineraria bicolour trait. The S2 capitulums of 10 *F*_1_ plants, including 8 carmine bicolour and 2 pure carmine progenies, were sampled to investigate the candidate genes co-expressed with the bicolour phenotype. Colourless and coloured regions of ray florets from bicolour plants were sampled separately, while ray florets from pure colour plants were sampled as a whole. All materials were immediately frozen in liquid nitrogen and stored at −80°C. The expression levels of *ScAG* and *ScAGL11* genes in the above samples were determined using RT–PCR. All the primers used in this study are listed in Supplementary Data Table S11.

### Full-length cDNA isolation and phylogenetic analysis

The 5′-terminal fragment gene sequences were obtained from transcriptome data, and the 3′-terminal cDNA sequences were determined using rapid amplification of cDNA ends–PCR (RACE–PCR). The full-length sequences were cloned using the cDNAs from coloured and colourless regions of JeCB ray florets at S2 [71], and the *ScAG* and *ScAGL11* sequences were deposited in GenBank (accession numbers OL598710 and OL598711, respectively). The amino acid sequences were translated from the above nucleotides using MEGA 7.0 software [72], and used for BLAST analysis on the NCBI database. Multiple sequence alignment was conducted using MUSCLE and Boxshade (https://sourceforge.net/projects/boxshade/) [73]. The following sequences with high homology to *ScAG* or *ScAGL11* were retrieved from GenBank: AAO22984.1 (*C. × morifolium*); CAA08800.1 (*G. hybrida*); XP_022038383.1 (*Helianthus annuus*); AAO18228.1 (*H. annuus*); XP_023732805.1 (*L. sativa*); XP_019176800.1 (*Ipomoea nil*); XP_023760420.1 (*L. sativa*); XP_009770275.1 (*Nicotiana sylvestris*); XP_009599025.1 (*N. tomentosiformis*); XP_011096026.1 (*Sesamum indicum*). All *Arabidopsis* MIKC-type MADS-box TFs were downloaded from The Arabidopsis Information Resource (http://www.arabidopsis.org/index.jsp) to perform phylogenetic analysis (Supplementary Data Table S6). Additional genes related to MADS-box TFs derived from other species were obtained from the NCBI database (Supplementary Data Table S6). Next, neighbour-joining phylogenetic trees were built using MEGA 7.0 [73], with p-distance amino acid substitutions, uniform rates among sites, and pairwise deletions to treat missing data. The clade support was estimated using 1000 bootstraps. All primers used in this study are listed in Supplementary Data Table S11.

### Subcellular localization

The ORFs of *ScAG* and *ScAGL11* without the stop codon were inserted into eGFP vectors driven by the *CaMV35S* promoter with XhoI and SalI restriction sites (Supplementary Data Fig. S11a). However, recombinant vectors were transformed into *Agrobacterium* strain GV3101-psoup and injected into *N. benthamiana* leaves following the Hellens protocol [74]*.* Two days after injection, the GFP fluorescence signal was observed using a Leica SP8 microscope (Leica Microsystems, Wetzlar, Germany). Each experiment was repeated three times, and all primers used in this study are listed in Supplementary Data Table S11.

### Tobacco transformation

For gene functional analysis, the ORFs of *ScAG* and *ScAGL11* were inserted into pBI121 vectors driven by the *CaMV35S* promoter with XbaI and BamHI restriction sites (Supplementary Data Fig. S11b). The recombinant vectors were transferred into *Agrobacterium* strain GV3101. *N. tabacum* cultivar ‘NC89’ leaf discs were used as explants for transgenic transformation as described by Horsch and Klee [75]. Next, plantlets were transferred into soil and cultivated in an artificial climate chamber. All transgenic plants were confirmed by PCR amplification using a specific upstream primer from the *CaMV35S* promoter and specific downstream primers for *ScAG* and *ScAGL11*. Wild-type tobacco plants and transgenic lines were cultivated under 16 hours day/8 hours night at 22°C in an artificial climate chamber. The flower conditions were recorded and photographed after blossoming. Thereafter, the expression of ABP structural genes [*NtCHS* (KU949017.1), *NtCHI* (NM_001325287.1), *NtF3H* (NW_015891008.1), *NtF3′H* (NW_015898127.1), *NtDFR* (NW_015927898.1), and *NtANS* (NW_015903696.1)] and regulatory genes [*NtAN1a* (NW_015891756.1) and *NtAN1b* (NW_015920078.1)] in transgenic plants with phenotypic changes was analysed by qRT–PCR. All primers used in this study are listed in Supplementary Data Table S11.

### Virus-induced gene silencing in leaves of cineraria ‘Jester’ pink

Here, pTRV1 (pYL192) and pTRV2 (pYY13) vectors were used to conduct a VIGS experiment in capitulum to characterize the functions of *ScAG* and *ScAGL11*. *ScAG* and *ScAGL11* fragments measuring 178 and 179 bp with LIC1 (CGACGACAAGACCCT) and LIC2 (GAGGAGAAGAGCCCT) in the 5′-termini of upstream and downstream primers were inserted into the pTRV2 (Tobacco rattle virus) vectors using the PstI restriction site [35] (Supplementary Data Fig. S11c)_._ The PstI (New England Biolabs, MA, USA) enzyme digested the pTRV2 vector. Then, linear pTRV2 vectors and target fragments were treated with T_4_ DNA polymerase and ligated using T_4_ DNA ligase (New England Biolabs, MA, USA) to generate pTRV2-*ScAG* and pTRV2-*ScAGL11*, respectively. Recombinant vectors were transformed into *Agrobacterium* strain GV3101 by the freeze–thaw method [76].

The *Agrobacterium* mixture containing pTRV1, pTRV2-*ScAG*, and pTRV2-*ScAGL11* was cultured at 28°C for 16 hours, resuspended to OD_600_ = 1.5 in an infiltration buffer containing 10 mM MgCl_2_, 200 mM acetosyringone, and 10 mM MES at pH 5.6, and incubated at room temperature for 3 hours. A previously built and optimized VIGS system [34] performed the infiltration. *Agrobacterium* GV3101 cells containing the pTRV1 and pTRV2 vectors were mixed at a 1:1 ratio and injected into the abaxial leaf surface of JeP seedlings at the two-leaf stage. The mixture had four combinations: pTRV1 mixed with empty pTRV2 (CK); pTRV1 mixed with pTRV2-*ScAG*; pTRV1 mixed with pTRV2-*ScAGL11*; and pTRV1 mixed with pTRV2-*ScAG* and pTRV2-*ScAGL11.* After infiltration, the inoculated plants were grown in an artificial climate chamber at 10°C for 2 days and at 15°C for 1 day in the dark. The plants were then cultivated at 20°C under 16 hours of light in 60% relative humidity. Afterwards, regions with phenotypic changes were verified by RT–PCR using specific primers to check for gene silencing. The anthocyanin content of the coloured regions of silenced leaves was detected as described in the section HPLC–MS analysis. The qRT–PCR technique determined the expression of *ScCHS2*, *ScF3H1*, *ScDFR3*, *ScANS*, *ScbHLH17*, *ScAG*, and *ScAGL11* genes in the silenced leaves. All data were detected in at least three silenced lines, and all primers used in this study are listed in Supplementary Data Table S11.

### Virus-induced gene silencing in cineraria capitulum

*Agrobacterium* strain GV3101 cells containing pTRV1 and pTRV2 vectors were mixed at a 1:1 ratio as described for leaf infiltration (except that the OD_600_ of the mixture was changed to 1.0) [34] to silence capitulum *ScAG* and *ScAGL11*. The mixture was introduced by needle injection into the buds of JeCB at S1 from the scapes. The mixture contained four combinations: pTRV1 mixed with empty pTRV2 (CK); pTRV1 mixed with pTRV2-*ScAG*; pTRV1 mixed with pTRV2-*ScAGL11*; and pTRV1 mixed with pTRV2-*ScAG* and pTRV2-*ScAGL11.* Moreover, the whole JeCB capitulum was submerged in an infiltration mixture to conduct vacuum infiltration at 0.8 kg/cm^2^ for 10 minutes twice, as described previously [34]. The growth conditions of the inoculated plants were as described for leaf infiltration. In the silenced capitulum, the expression of *ScCHS2*, *ScF3H1*, *ScDFR3*, *ScANS*, *ScAG*, and *ScAGL11* genes was analysed via qRT–PCR. The data were obtained in at least three silenced lines.

### Yeast two-hybrid assays

The Matchmaker™ Gold Yeast Two-hybrid System (Clontech Laboratories, CA, USA) was used to perform Y2H assays following the manufacturer’s instructions to explore the relationship between *ScAG* and *ScAGL11*. The *ScAG* and *ScAGL11* ORFs were inserted into pGADT7 and pGBKT7 vectors to obtain AD-*ScAG*, AD-*ScAGL11*, BD-*ScAG*, and BD-*ScAGL11* recombinant vectors (Supplementary Data Fig. S11d), and the recombinant vectors were co-transformed into competent yeast cells (strain Y2H Gold). After verifying no self-activation activity, the recombinant vectors were spread on SD/−Trp−Leu medium. Finally, a single colony was picked and diluted 1-, 10-, and 100-fold to inoculate the SD/−Trp/−Leu/−His/−Ade screening medium. All the primers used in this study are listed in Supplementary Data Table S11.

### Bimolecular fluorescence complementation assay

The *ScAG* and *ScAGL11* ORFs without the stop codon were separately inserted into the N-terminal or C-terminal regions of YFP using XbaI and BamHI restriction sites to construct *35S*::*ScAG*-zYNE and *35S*::Sc*AGL11*-zYCE vectors (Supplementary Data Fig. S11e). The recombinant vectors were transferred into *Agrobacterium* strain GV3101-psoup-p19 and transiently co-transformed into *N. benthamiana* leaves. The *N. benthamiana* transient expression method was described by Hellens *et al*. [74]. *35S*::*ScAG*-zYNE co-transformed with *35S*::*ScAGL11*-zYCE and *35S*::zYCE co-transformed with *35S*::zYNE were the negative controls. The YFP fluorescence signal was imaged 48 hours after injection using a Leica SP8 microscope (Leica microsystems, Wetzlar, Germany). Each experiment was repeated three times. All the primers used in this study are listed in Supplementary Data Table S11.

### Isolation of promoter sequences using genome walking

The promoter sequences of *ScCHS2*, *ScDFR3*, and *ScANS* were cloned from genomic DNA of JeCB ray florets using the GenomeWalker kit (Takara, Kusatsu-Shiga, Japan). The specific primers were designed based on the transcriptome cDNA sequences listed in Supplementary Data Table S11. The prediction and analysis of *cis*-elements in promoters were performed using PlanCARE (http://bioinformatics.psb.ugent.be/webtools/plantcare/html).

### Yeast one-hybrid assay

The Matchmaker™ Gold Yeast One-hybrid System (Clontech Laboratories, CA, USA) was used for the Y1H assay to examine the combinations of *ScAG*/*ScAGL11* and *ScCHS2*, *ScDFR3*, and *ScANS* promoters. First, promoter sequences were shortened into several 200- to 400-bp fragments containing the CArG box to avoid irrepressible self-activation activity of bait vectors. The shortened fragments were inserted into the pAbAi vector using SalI and SacI restriction sites, to produce the following recombination vectors: pAbAi-*ScCHS2*-pro-1/2 (containing CArG box 1 and 2 in *ScCHS2* promoter); pAbAi-*ScF3H1*-pro-1 (containing CArG box 1 in *ScF3H1* promoter); pAbAi-*ScF3H1*-pro-2 (containing CArG box 2 in *ScF3H1* promoter); pAbAi-*ScF3H1*-pro-3 (containing CArG box 3 in *ScF3H1* promoter); pAbAi-*ScDFR3*-pro-1 (containing CArG box 1 in *ScDFR3* promoter); and pAbAi-*ScDFR3*-pro-2 (containing CArG box 2 in *ScDFR3* promoter) (Supplementary Data Fig. S11f). Then, the pAbAi recombination vector was linearized and linked to the Y1H Gold strain, and these yeast cells were examined on SD/−Leu medium with Aureobasidin A (AbA) to inhibit self-activation activity. The AD-*ScAG*/AD-*ScAGL11* vectors were transformed into yeast cells containing pAbAi recombination vectors and spread on SD/−Leu medium. Next, a single colony was picked and diluted 10-fold to inoculate the SD/−Leu medium with an optimal AbA concentration. The empty vector (AD) served as a negative control, and each experiment was repeated three times. All the primers used in this study are listed in Supplementary Data Table S11.

### LUC reporter assay

The *ScAG* and *ScAGL11* ORFs were cloned into pGreenII62-SK vectors (effectors) to investigate the regulatory relationship between two MADS-box genes and the key ABP structure genes. Consequently, *ScF3H1* and *ScDFR2* promoter sequences were cloned into pGreenII62-LUC vectors (reporters) (Supplementary Data Fig. S11g). Next, the *ScAG*-SK, *ScAGL11*-SK, *ScF3H1*-LUC, and *ScDFR3*-LUC vectors were obtained and transformed into *Agrobacterium* strain GV3101-psoup. The *Agrobacterium* strains containing SK and LUC recombination vector were co-transformed into *N. benthamiana* leaves, and the transient expression followed the method of Hellens *et al*. [74]. The empty vectors (SK) co-transformed with LUC recombination vectors were used as a negative control. The activity of firefly luciferase (LUC) and *Renilla* luciferase (REN) was detected 72 h after injection using Dual Luciferase Reporter Assay System reagents (Promega, WI, USA). The ratio of LUC to REN determined the activity, and each experiment was repeated three times. All the primers used in this study are listed in Supplementary Data Table S11.

## Supplementary Material

Web_Material_uhac071

## Data Availability

All data supporting the findings of this study are available within the paper and within the supplementary materials published online.
