# Supplementary material for: Functional analysis of the ScAG and ScAGL11 MADS-box transcription factors for anthocyanin biosynthesis and bicolour pattern formation in Senecio cruentus ray florets
Source: Hortic Res. 2022 Mar 23;9:uhac071. doi: 10.1093/hr/uhac071 (PMC9209810; doi:10.1093/hr/uhac071)
Supplement: Web_Material_uhac071 [file web_material_uhac071.zip › Hortres-04868R3-Figure1-7 and FigureS1-S11.pptx]

## Slide 1
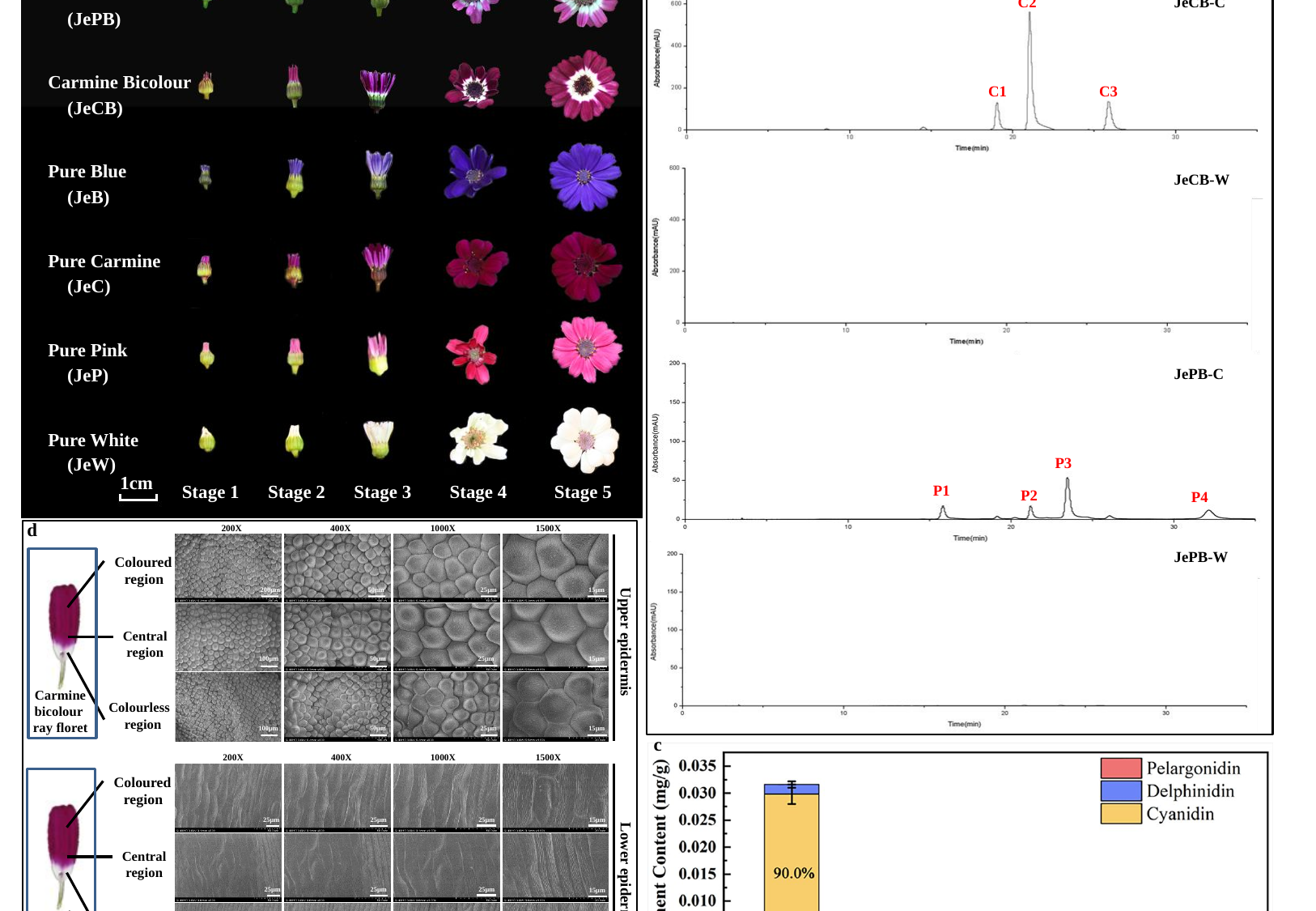

a
d
c
b
C2
JeCB-C
C1
C3
JeCB-W
JePB-C
P3
P1
P2
P4
JePB-W
Pink Bicolour
(JePB)
Carmine Bicolour
(JeCB)
Pure Blue
(JeB)
Pure Carmine
(JeC)
Pure Pink
(JeP)
Pure White
(JeW)
1cm
Stage 1
Stage 2
Stage 3
Stage 4
Stage 5
1500X
200X
400X
1000X
25μm
200μm
50μm
15μm
 Coloured
region
50μm
15μm
100μm
25μm
Central
region
Upper epidermis
50μm
15μm
25μm
Carmine bicolour
ray floret
Colourless
region
100μm
1500X
200X
400X
1000X
25μm
25μm
25μm
15μm
25μm
25μm
25μm
15μm
Lower epidermis
25μm
25μm
25μm
15μm
 Coloured
region
Central
region
Carmine bicolour
ray floret
Colourless
region
Figure 1a-1d

## Slide 2
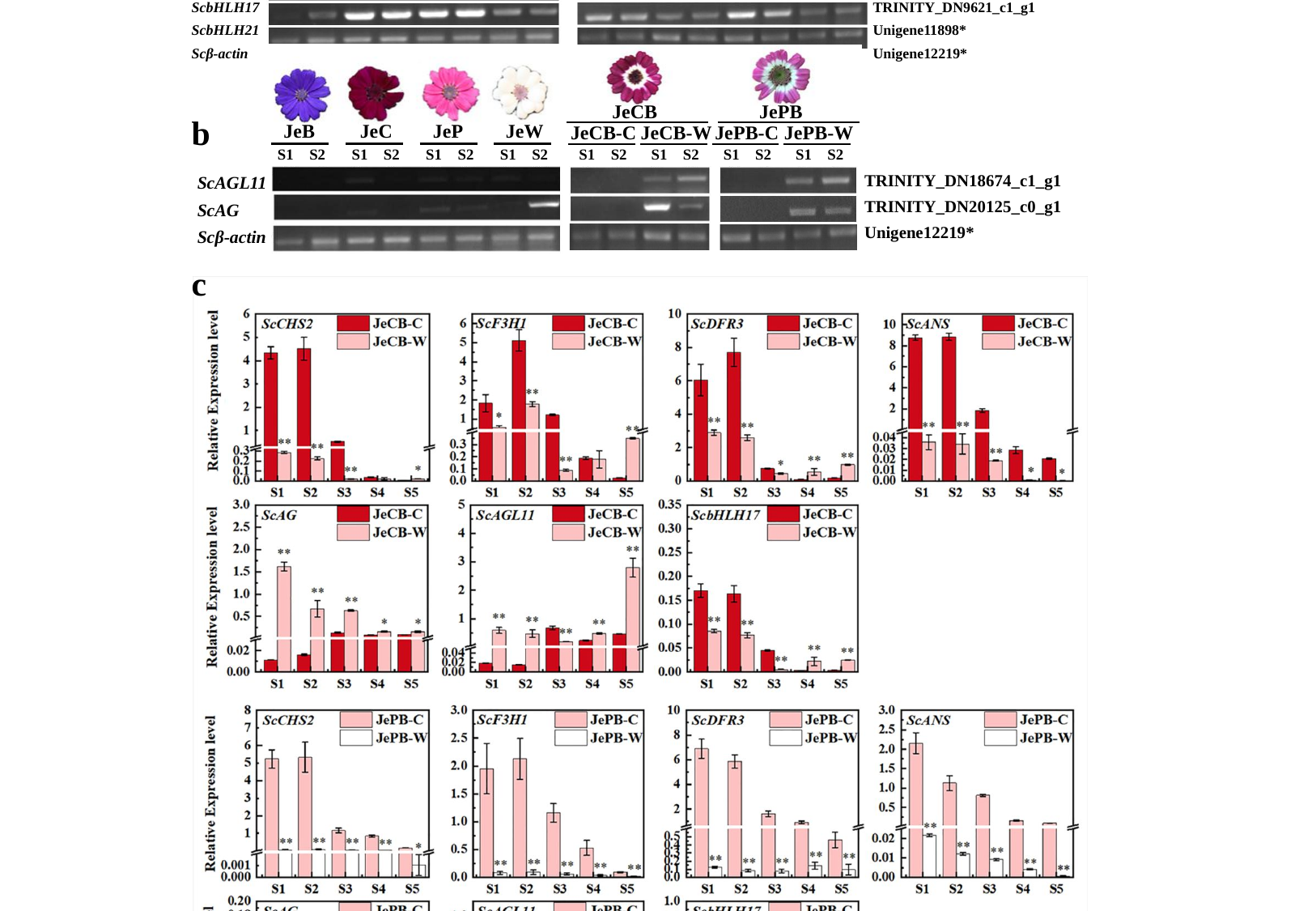

JeCB
JePB
JeCB-C
JeCB-W
JePB-C
JePB-W
S1
S2
S1
S2
S1
S2
S1
S2
JeB
JeC
JeP
JeW
S1
S2
S1
S2
S1
S2
S1
S2
a
b
c
TRINITY_DN11487_c0_g1
Unigene12332*
Unigene12439*
TRINITY_DN15133_c1_g1
Unigene17284*
CL6923.Contig1*
CL11609.Contig2*
Unigene2908*
CL4194-2*
TRINITY_DN17756_c4_g5
CL6582.Contig1*
TRINITY_DN19492_c0_g2
CL4631.Contig4*
CL4406*
CL5332*
CL5801*
CL14095*
Unigene3728*
CL13611*
Unigene9051*
Unigene6724*
CL107*
CL3474*
TRINITY_DN9621_c1_g1
Unigene11898*
Unigene12219*
TRINITY_DN18674_c1_g1
TRINITY_DN20125_c0_g1
Unigene12219*
ScCHS2
ScCHS3
ScCHI1
ScF3H1
ScF3H5
ScF3H6
ScF3H7
ScF3'H1
ScF3'5'H
ScDFR3
ScDFR5
ScANS
ScGST3
ScMYB7
ScMYB8
ScMYB10
ScMYB22
ScMYB23
ScMYB28
ScMYB31
ScMYB48
ScbHLH1
ScbHLH6
ScbHLH17
ScbHLH21
Scβ-actin
JeCB
JePB
JeCB-C
JeCB-W
JePB-C
JePB-W
S1
S2
S1
S2
S1
S2
S1
S2
JeB
JeC
JeP
JeW
S1
S2
S1
S2
S1
S2
S1
S2
ScAGL11
ScAG
Scβ-actin
Figure 2a-2c

## Slide 3
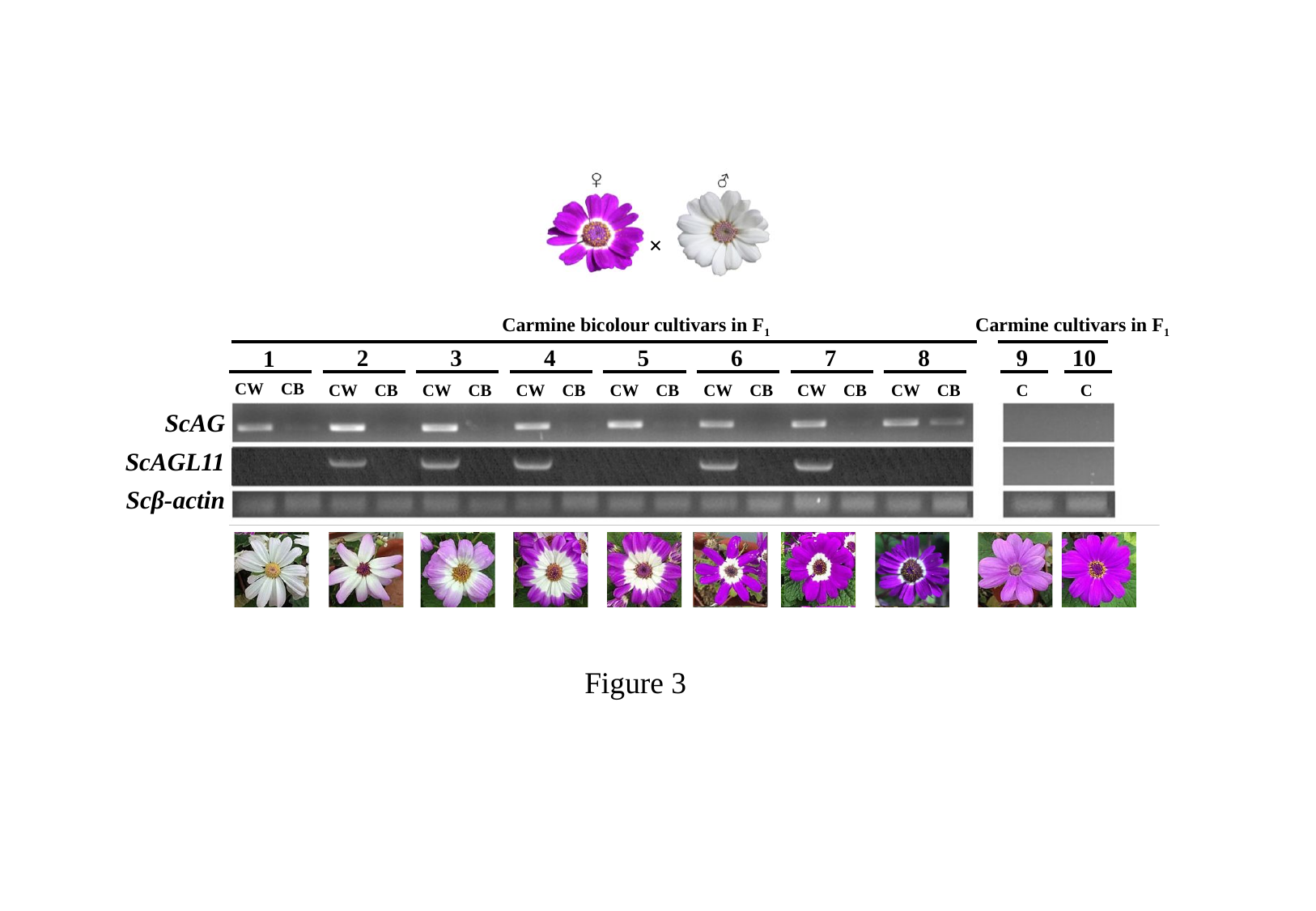

×
b
Carmine bicolour cultivars in F1
2
3
4
5
6
7
8
1
CW
CB
CW
CB
CW
CB
CW
CB
CW
CB
CW
CB
CW
CB
CW
CB
Carmine cultivars in F1
9
10
C
C
ScAG
ScAGL11
Scβ-actin
Figure 3

## Slide 4
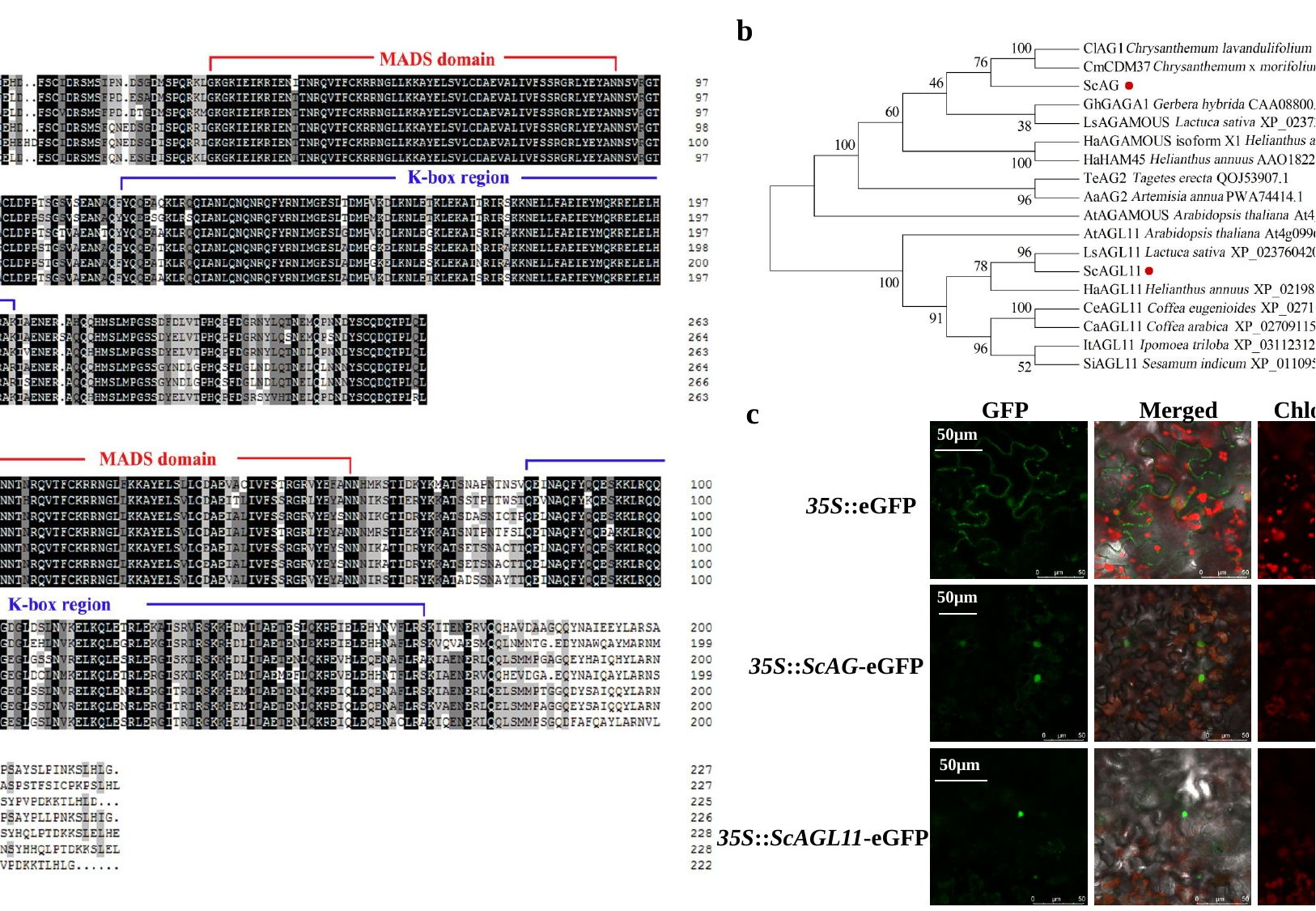

b
a
b
c
GFP
Merged
ChlorophyII
Bright
35S::eGFP
35S::ScAG-eGFP
35S::ScAGL11-eGFP
50μm
50μm
50μm
Figure 4a-4c

## Slide 5
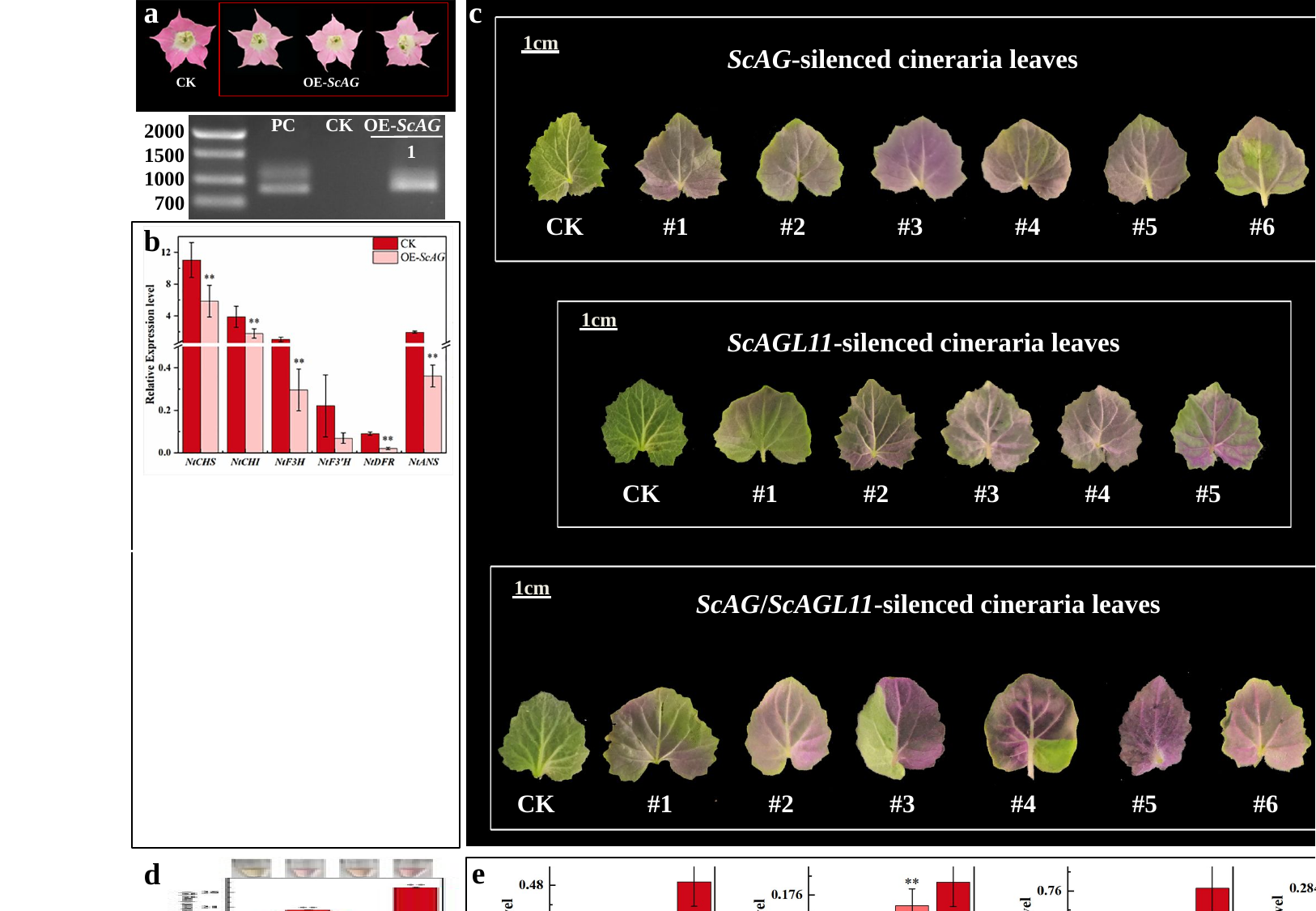

c
1cm
1cm
d
e
d
e
a
c
CK
OE-ScAG
b
1cm
ScAG-silenced cineraria leaves
1cm
ScAGL11-silenced cineraria leaves
1cm
ScAG/ScAGL11-silenced cineraria leaves
d
CK
#1
#2
#3
#4
#5
#6
CK
#1
#2
#3
#4
#5
CK
#1
#2
#3
#4
#5
#6
PC
CK
OE-ScAG
2000
1500
1000
700
1
b
Figure 5a-5e

## Slide 6
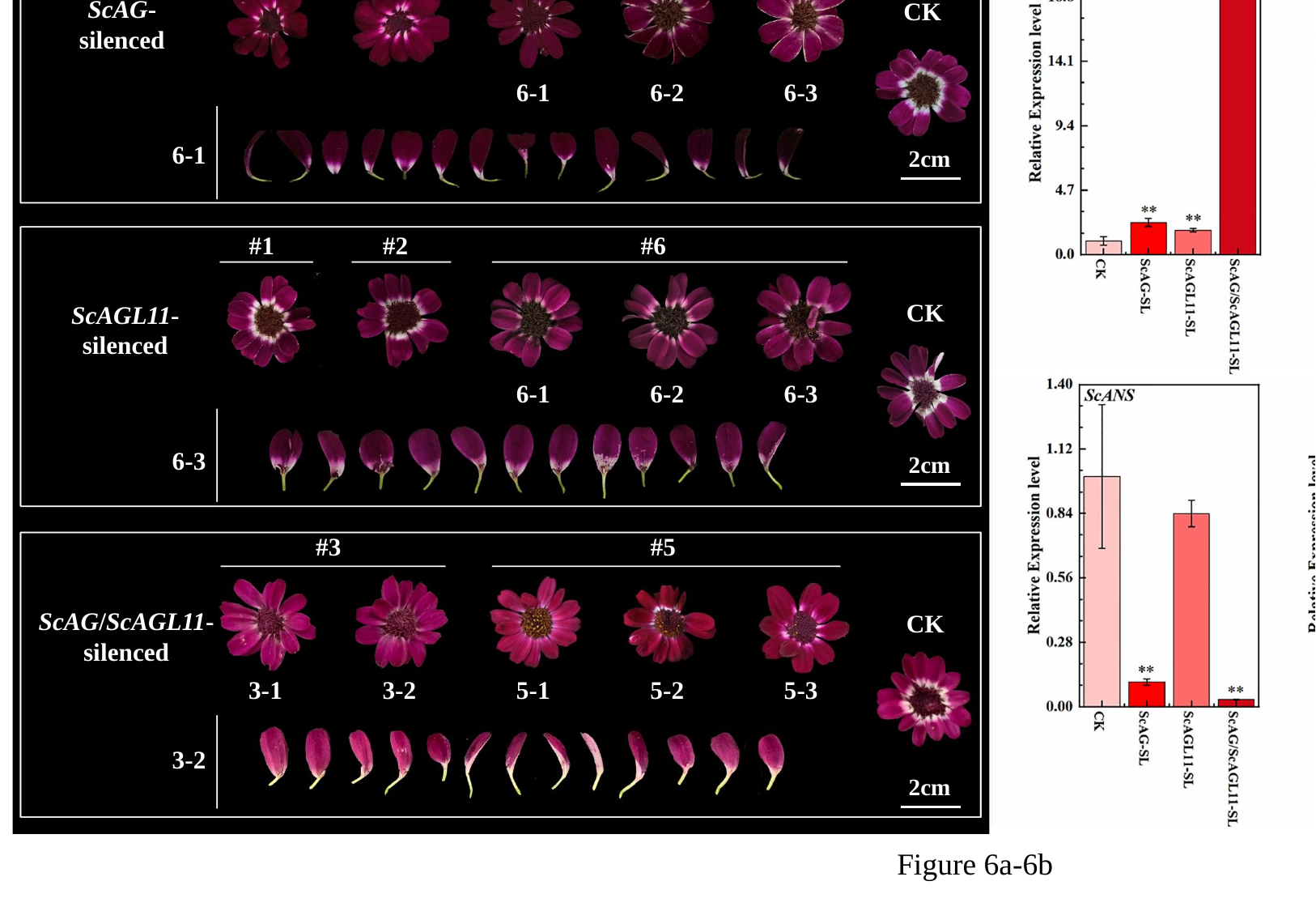

a
#1
#5
#6
ScAG-silenced
CK
6-1
6-2
6-3
6-1
2cm
#1
#2
#6
CK
ScAGL11-silenced
6-1
6-2
6-3
6-3
2cm
#3
#5
ScAG/ScAGL11-silenced
CK
3-1
3-2
5-1
5-2
5-3
3-2
2cm
2cm
2cm
2cm
b
Figure 6a-6b

## Slide 7
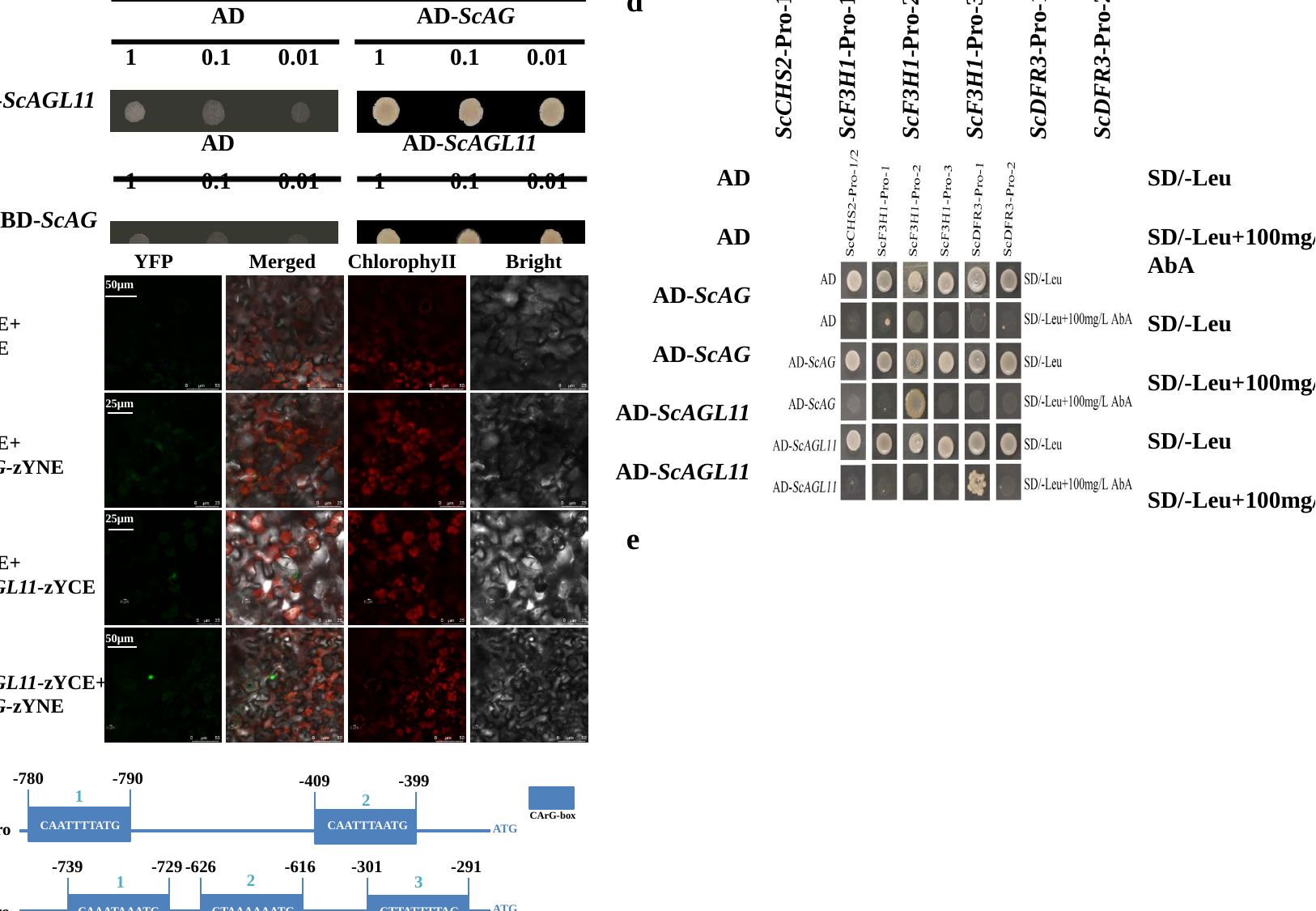

ScCHS2-Pro-1/2
ScF3H1-Pro-1
ScF3H1-Pro-2
ScF3H1-Pro-3
ScDFR3-Pro-1
ScDFR3-Pro-2
AD
AD
AD-ScAG
AD-ScAG
AD-ScAGL11
AD-ScAGL11
SD/-Leu
SD/-Leu+100mg/L AbA
SD/-Leu
SD/-Leu+100mg/L AbA
SD/-Leu
SD/-Leu+100mg/L AbA
SD/-Trp/-Leu/-His/-Ade
AD
AD-ScAG
1 0.1 0.01
1 0.1 0.01
BD-ScAGL11
AD
AD-ScAGL11
1 0.1 0.01
1 0.1 0.01
BD-ScAG
a
d
b
e
c
50μm
25μm
25μm
50μm
YFP
Merged
ChlorophyII
Bright
35S::zYCE+
35S::zYNE
35S::zYCE+
35S::ScAG-zYNE
35S::zYNE+
35S::ScAGL11-zYCE
35S::ScAGL11-zYCE+
35S::ScAG-zYNE
50μm
25μm
25μm
50μm
-780
-790
CAATTTTATG
-409
-399
ScCHS2-Pro
ATG
-739
-729
CAAATAAATG
-626
-616
CTAAAAAATG
-301
CTTATTTTAG
-291
ScF3H1-Pro
ATG
-730
-720
CTTTTATAAG
-535
CAAATTTTTG
-525
ScDFR3-Pro
ATG
CArG-box
1
2
2
1
3
1
2
CAATTTAATG
Figure 7a-7e

## Slide 8
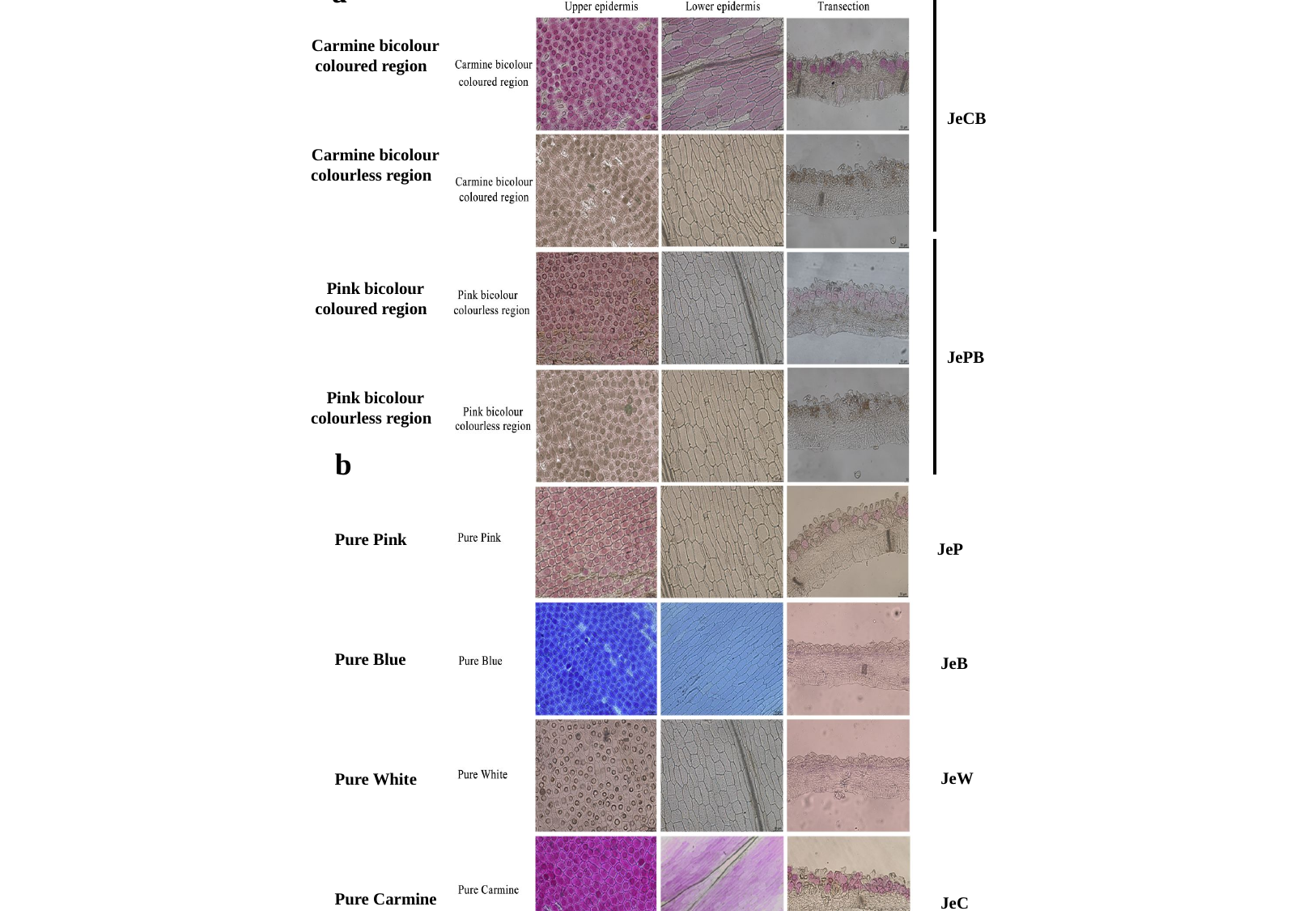

Upper epidermis
Lower epidermis
Transection
JeCB
JePB
JeP
JeB
JeW
JeC
a
b
Carmine bicolour
coloured region
Carmine bicolour
colourless region
Pink bicolour
coloured region
Pink bicolour
colourless region
Pure Pink
Pure Blue
Pure White
Pure Carmine
Figure S1a-1b

## Slide 9
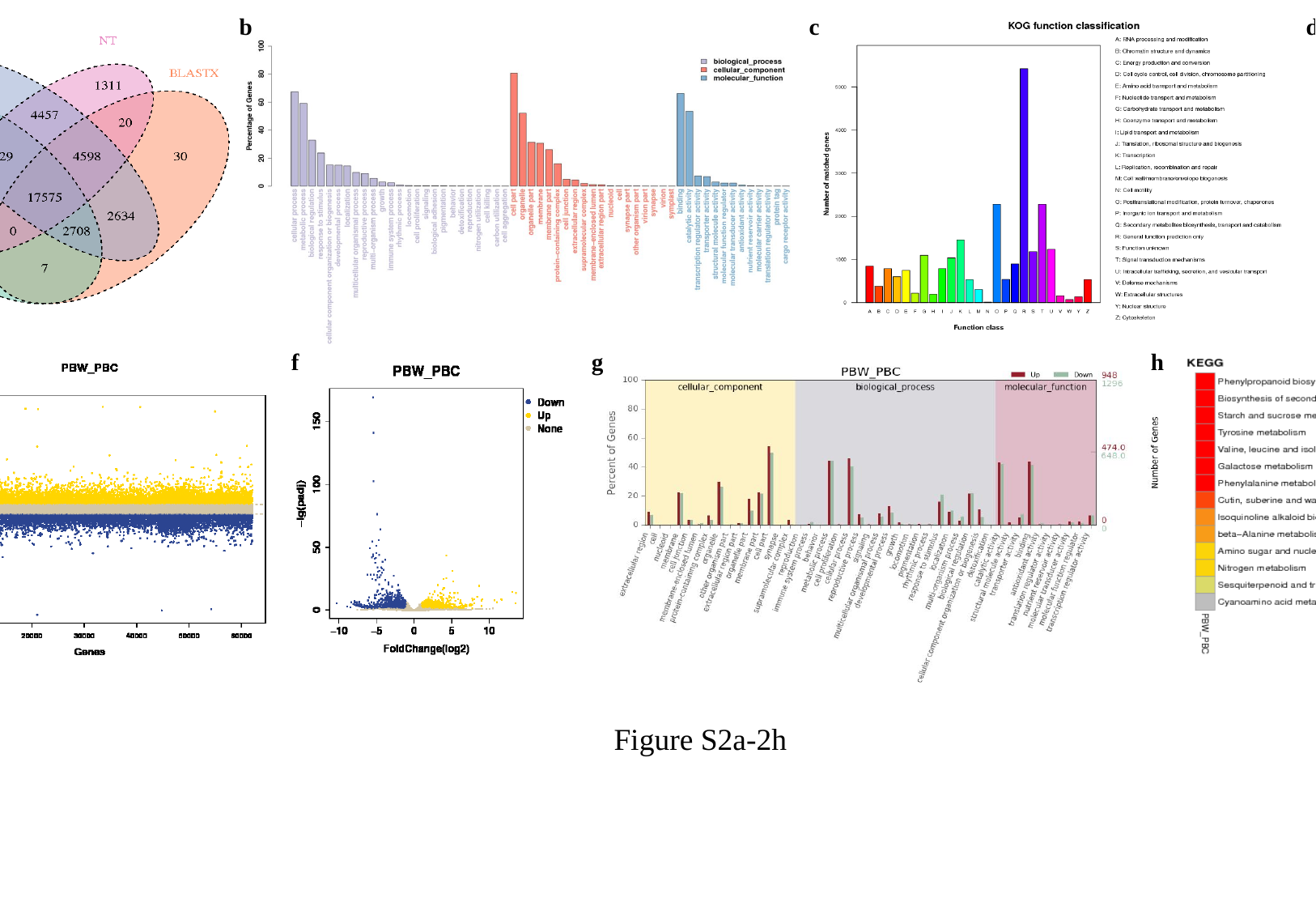

a
b
c
d
c
e
f
g
h
Figure S2a-2h

## Slide 10
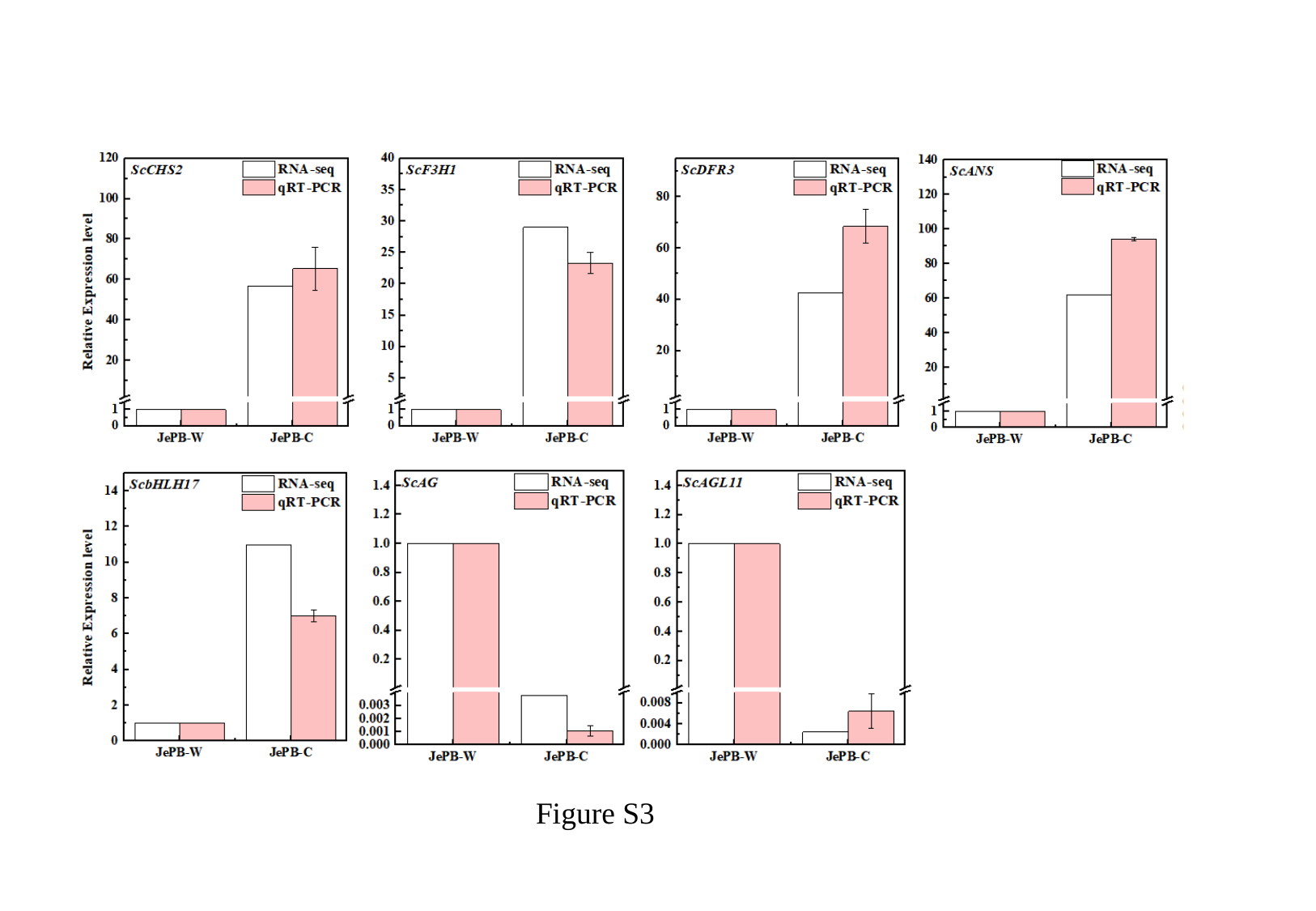

Figure S3

## Slide 11
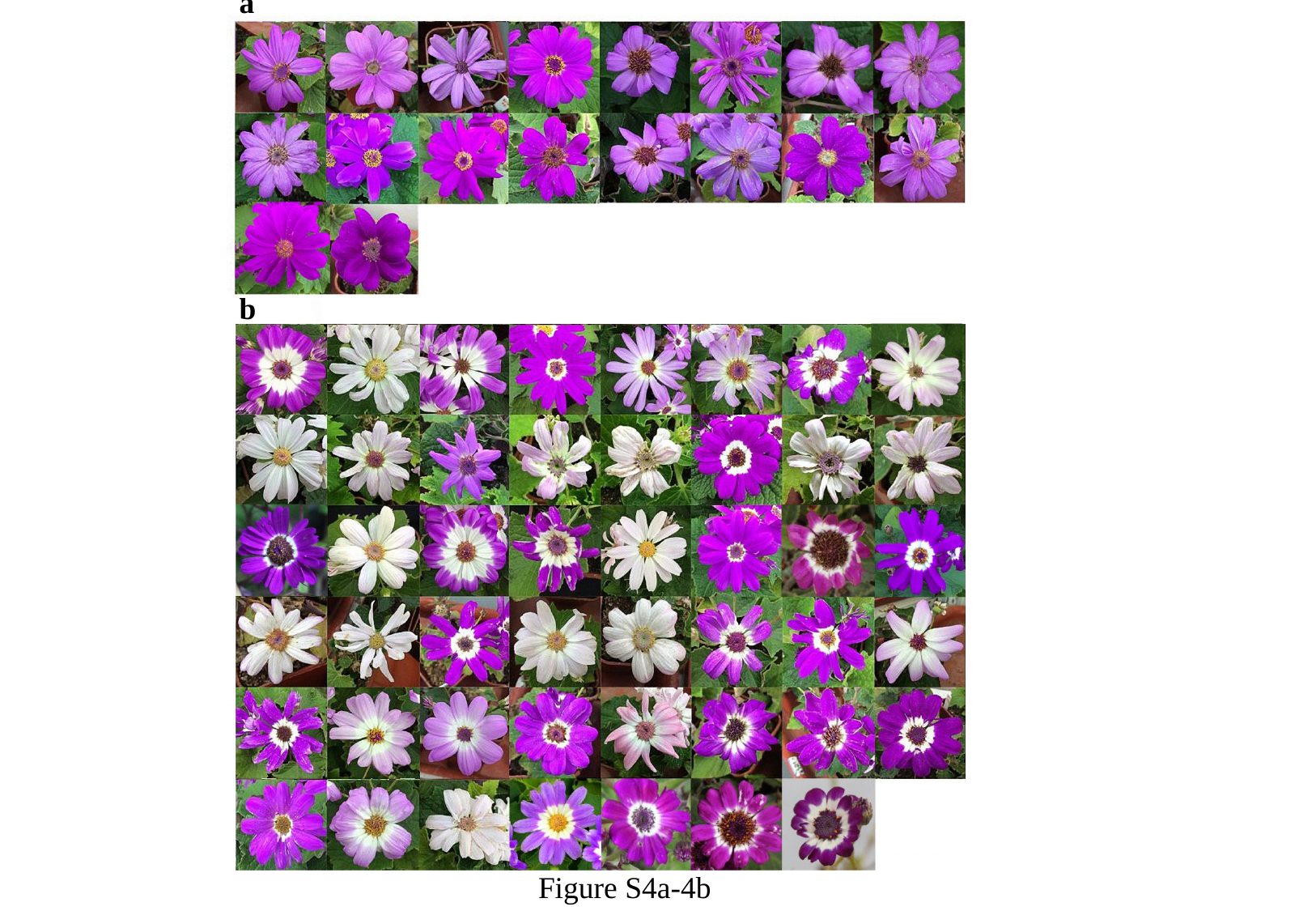

a
b
Figure S4a-4b

## Slide 12
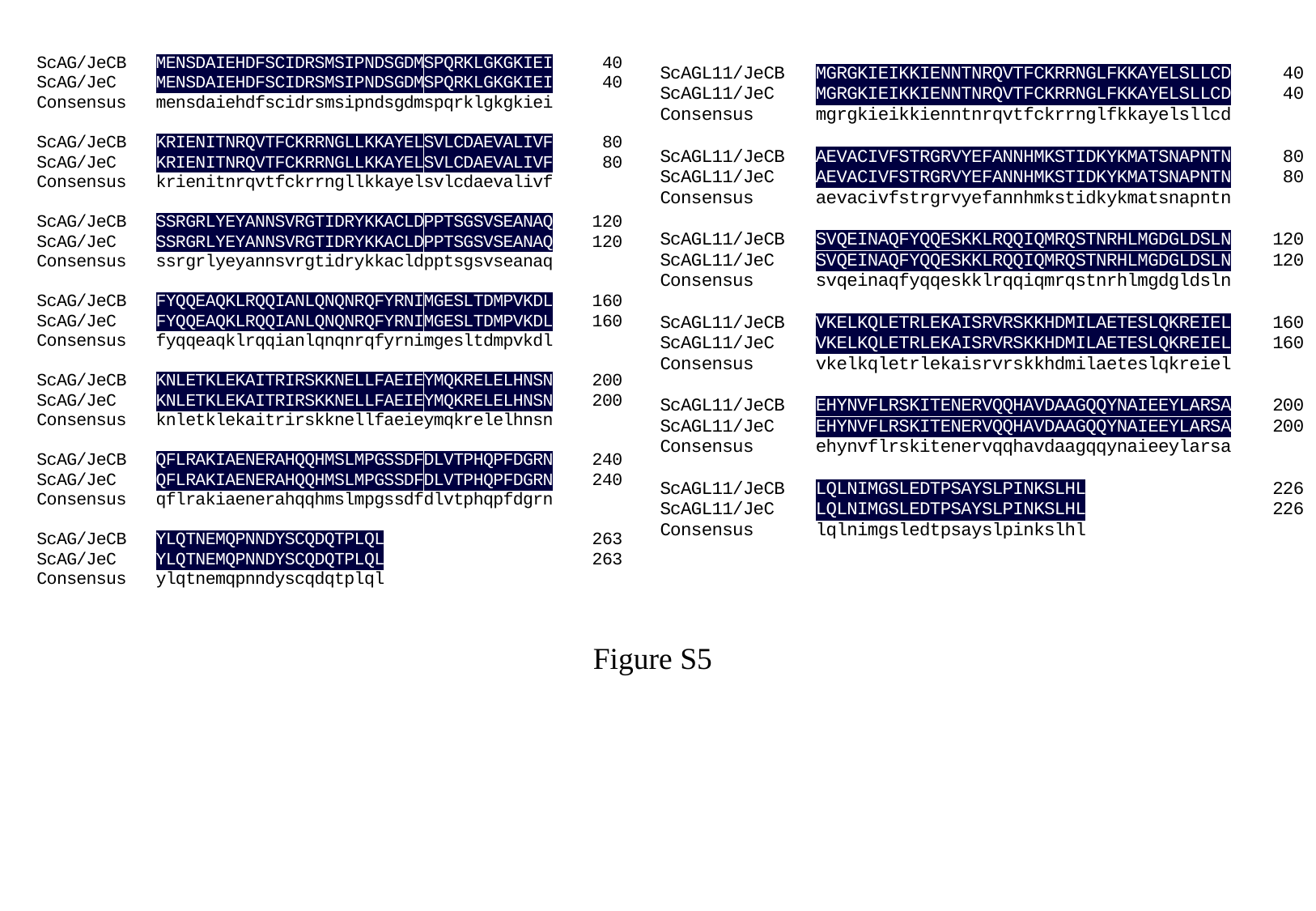

Figure S5

## Slide 13
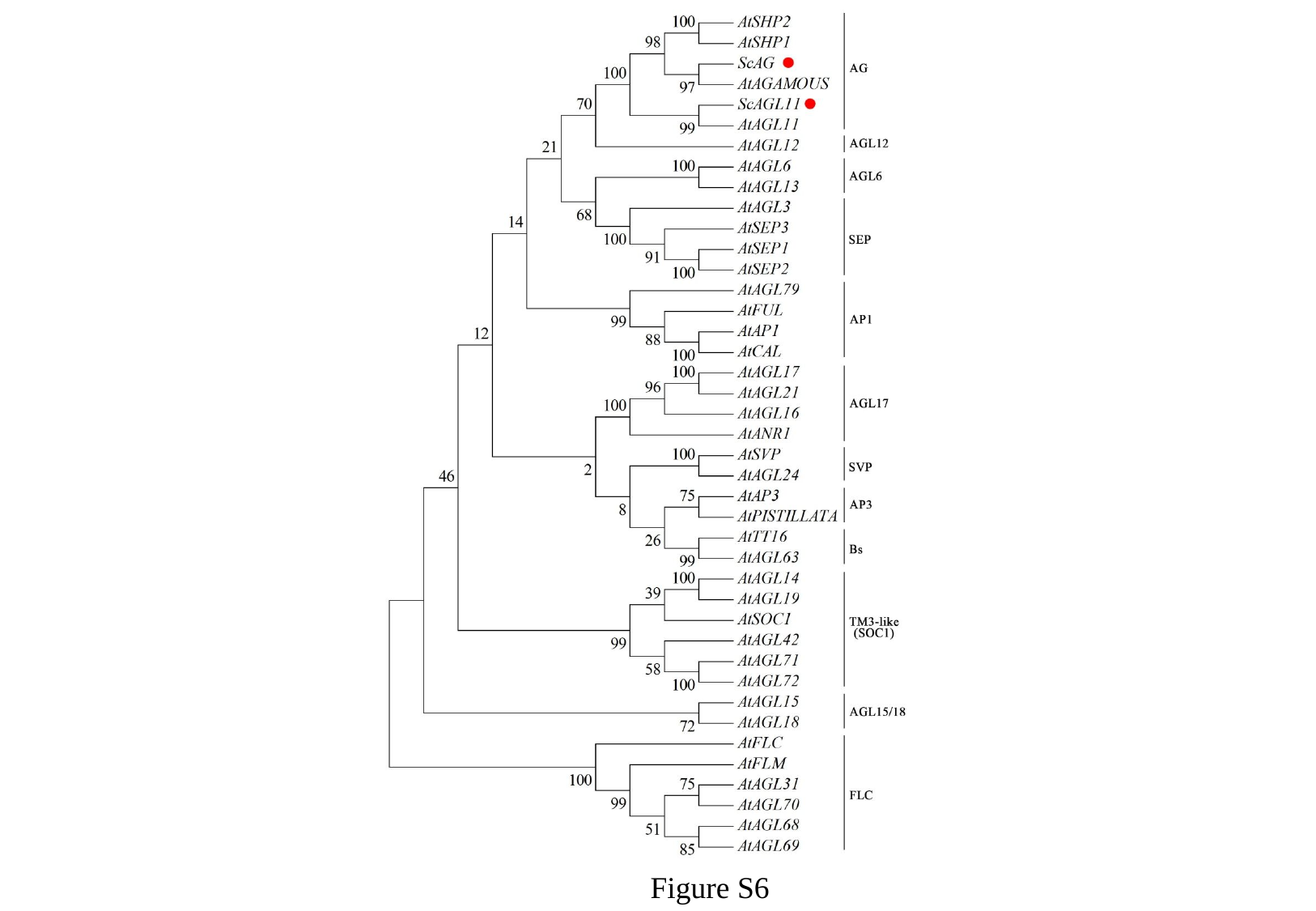

Figure S6

## Slide 14
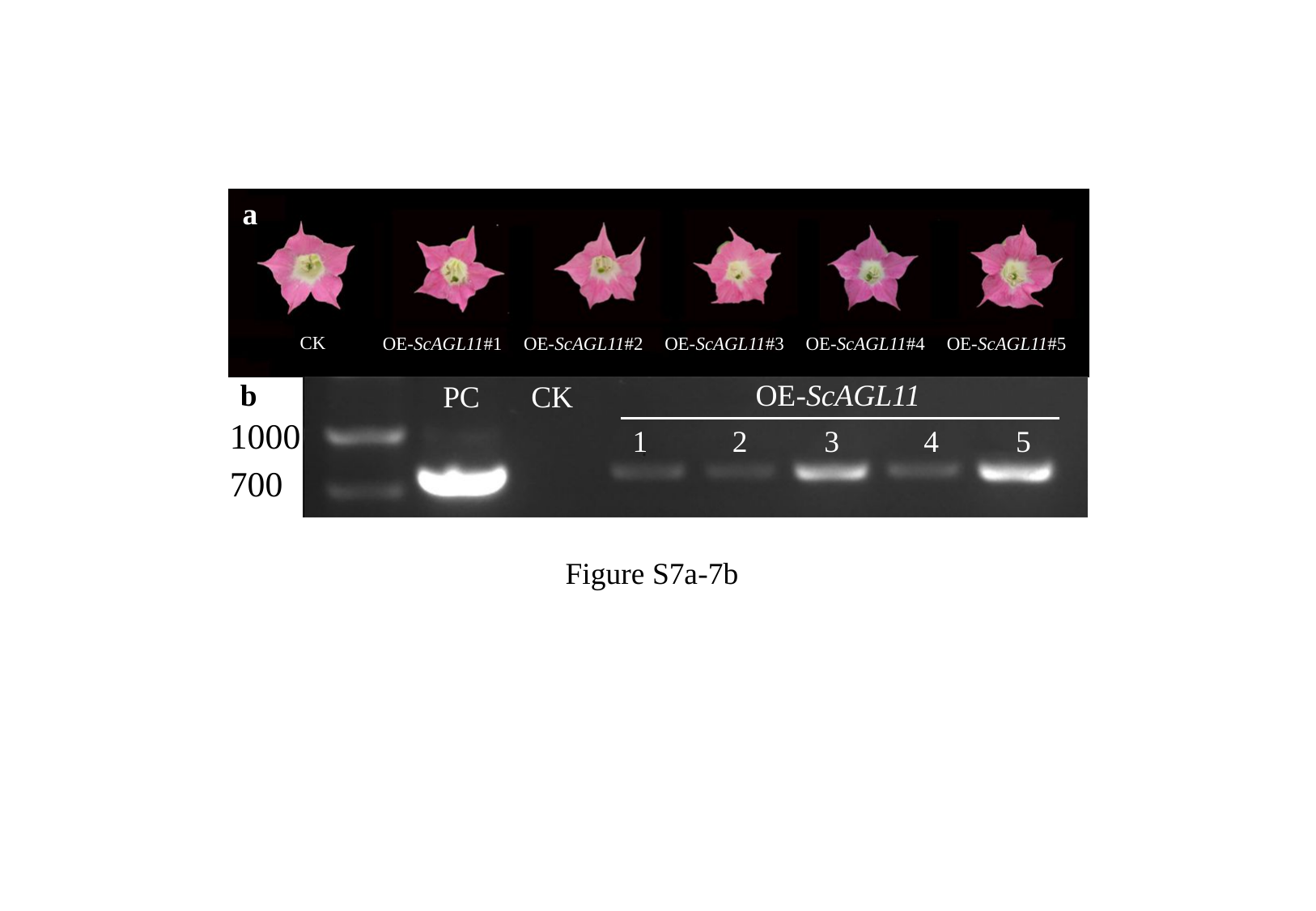

a
CK
OE-ScAGL11#1
OE-ScAGL11#2
OE-ScAGL11#3
OE-ScAGL11#4
OE-ScAGL11#5
OE-ScAGL11
b
PC
CK
1 2 3 4 5
1000
700
Figure S7a-7b

## Slide 15
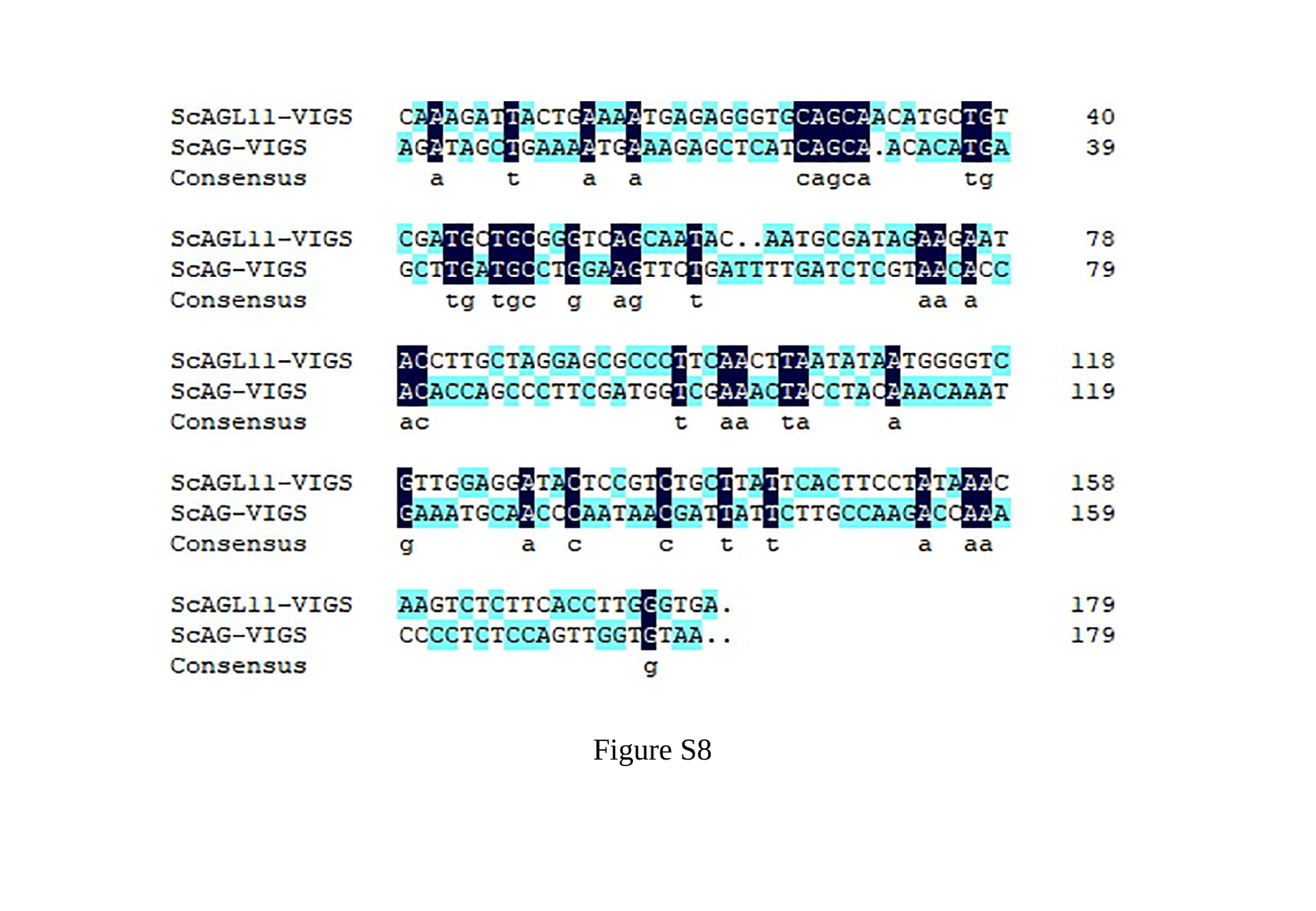

Figure S8

## Slide 16
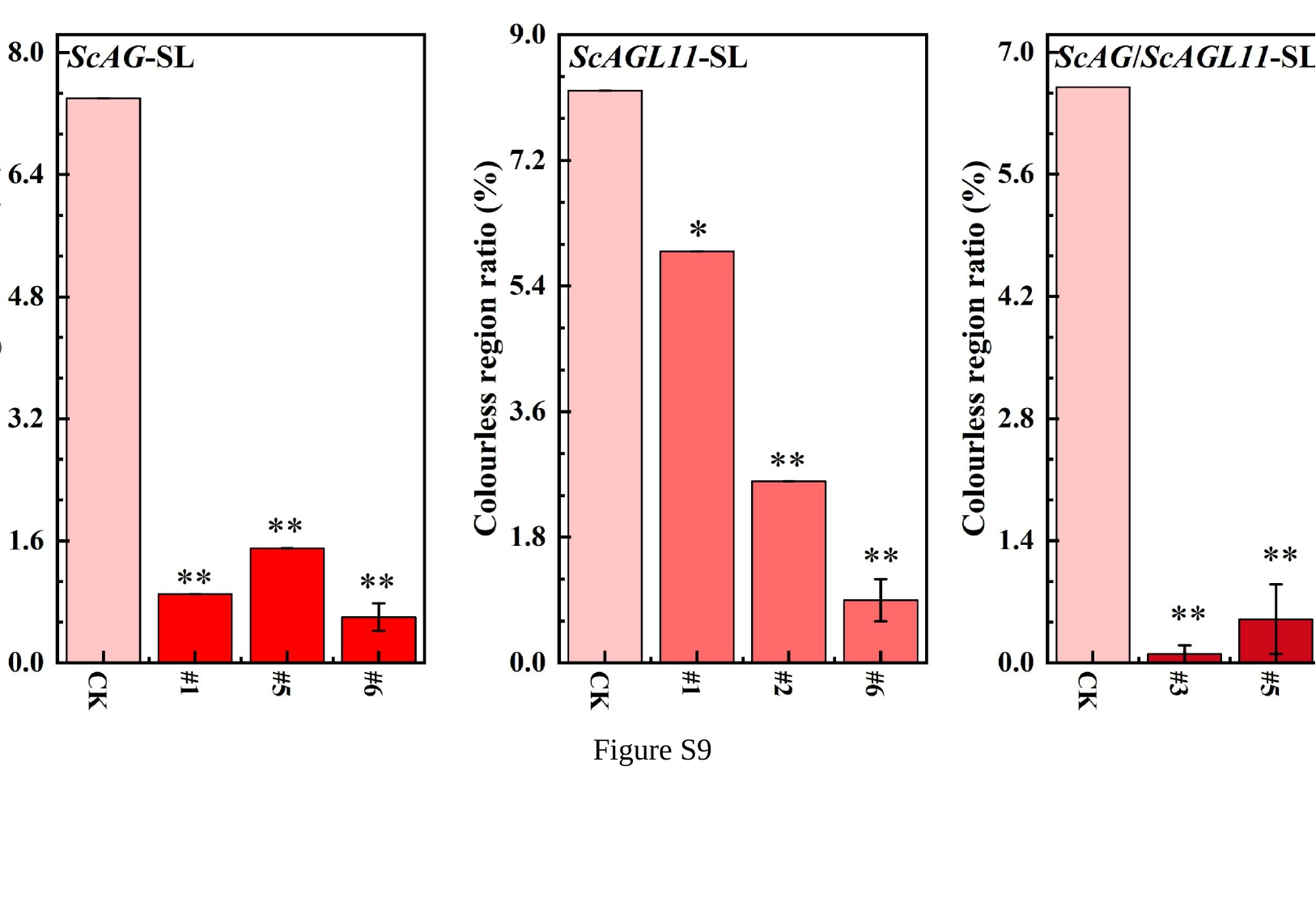

Figure S9

## Slide 17
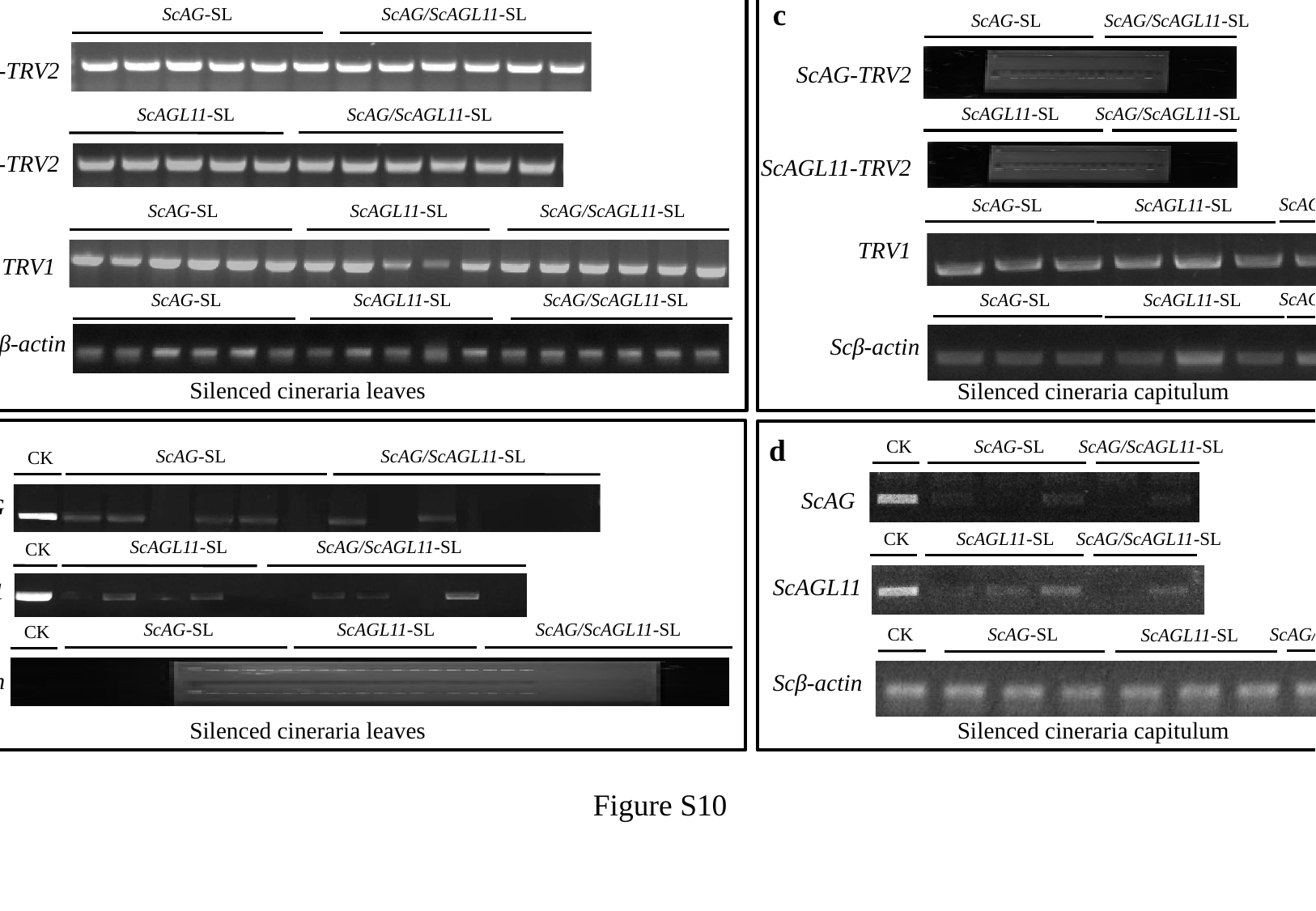

a
ScAG-SL
ScAG/ScAGL11-SL
ScAG-SL
ScAG/ScAGL11-SL
ScAG-TRV2
ScAG-TRV2
ScAGL11-SL
ScAG/ScAGL11-SL
ScAGL11-SL
ScAG/ScAGL11-SL
ScAGL11-TRV2
ScAGL11-TRV2
ScAG/ScAGL11-SL
ScAG-SL
ScAGL11-SL
ScAG-SL
ScAGL11-SL
ScAG/ScAGL11-SL
TRV1
TRV1
ScAG/ScAGL11-SL
ScAG-SL
ScAG-SL
ScAGL11-SL
ScAG/ScAGL11-SL
ScAGL11-SL
Scβ-actin
Scβ-actin
b
c
d
CK
ScAG-SL
ScAG/ScAGL11-SL
ScAG
CK
ScAGL11-SL
ScAG/ScAGL11-SL
ScAGL11
CK
ScAG/ScAGL11-SL
ScAG-SL
ScAGL11-SL
Scβ-actin
ScAG-SL
ScAG/ScAGL11-SL
CK
ScAG
ScAGL11-SL
ScAG/ScAGL11-SL
CK
ScAGL11
ScAG-SL
ScAGL11-SL
ScAG/ScAGL11-SL
CK
Scβ-actin
Silenced cineraria leaves
Silenced cineraria capitulum
Silenced cineraria leaves
Silenced cineraria capitulum
Figure S10

## Slide 18
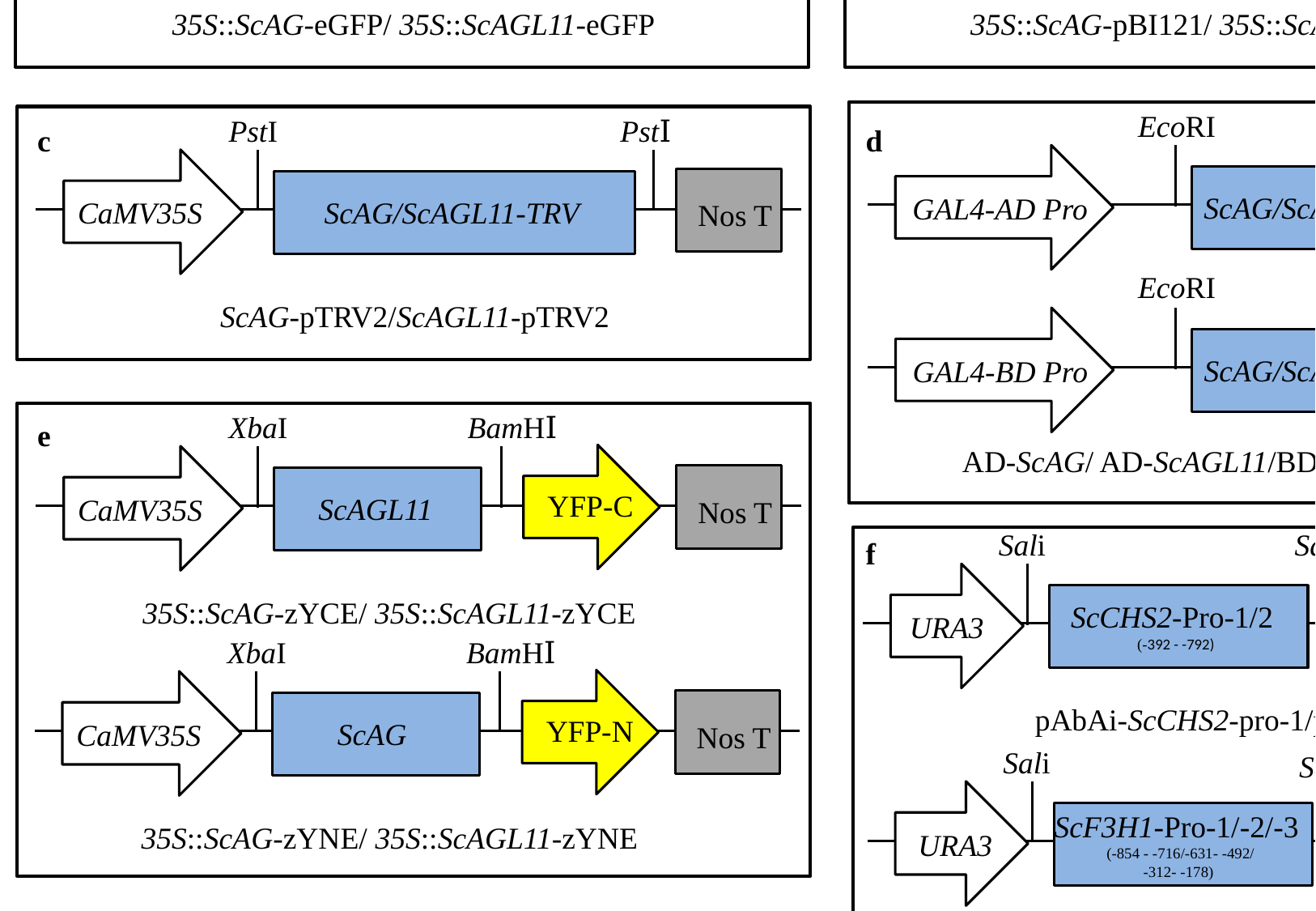

XhoI
Sali
CaMV35S
Nos T
ScAG/ScAGL11
eGFP
35S::ScAG-eGFP/ 35S::ScAGL11-eGFP
BamHⅠ
XbaI
CaMV35S
Nos T
ScAG/ScAGL11
GUS
35S::ScAG-pBI121/ 35S::ScAGL11-pBI121
a
b
BamHI
EcoRI
GAL4-AD Pro
Nos T
ScAG/ScAGL11
BamHI
EcoRI
GAL4-BD Pro
Nos T
ScAG/ScAGL11
AD-ScAG/ AD-ScAGL11/BD-ScAG/ BD-ScAGL11
PstⅠ
PstI
CaMV35S
Nos T
ScAG/ScAGL11-TRV
ScAG-pTRV2/ScAGL11-pTRV2
c
d
BamHⅠ
XbaI
CaMV35S
Nos T
ScAGL11
YFP-C
35S::ScAG-zYCE/ 35S::ScAGL11-zYCE
BamHⅠ
XbaI
CaMV35S
Nos T
ScAG
YFP-N
35S::ScAG-zYNE/ 35S::ScAGL11-zYNE
e
Saci
Sali
URA3
Nos T
ScCHS2-Pro-1/2
(-392 - -792)
AUR1-C
pAbAi-ScCHS2-pro-1/pro-2/pro-3
Sali
URA3
Nos T
ScF3H1-Pro-1/-2/-3
(-854 - -716/-631- -492/
-312- -178)
AUR1-C
pAbAi-ScF3H1-pro-1/pro-2/pro-3
Saci
URA3
Nos T
ScDFR3-Pro-1/-2
(-834- -713/-535- -407)
AUR1-C
pAbAi-ScDFR3-pro-1/pro-2
f
Saci
BamHⅠ
XbaI
CaMV35S
Nos T
ScAG/ScAGL11
ScAG-SK/ ScAGL11-SK
HindIIⅠ
KpnI
CaMV35S
Nos T
ScDFR3/ScF3H1-Pro
Luc
ScDFR3-Luc/ScF3H1-Luc
g
Sali
Figure S11a-11f
