# Supplementary material for: Functional analysis of the ScAG and ScAGL11 MADS-box transcription factors for anthocyanin biosynthesis and bicolour pattern formation in Senecio cruentus ray florets
Source: Hortic Res. 2022 Mar 23;9:uhac071. doi: 10.1093/hr/uhac071 (PMC9209810; doi:10.1093/hr/uhac071)
Supplement: Web_Material_uhac071 [file web_material_uhac071.zip › Minor revision Supplementary figures and tables.docx]

**Supplementary figures and tables**


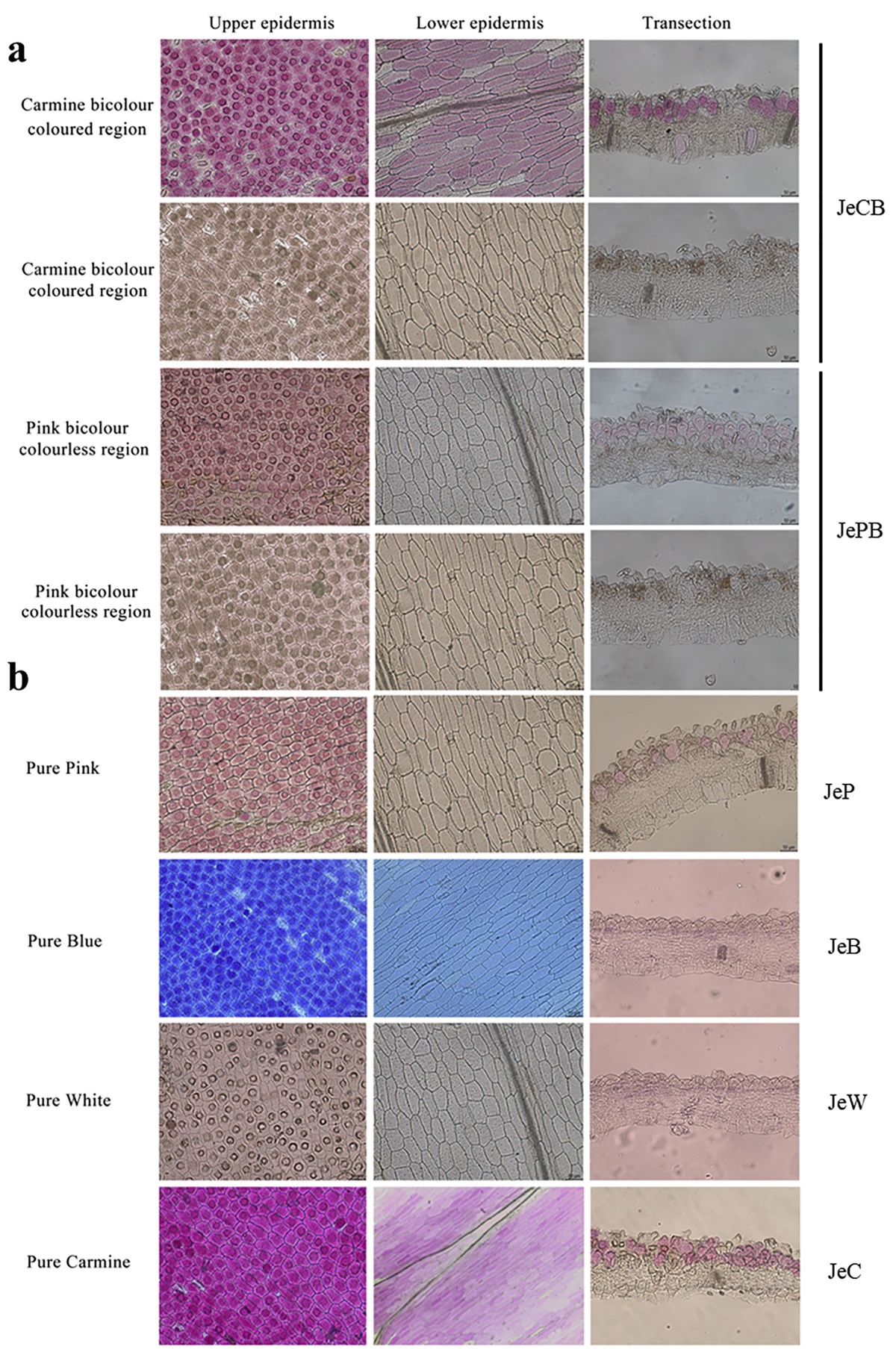


**Figure S1. Epidermal cell morphology in cineraria ray florets at S5 under an optical microscope (10×40).** **a.** Epidermal cell morphology in the colourless and coloured regions of JeCB and JePB ray florets. **b.** Epidermal cell morphology in four pure colour (JeP, JeB, JeW, and JeC) ray florets.


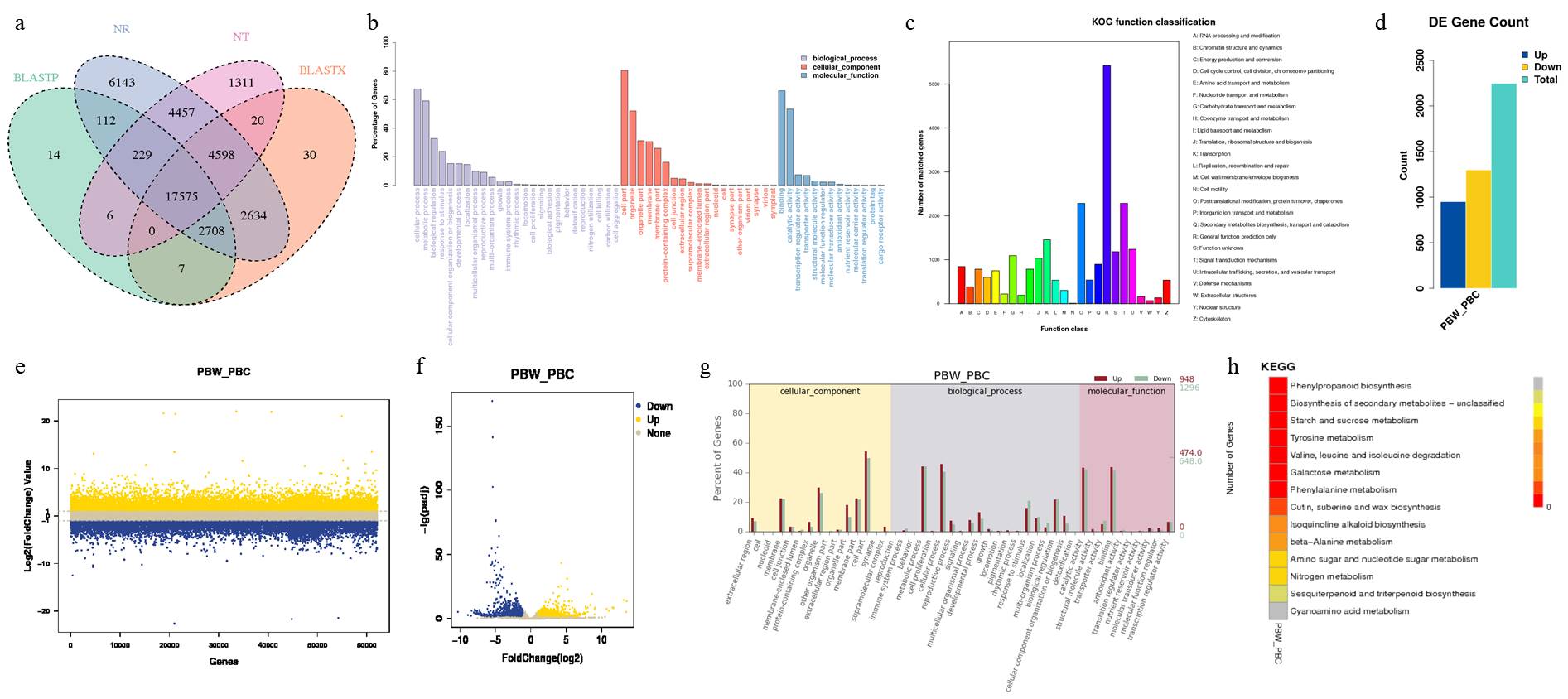


**Figure S2. Transcriptome analysis of cineraria.** **a.** Annotation results of 65144 unigenes according to four annotated databases. Green indicates BLASTP database, blue indicates NR database, pink indicates NT database, and orange colour indicates BLASTX database. **b.** GO annotation of 65144 unigenes. Unigenes were annotated in three major groups: biological process, cellular component, and molecular function. The right-side y-axis indicates the number of genes in a category; the left-side y-axis indicates the percentage of the genes in a specific category. **c.** KOG functional annotation of 65144 unigenes. Unigenes were analysed using the KOG database. The KOG classifications were divided into 24 functional groups, and 65144 unigenes were assigned to 24 KOG groups. **d.** Statistics results of 2246 unigenes according to the gene expression level. Up means up-regulated genes, and down means down-regulated genes. **e.** Gene differential expression in cineraria transcriptome. The figure shows the differentially expressed genes (DEGs) in the coloured and colourless region. The grey spots indicate unchanged; blue spots indicate down-regulated, and yellow spots indicate up-regulated unigenes. **f.** The volcano plot shows DEGs in the coloured and colourless regions. The grey spots indicate unchanged; blue spots indicate down-regulated, and yellow spots indicate up-regulated unigenes. **g.** GO annotation of DEGs. A total of 2246 DEGs were annotated to three major groups: biological process, cellular component, and molecular function. **h.** KEGG annotation of the DEGs. A total of 2246 annotated unigenes enriched 14 groups.


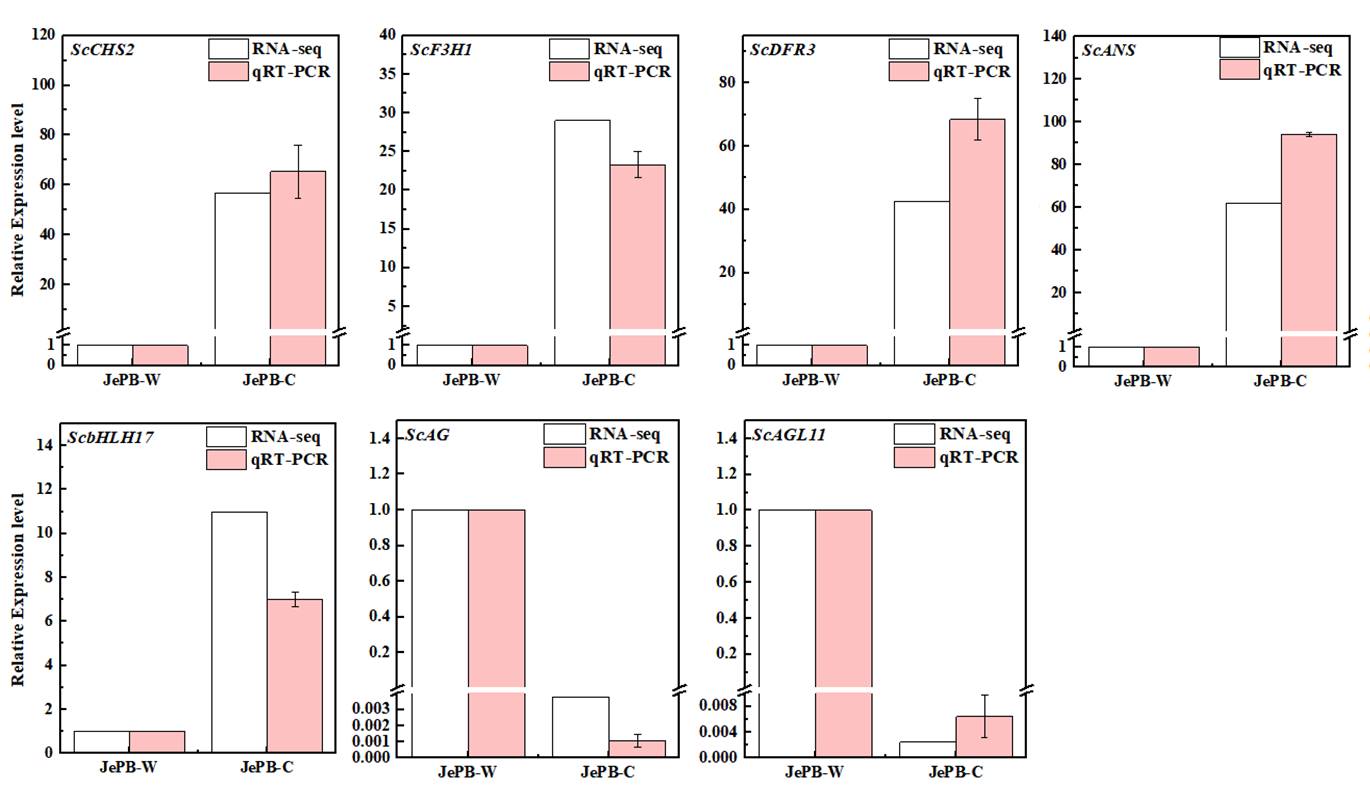


**Figure S3. Expression ratios of the seven DEGs from transcriptome analysis and qRT-PCR validation.** *ScCHS2*: TRINITY_DN11487_c0_g1, *ScF3H1*: TRINITY_DN15133_c1_g1, *ScDFR3*: TRINITY_DN17756_c4_g5, *ScANS*: TRINITY_DN19492_c0_g2, *ScbHLH17*: TRINITY_DN9621_c1_g1 *ScAG*: TRINITY_DN18674_c1_g1, *ScAGL11*: TRINITY_DN20125_c0_g1. RNA-seq: gene transcript accumulation level according to the transcriptome database. qRT-PCR: gene expression level from qRT-PCR validation. JePB-W: colourless region of JePB, JePB-C: coloured region of JePB.


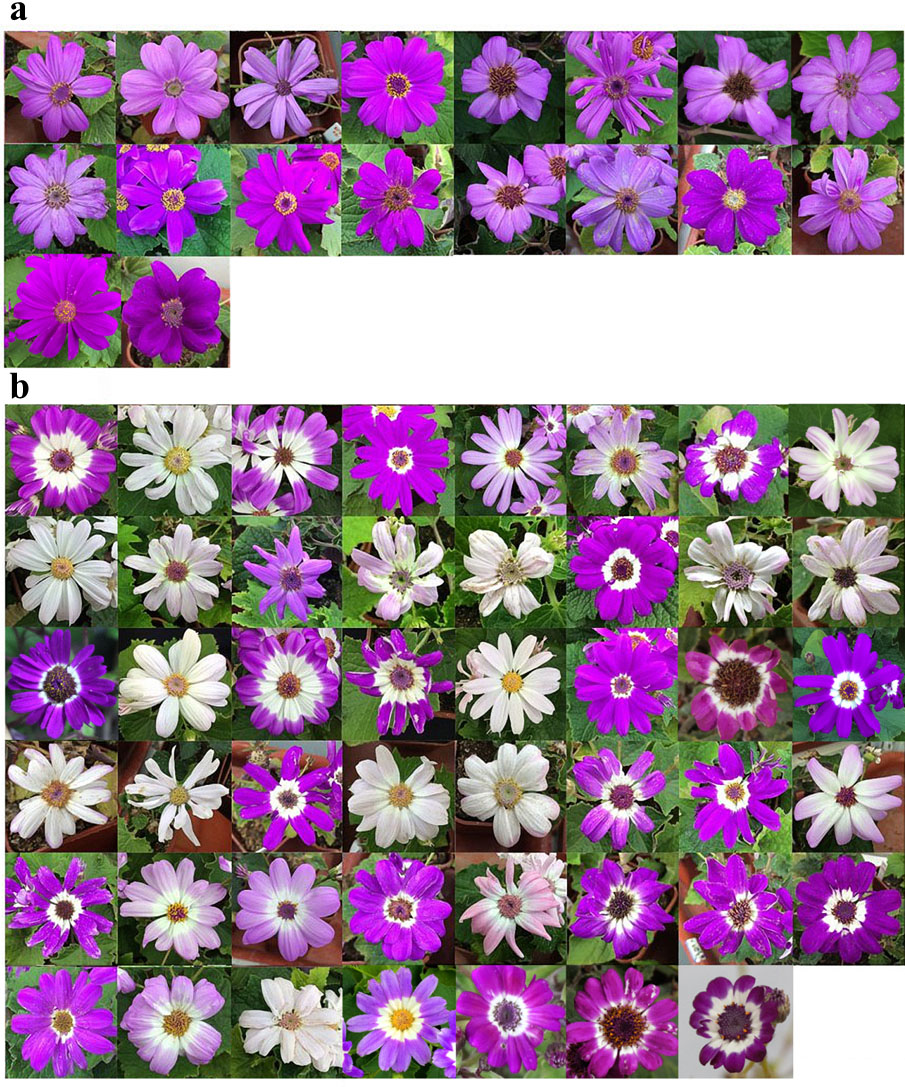


**Figure S4. Phenotypes of the F_1_ population (65) derived from JeW × JeCB crosses.** **a.** Pure carmine phenotypes (47) in the F_1_ population. **b.** Bicolour phenotypes (18) in the F_1_ population.

**
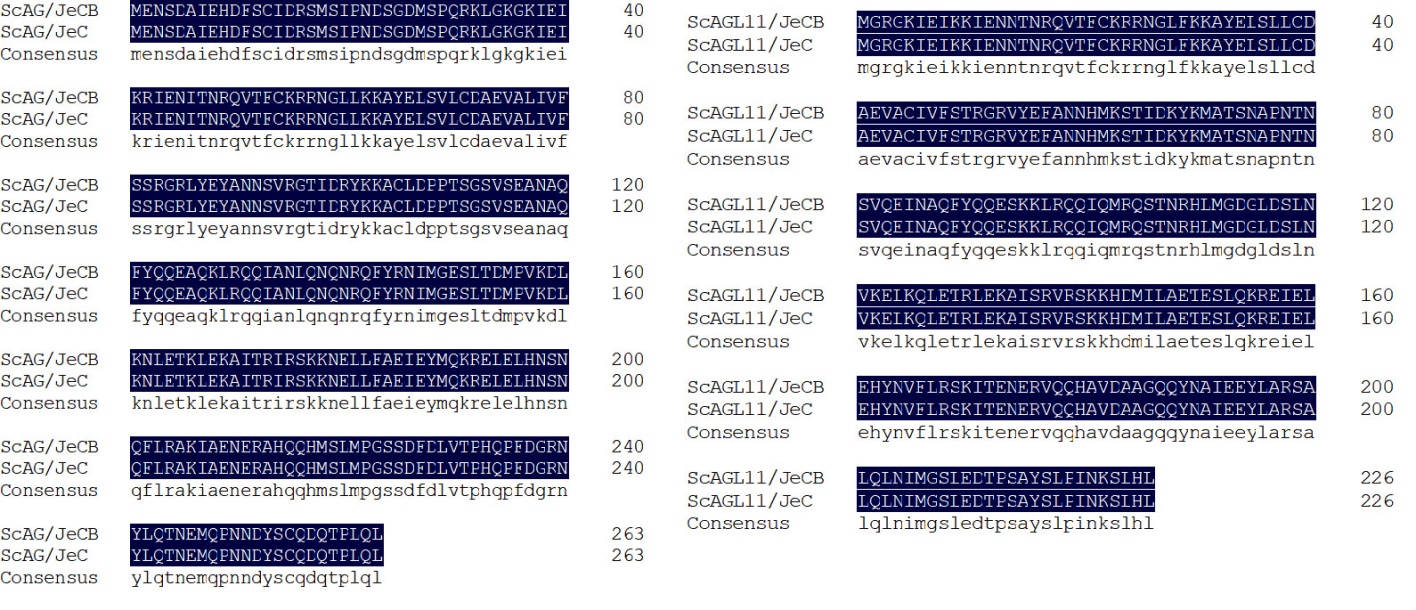
**

**Figure S5. The amino acid sequences of ScAG and ScAGL11 in the JeCB and JeC ray florets.**

**
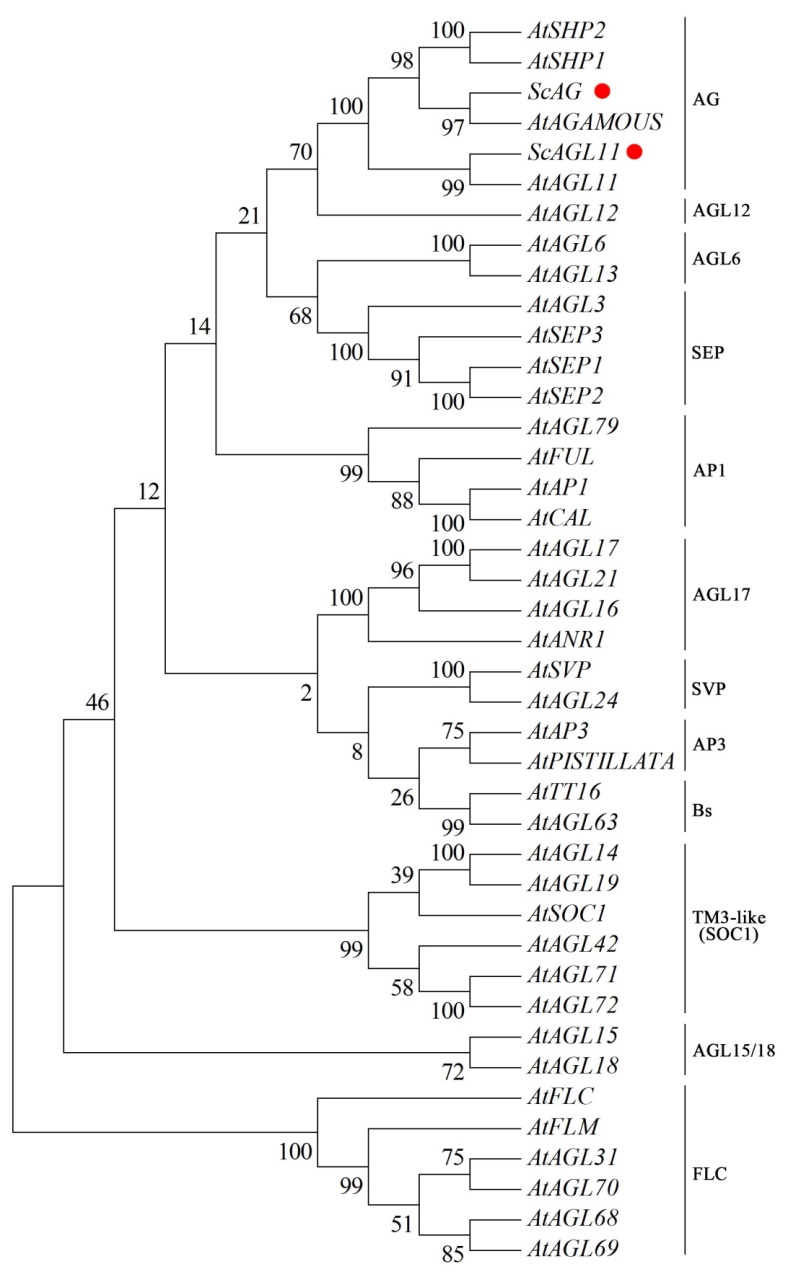
**

**Figure S6. A phylogenetic tree was built using ScAG, ScAGL11 and 39 MIKC type MADS-box TFs of *Arabidopsis*.** The red circle indicates ScAG and ScAGL11.


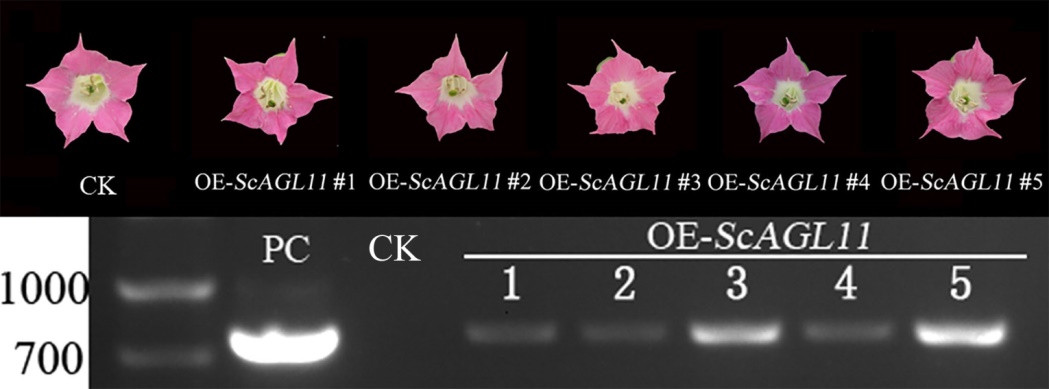


**Figure S7. Phenotypic results of *ScAGL11* expression in OE-*ScAGL11* tobacco lines.** CK: transgenic empty pBI121 vector tobacco. OE-*ScAGL11*: *ScAGL11*-overexpressed tobacco. PC: positive control, *A*. *tumefaciens* strain GV3101 containing *35S*::*ScAGL11*-pBI121 vector.


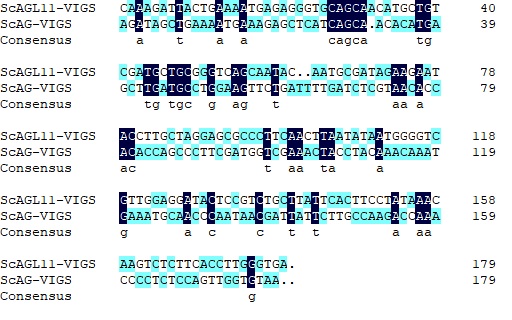


**Figure S8. Sequence alignment of the shortened fragments inserted into TRV2 vectors via the VIGS experiment.**


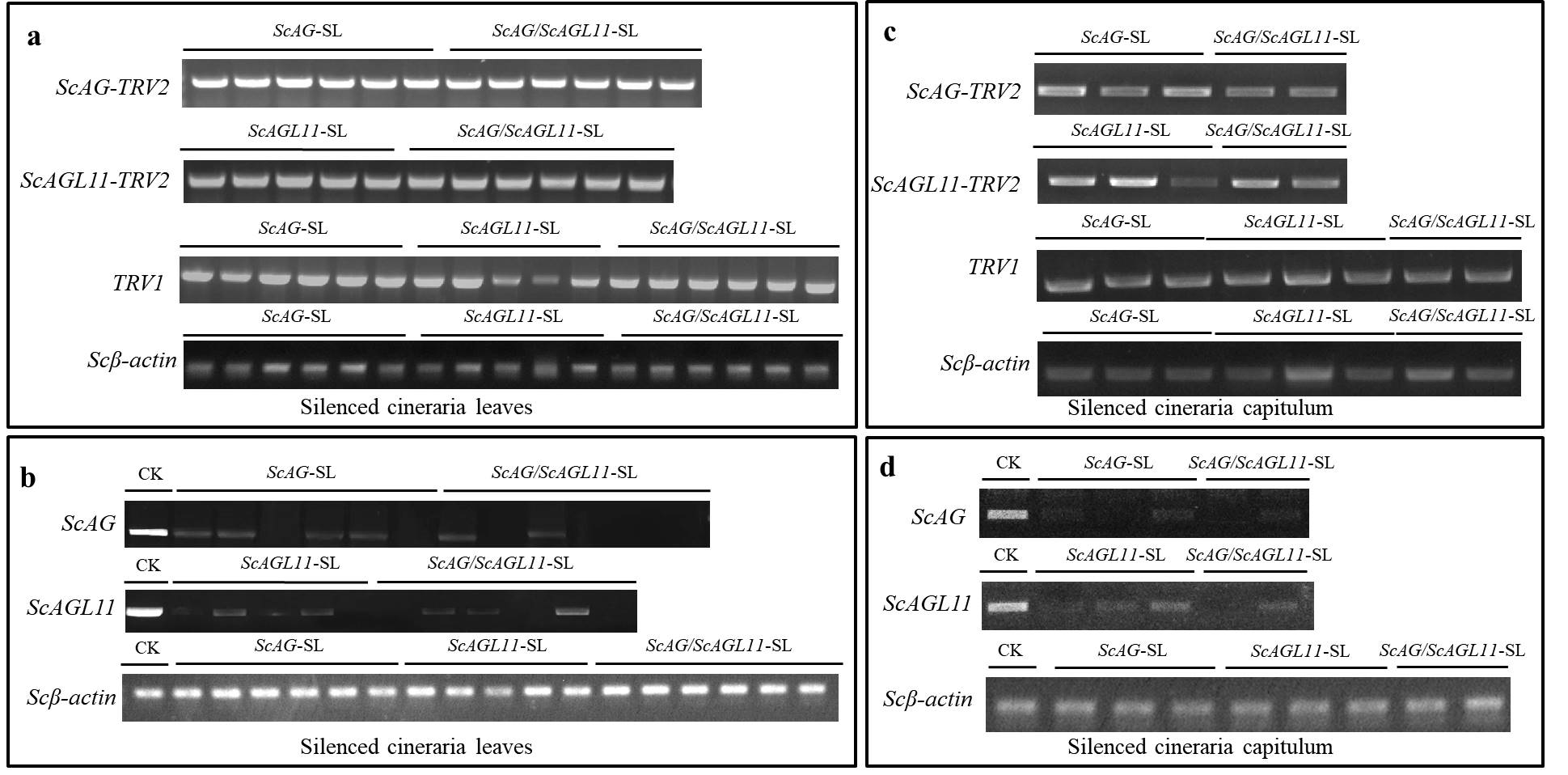


**Figure S9. Gene expression results from the silenced cineraria leaves and capitulum in VIGS experiments. a-b.** RT-PCR gene expression in the silenced cineraria leaves. **a.** Expression of *TRV1*, *ScAG*-*TRV2*, and *ScAGL11*-*TRV2* in silenced cineraria leaves. **b.** Expression of *ScAG* and *ScAGL11* in silenced cineraria leaves and the CK line. CK: leaves infiltrated with empty pTRV1 and pTRV2 vectors, *ScAG*-SL: *ScAG*-silenced cineraria leaves, *ScAGL11*-SL: *ScAGL11*-silenced cineraria leaves, *ScAG*/*ScAGL11*-SL: leaves where *ScAG* and *ScAGL11* were silenced together. **c-d.** RT-PCR gene expression in the silenced cineraria capitulum by RT-PCR **c.** Expression of *TRV1*, *ScAG*-*TRV2* and *ScAGL11*-*TRV2* in silenced cineraria capitulum and the CK line. **d.** Expression of *ScAG* and *ScAGL11* in silenced cineraria capitulum and the CK line. CK: capitulum infiltrated with empty pTRV1 and pTRV2 vectors, *ScAG*-SL: *ScAG*-silenced capitulum, *ScAGL11*-SL: *ScAGL11*-silenced capitulum, and *ScAG*/*ScAGL11*-SL: capitulum where *ScAG* and *ScAGL11* were silenced together.

**

**

**Figure S10. The ratio of the colourless region to the whole ray floret in the silenced cineraria capitulum.** *ScAG*-SL: *ScAG*-silenced ray florets, *ScAGL11*-SL: *ScAGL11*-silenced ray florets, *ScAG*/*ScAGL11*-SL: ray florets where *ScAG* and *ScAGL11* were silenced together. CK: capitulum infiltrated with empty pTRV1 and pTRV2 vectors. The data are presented as the mean ±SD from three biological replicates, *, *P*<0.05, **, *P*<0.01; Student’s *t*-test.


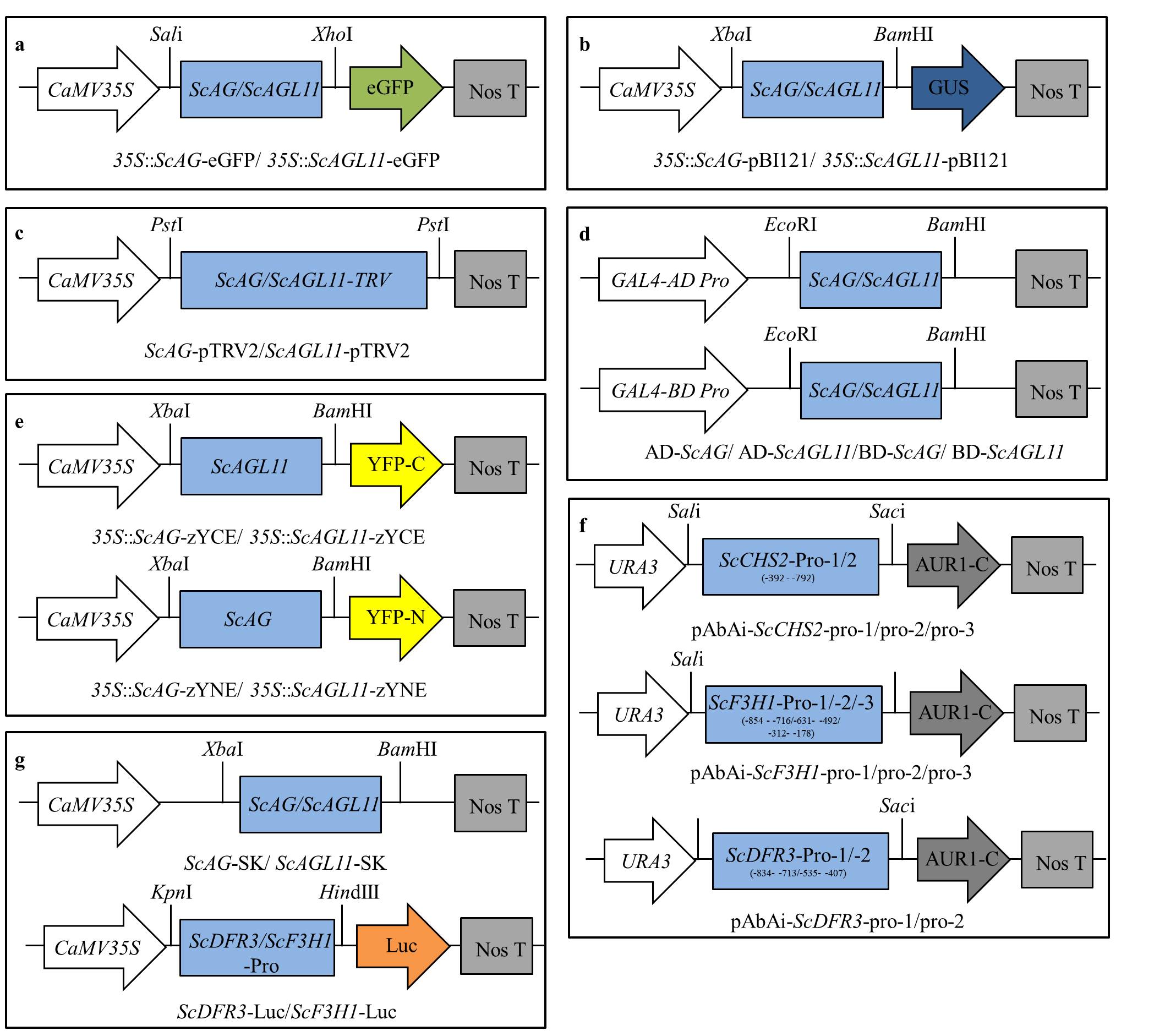


**Figure S11. Diagrams of recombinant constructs. a.** The *ScAG* and *ScAGL11* ORFs without the stop codon were inserted into eGFP vectors driven by the *CaMV35S* promoter using *Xho*I and *Sal*i restriction sites to generate *35S*::*ScAG*-eGFP and *35S*::*ScAGL11*-eGFP vectors. **b.** The *ScAG* and *ScAGL11* ORFs were cloned into pBI121 vectors driven by the *CaMV35S* promoter using *Xba*I and *Bam*HI restriction sites to generate *35S:*:*ScAG*-pBI121 and *35S*::*ScAGL11*-pBI121 vectors. **c.** Fragments of 178 bp and 179 bp from the non-conserved regions of the *ScAG* and *ScAGL11* sequences were linked with LIC1 (CGACGACAAGACCCT) and LIC2 (GAGGAGAAGAGCCCT). The linked were inserted into pTRV2 vectors using the *Pst*I restriction site to obtain *ScAG*-pTRV2 and *ScAGL11*-pTRV2 vectors. **d.** The *ScAG* and *ScAGL11* ORFs were inserted into pGADT7 and pGBKT7 vectors to obtain the AD-*ScAG*, AD-*ScAGL11*, BD-*ScAG*, and BD-*ScAGL11*. **e.** The *ScAG* ORFs (without the stop codon) was inserted into the N-terminal region of yellow fluorescent protein (YFP), and the ORF of *ScAGL11* (without the stop codon) was linked into the C-terminal region to construct *35S*::Sc*AG*-zYNE and *35S*::Sc*AGL11*-zYCE vectors**. f.** The shortened fragments containing different CArG boxes in the *ScCHS2*, *ScF3H1*, and *ScDFR3* promoters were inserted into pAbAi vectors using *Sac*i and *Sal*i restriction sites to construct pAbAi-*ScCHS2*-pro-1/pro-2/pro-3, pAbAi-*ScF3H1*-pro-1/pro-2/pro-3 and pAbAi-*ScDFR3*-pro-1/pro-2 vectors. **g.** The *ScAG* and *ScAGL11* ORFs were inserted into pGreenⅡ62-SK vectors to generate *ScAG*-SK and *ScAGL11*-SK vectors. The sequences of *ScF3H1* and *ScDFR3* were cloned into pGreenⅡ62-Luc vectors to generate *ScF3H1*-Luc and *ScDFR3*- Luc vectors.

**Table S1. Anthocyanidins composition in cineraria ‘Jester’ bicolour cultivars.**

| Cultivar | Ingredient | Retention time (min) | Mass spectrum result | Anthocyanidn |
| --- | --- | --- | --- | --- |
| JeCB-C | C1 | 19.4 | 287,449,611,773 | cyanidin |
|  | C2 | 21.5 | 287,449,611,773,919 | cyanidin |
|  | C3 | 25.5 | 303,465,627,789,951 | delphinidin |
| JePB-C | P1 | 16.0 | 271,433,595,757,875,919 | pelargonidin |
|  | P2 | 21.7 | 287,449,611,773,859,935 | pelargonidin |
|  | P3 | 23.9 | 271,433,519,757 | cyanidin |
|  | P4 | 32.6 | 271,433,757,919 | pelargonidin |

JeCB-C: coloured region of JeCB, JePB-C: coloured region of JePB

**Table S2. Summary of transcriptome sequence results in JePB**

| **Sample** | **JePB-W-1** | **JePB-W-2** | **JePB-W-3** | **JePB-C-1** | **JePB-C-2** | **JePB-C-3** | **Total** |
| --- | --- | --- | --- | --- | --- | --- | --- |
| **Raw Reads Number** | 37710646 | 41299084 | 39800770 | 38928424 | 42,175,114 | 42,175,115 | 38928424 |
| **Raw Bases Number** | 5656596900 | 6194862600 | 5970115500 | 5839263600 | 6,326,267,100 | 6,326,267,101 | 5839263600 |
| **Clean Reads Number** | 36273976 | 39507050 | 37821048 | 37201112 | 40,204,884 | 40,204,885 | 37201112 |
| **Clean Reads Rate(%)** | 96.19 | 95.66 | 95.03 | 95.56 | 95.33 | 96.33 | 95.56 |
| **Clean Bases Number** | 5441096400 | 5926057500 | 5673157200 | 5580166800 | 6,030,732,600 | 6,030,732,601 | 5580166800 |
| **Low-quality Reads Number** | 563072 | 755128 | 736054 | 550038 | 748,940 | 748,941 | 550038 |
| **Low-quality Reads Rate(%)** | 1.49 | 1.83 | 1.85 | 1.41 | 1.78 | 2.78 | 1.41 |
| **Ns Reads Number** | 85828 | 128582 | 139940 | 133278 | 144,504 | 144,505 | 133278 |
| **Ns Reads Rate(%)** | 0.23 | 0.31 | 0.35 | 0.34 | 0.34 | 1.34 | 0.34 |
| **Adapter Polluted Reads Number** | 787770 | 908324 | 1103728 | 1043996 | 1,076,786 | 1,076,787 | 1043996 |
| **Adapter Polluted Reads Rate(%)** | 2.09 | 2.2 | 2.77 | 2.68 | 2.55 | 3.55 | 2.68 |
| **Raw Q30 Bases Rate(%)** | 88.76 | 88.55 | 88.77 | 88.9 | 88.84 | 89.84 | 88.9 |
| **Clean Q30 Bases Rate(%)** | 89.32 | 89.22 | 89.47 | 89.46 | 89.52 | 90.52 | 89.46 |
| **Total unigene number** | 47588 | 50489 | 49797 | 49793 | 49318 | 49319 | 65144 |

JePB-W: colourless region of JePB, JePB-C: coloured region of JePB.

**Table S3. Transcriptome sequence assembly for JePB**

| **Header** | **Trinity** | **Unigene** |
| --- | --- | --- |
| **Count** | 213951 | 65144 |
| **Percent GC (%)** | 38.70 | 38.09 |
| **Total bases** | 220607256 | 57218168 |
| **N50** | 1526 | 1419 |
| **N90** | 473 | 362 |
| **Min** | 201 | 201 |
| **Max** | 16589 | 16589 |
| **Count** | 213951 | 65144 |
| **Mean** | 103111 | 87833 |

**Table S4. Unigenes were annotated in 13 databases.**

| **Database** | **Count** | **Percentage (%)** |
| --- | --- | --- |
| **NR** | 38456 | 59.032 |
| **GO** | 27592 | 42.355 |
| **TmHMM** | 5349 | 8.211 |
| **SignalP** | 1586 | 2.435 |
| **NT** | 28196 | 43.283 |
| **Prot** | 26291 | 40.358 |
| **KO** | 10934 | 16.784 |
| **RNAMMER** | 18 | 0.028 |
| **BLASTP** | 20651 | 31.701 |
| **eggNOG** | 23042 | 35.371 |
| **Map** | 6960 | 10.684 |
| **PFAM** | 19008 | 29.178 |
| **BLASTX** | 27572 | 42.325 |
| **Total_anno** | 65144 | 100 |
| **Total_unigene** | 65144 | 100 |

**Table S5. List of 386 DEGs in the colourless compared to the coloured region in JePB.**

|  | **Gene** | **FPKM** | | **Log_2_FoldChange** | **Up/Down** | **NR_Description** |
| --- | --- | --- | --- | --- | --- | --- |
|  |  | **JePB-W** | **JePB-C** |  |  |  |
| **1** | TRINITY_DN11859_c1_g4 | 4 | 51 | -3.593 | down | hypothetical protein E3N88_26614 (*Mikania micrantha*) |
| **2** | TRINITY_DN7687_c0_g1 | 12 | 122 | -3.368 | down | uncharacterized protein At5g65660-like (*Cynara cardunculus* var. *scolymus*) |
| **3** | TRINITY_DN6737_c0_g1 | 4 | 93 | -4.715 | down | basic-leucine zipper domain-containing protein (*Artemisia annua*) |
| **4** | TRINITY_DN9534_c0_g2 | 3 | 65 | -4.470 | down | hypothetical protein E3N88_15396 (*Mikania micrantha*) |
| **5** | TRINITY_DN11264_c1_g4 | 8 | 87 | -3.414 | down | Cilia- and flagella-associated protein 20 (*Glycine soja*) |
| **6** | TRINITY_DN14114_c0_g4 | 6 | 69 | -3.491 | down | hypothetical protein E3N88_24673 (*Mikania micrantha*) |
| **7** | TRINITY_DN11693_c0_g3 | 2 | 155 | -6.106 | down | putative transcription factor interactor and regulator CCHC(Zn) family (*Helianthus annuus*) |
| **8** | TRINITY_DN13566_c0_g2 | 4 | 274 | -6.019 | down | glycine-rich protein 2 isoform X3 (*Cynara cardunculus* var. *scolymus*) |
| **9** | TRINITY_DN13133_c4_g2 | 3 | 32 | -3.638 | down | pyridoxal kinase-like isoform X6 (*Lactuca sativa*) |
| **10** | TRINITY_DN7403_c0_g3 | 1 | 29 | -4.456 | down | 7-ethoxycoumarin O-deethylase-like (*Cynara cardunculus* var. *scolymus*) |
| **11** | TRINITY_DN8193_c1_g2 | 7 | 151 | -4.393 | down | cytochrome P450 71B34-like (*Lactuca sativa*) |
| **12** | TRINITY_DN5596_c0_g1 | 3 | 71 | -4.597 | down | mechanosensitive ion channel protein 10 isoform X1 (*Helianthus annuus*) |
| **13** | TRINITY_DN11693_c0_g1 | 13 | 320 | -4.674 | down | uncharacterized protein LOC112508939 (*Cynara cardunculus* var. *scolymus*) |
| **14** | TRINITY_DN6241_c0_g1 | 9 | 115 | -3.655 | down | probable purine permease 11 (*Helianthus annuus*) |
| **15** | TRINITY_DN11523_c1_g2 | 0.7 | 12 | -4.237 | down | abscisic acid receptor PYL9 (*Cynara cardunculus* var. *scolymus*) |
| **16** | TRINITY_DN5743_c0_g2 | 2 | 20 | -3.625 | down | hypothetical protein E3N88_45523 (*Mikania micrantha*) |
| **17** | TRINITY_DN10042_c1_g2 | 2 | 23 | -3.574 | down | LEAF RUST 10 DISEASE-RESISTANCE LOCUS RECEPTOR-LIKE PROTEIN KINASE-like 1.1 (*Helianthus annuus*) |
| **18** | TRINITY_DN10488_c1_g2 | 2 | 49 | -4.434 | down | SGNH hydrolase-type esterase domain-containing protein (*Artemisia annua*) |
| **19** | TRINITY_DN18991_c4_g1 | 4 | 47 | -3.595 | down | phytosulfokine (*Artemisia annua*) |
| **20** | TRINITY_DN19823_c1_g2 | 2 | 46 | -4.342 | down | uncharacterized protein LOC112521824 (*Cynara cardunculus* var. *scolymus*) |
| **21** | TRINITY_DN13717_c1_g3 | 1.0 | 18 | -4.210 | down | putative transcription elongation factor SPT5 homolog 1 isoform X3 (*Cynara cardunculus* var. *scolymus*) |
| **22** | TRINITY_DN13622_c2_g7 | 0.7 | 33 | -5.675 | down | branched-chain amino acid aminotransferase 2, chloroplastic isoform X2 (*Helianthus annuus*) |
| **23** | TRINITY_DN9782_c4_g3 | 5 | 95 | -4.399 | down | Cystinosin/ERS1p repeat-containing protein (*Artemisia annua*) |
| **24** | TRINITY_DN11121_c1_g2 | 0.3 | 20 | -5.822 | down | auxin-responsive protein IAA29-like (*Cynara cardunculus* var. *scolymus*) |
| **25** | TRINITY_DN16498_c2_g3 | 0.3 | 52 | -7.209 | down | hypothetical protein HanXRQr2_Chr07g0294841 (*Helianthus annuus*) |
| **26** | TRINITY_DN9101_c2_g1 | 0.7 | 41 | -5.928 | down | unnamed protein product (*Lactuca saligna*) |
| **27** | TRINITY_DN9236_c1_g1 | 2 | 49 | -4.633 | down | hypothetical protein E3N88_35266 (*Mikania micrantha*) |
| **28** | TRINITY_DN10315_c1_g4 | 11 | 152 | -3.843 | down | hypothetical protein E3N88_41627 (*Mikania micrantha*) |
| **29** | TRINITY_DN10237_c0_g3 | 9 | 88 | -3.324 | down | serine/threonine-protein kinase-like protein CCR4 isoform X1 (*Lactuca sativa*) |
| **30** | TRINITY_DN17803_c0_g1 | 12 | 122 | -3.434 | down | NAC domain-containing protein 35 (*Cynara cardunculus* var. *scolymus*) |
| **31** | TRINITY_DN11882_c0_g2 | 10 | 135 | -3.811 | down | G-type lectin S-receptor-like serine/threonine-protein kinase At4g27290 (*Helianthus annuus*) |
| **32** | TRINITY_DN808_c0_g1 | 3 | 39 | -3.935 | down | abscisic acid receptor PYL4 (*Helianthus annuus*) |
| **33** | TRINITY_DN9813_c1_g1 | 3 | 117 | -5.203 | down | transmembrane amino acid transporter family protein (*Artemisia annua*) |
| **34** | TRINITY_DN12205_c1_g1 | 19 | 294 | -4.043 | down | inositol 2-dehydrogenase 2 (*Helianthus annuus*) |
| **35** | TRINITY_DN6430_c0_g2 | 0.7 | 41 | -6.012 | down | unnamed protein product (*Lactuca saligna*) |
| **36** | TRINITY_DN1150_c0_g2 | 0.3 | 17 | -5.602 | down | organic cation/carnitine transporter4 (*Artemisia annua*) |
| **37** | TRINITY_DN13076_c2_g10 | 0.3 | 30 | -6.423 | down | hypothetical protein E3N88_25389 (*Mikania micrantha*) |
| **38** | TRINITY_DN11782_c0_g2 | 0.3 | 35 | -6.662 | down | cytochrome P450 93A3-like (*Cynara cardunculus* var. *scolymus*) |
| **39** | TRINITY_DN923_c0_g2 | 0.3 | 77 | -7.807 | down | beta-glucosidase 9 GH1 family (*Artemisia annua*) |
| **40** | TRINITY_DN11101_c1_g4 | 3 | 32 | -3.698 | down | hypothetical protein E3N88_31130 (*Mikania micrantha*) |
| **41** | TRINITY_DN17874_c1_g2 | 2 | 25 | -4.008 | down | hypothetical protein E3N88_25847 (*Mikania micrantha*) |
| **42** | TRINITY_DN7840_c0_g1 | 1.0 | 101 | -6.738 | down | heme peroxidase (*Artemisia annua*) |
| **43** | TRINITY_DN5051_c0_g1 | 2 | 42 | -4.748 | down | hypothetical protein LSAT_9X79900 (*Lactuca sativa*) |
| **44** | TRINITY_DN2834_c0_g1 | 0.7 | 96 | -7.229 | down | 21 kDa protein (*Helianthus annuus*) |
| **45** | TRINITY_DN12747_c1_g3 | 0.3 | 18 | -5.720 | down | hypothetical protein HanXRQr2_Chr06g0259871 (*Helianthus annuus*) |
| **46** | TRINITY_DN1370_c0_g1 | 0.3 | 17 | -5.616 | down | acyl transferase/acyl hydrolase/lysophospholipase (*Artemisia annua*) |
| **47** | TRINITY_DN4379_c0_g1 | 0.7 | 21 | -5.021 | down | neomenthol dehydrogenase-like (*Lactuca sativa*) |
| **48** | TRINITY_DN4867_c0_g1 | 0.7 | 31 | -5.603 | down | hypothetical protein CTI12_AA458930 (*Artemisia annua*) |
| **49** | TRINITY_DN5251_c0_g1 | 0.3 | 13 | -5.247 | down | unnamed protein product (*Lactuca saligna*) |
| **50** | TRINITY_DN10289_c0_g4 | 0.3 | 53 | -7.270 | down | uncharacterized protein LOC110897625 (*Helianthus annuus*) |
| **51** | TRINITY_DN5127_c0_g1 | 0.3 | 64 | -7.555 | down | hypothetical protein CTI12_AA514550 (*Artemisia annua*) |
| **52** | TRINITY_DN5252_c0_g1 | 0.3 | 20 | -5.872 | down | START-like domain, Bet v I type allergen (*Artemisia annua*) |
| **53** | TRINITY_DN16968_c0_g4 | 0.3 | 17 | -5.610 | down | putative chromatin regulator PHD family (*Helianthus annuus*) |
| **54** | TRINITY_DN8760_c1_g2 | 8 | 126 | -4.032 | down | hypothetical protein E3N88_23088 (*Mikania micrantha*) |
| **55** | TRINITY_DN9636_c0_g2 | 21 | 226 | -3.467 | down | Pleckstrin homology-like domain-containing protein (*Cynara cardunculus* var. *scolymus*) |
| **56** | TRINITY_DN9878_c1_g2 | 2 | 33 | -3.897 | down | dicer-like 4 (*Artemisia annua*) |
| **57** | TRINITY_DN12059_c1_g1 | 605 | 9935 | -4.089 | down | beta-D-xylosidase 1 (*Helianthus annuus*) |
| **58** | TRINITY_DN5014_c0_g1 | 2 | 42 | -4.433 | down | putative UPF0481 protein At3g02645 (*Helianthus annuus*) |
| **59** | TRINITY_DN7194_c0_g1 | 3 | 60 | -4.556 | down | proteinase inhibitor I25 (*Artemisia annua*) |
| **60** | TRINITY_DN15360_c3_g6 | 7 | 101 | -3.901 | down | uncharacterized protein LOC111906747 (*Lactuca sativa*) |
| **61** | TRINITY_DN8102_c2_g1 | 0.3 | 43 | -6.937 | down | putative GDSL lipase/esterase, SGNH hydrolase superfamily (*Helianthus annuus*) |
| **62** | TRINITY_DN9284_c1_g1 | 0.3 | 68 | -7.605 | down | uncharacterized protein LOC110874213 (*Helianthus annuus*) |
| **63** | TRINITY_DN9095_c1_g1 | 3 | 44 | -3.920 | down | hypothetical protein E3N88_23225 (*Mikania micrantha*) |
| **64** | TRINITY_DN17360_c1_g4 | 0.7 | 23 | -5.173 | down | polygalacturonase At1g48100 (*Helianthus annuus*) |
| **65** | TRINITY_DN10276_c0_g2 | 0.7 | 12 | -4.247 | down | Heat shock protein 70 family (*Artemisia annua*) |
| **66** | TRINITY_DN12244_c0_g3 | 1 | 30 | -4.983 | down | protein kinase domain-containing protein (*Artemisia annua*) |
| **67** | TRINITY_DN6396_c0_g1 | 0.3 | 15 | -5.467 | down | transcription factor TGA9 isoform X2 (*Cynara cardunculus* var. *scolymus*) |
| **68** | TRINITY_DN3057_c0_g1 | 2 | 34 | -4.409 | down | uncharacterized protein LOC112525788 isoform X1 (*Cynara cardunculus* var. *scolymus*) |
| **69** | TRINITY_DN8733_c0_g1 | 5 | 55 | -3.632 | down | ammonium transporter 3 member 1-like (*Lactuca sativa*) |
| **70** | TRINITY_DN17756_c4_g5 | 84 | 3567 | -5.464 | down | dihydroflavonol reductase (*Gynura bicolor*) |
| **71** | TRINITY_DN12959_c2_g2 | 199 | 6233 | -5.028 | down | UDP-Glycosyltransferase superfamily protein (*Artemisia annua*) |
| **72** | TRINITY_DN12959_c2_g1 | 93 | 2919 | -5.024 | down | crocetin glucosyltransferase, chloroplastic-like (*Cynara cardunculus* var. *scolymus*) |
| **73** | TRINITY_DN12959_c2_g3 | 10 | 270 | -4.867 | down | UDP-glycosyltransferase 75C1 (*Helianthus annuus*) |
| **74** | TRINITY_DN11487_c0_g1 | 187 | 10632 | -5.885 | down | chalcone synthase type 4 (*Dahlia pinnata*) |
| **75** | TRINITY_DN11108_c2_g1 | 3 | 94 | -5.030 | down | hypothetical protein E3N88_18335 (*Mikania micrantha*) |
| **76** | TRINITY_DN13405_c0_g1 | 7 | 78 | -3.622 | down | hypothetical protein E3N88_43878 (*Mikania micrantha*) |
| **77** | TRINITY_DN13846_c1_g1 | 10 | 179 | -4.278 | down | hydroxyisourate hydrolase-like (*Cucumis melo* var. *makuwa*) |
| **78** | TRINITY_DN13928_c3_g1 | 48 | 1364 | -4.895 | down | hydroxycinnamoyl-CoA quinate hydroxycinnamoyl-transferase (*Cynara cardunculus* var. *scolymus*) |
| **79** | TRINITY_DN18353_c1_g3 | 65 | 1764 | -4.817 | down | solute carrier family 35 member F1 isoform X4 (*Helianthus annuus*) |
| **80** | TRINITY_DN10242_c0_g5 | 166 | 7600 | -5.580 | down | 4-hydroxyphenylpyruvate dioxygenase (*Lactuca sativa*) |
| **81** | TRINITY_DN10701_c0_g2 | 258 | 8457 | -5.094 | down | malonyl-coenzyme A: anthocyanidin 3-O-glucoside-6''-O-malonyltransferase (*Pericallis cruenta*) |
| **82** | TRINITY_DN17842_c0_g1 | 265 | 6056 | -4.570 | down | UDP-glucose:flavonoid 3-O-glucosyltransferase (*Gynura bicolor*) |
| **83** | TRINITY_DN19492_c0_g2 | 36 | 2245 | -5.061 | down | anthocyanidin synthase (*Pericallis cruenta*) |
| **84** | TRINITY_DN18717_c1_g1 | 113 | 3260 | -4.909 | down | beta-glucosidase 11-like isoform X1 (*Vitis riparia*) |
| **85** | TRINITY_DN13077_c0_g2 | 29 | 682 | -4.617 | down | solute carrier family 35 member F1 isoform X4 (*Helianthus annuus*) |
| **86** | TRINITY_DN18717_c2_g3 | 8 | 182 | -4.512 | down | beta-glucosidase 22 isoform X1 (*Elaeis guineensis*) |
| **87** | TRINITY_DN18154_c0_g3 | 6 | 62 | -3.450 | down | uncharacterized protein LOC111890463 (*Lactuca sativa*) |
| **88** | TRINITY_DN19062_c0_g3 | 3 | 42 | -3.866 | down | hypothetical protein LSAT_3X43501 (*Lactuca sativa*) |
| **89** | TRINITY_DN13982_c1_g1 | 37 | 594 | -4.078 | down | solute carrier family 35 member F1-like isoform X1 (*Cynara cardunculus* var. *scolymus*) |
| **90** | TRINITY_DN9621_c1_g1 | 165 | 1932 | -3.550 | down | bHLH transcription factor 2 (*Chrysanthemum* × *morifolium*) |
| **91** | TRINITY_DN5035_c0_g1 | 0 | 9 | -5.737 | down | protein DETOXIFICATION 49-like (*Cynara cardunculus* var. *scolymus*) |
| **92** | TRINITY_DN13928_c3_g2 | 0 | 11 | -5.974 | down | hydroxycinnamoyl-CoA quinate/shikimate hydroxycinnamoyl transferase (*Cichorium intybus*) |
| **93** | TRINITY_DN11863_c0_g2 | 10 | 114 | -3.644 | down | unnamed protein product (*Lactuca saligna*) |
| **94** | TRINITY_DN13292_c3_g3 | 23 | 381 | -4.131 | down | glycosyl hydrolase 5 family protein (*Helianthus annuus*) |
| **95** | TRINITY_DN7883_c0_g2 | 3 | 66 | -4.725 | down | hypothetical protein CTI12_AA028920 (*Artemisia annua*) |
| **96** | TRINITY_DN19635_c4_g2 | 8 | 133 | -4.136 | down | hypothetical protein HanXRQr2_Chr15g0716461 (*Helianthus annuus*) |
| **97** | TRINITY_DN12959_c2_g4 | 48 | 2456 | -5.753 | down | crocetin glucosyltransferase, chloroplastic-like (*Cynara cardunculus* var. *scolymus*) |
| **98** | TRINITY_DN19492_c0_g1 | 20 | 1422 | -6.212 | down | leucoanthocyanidin dioxygenase (*Artemisia annua*) |
| **99** | TRINITY_DN10245_c2_g2 | 5 | 67 | -3.809 | down | hypothetical protein CTI12_AA535120 (*Artemisia annua*) |
| **100** | TRINITY_DN9214_c5_g1 | 9 | 86 | -3.326 | down | unnamed protein product (*Lactuca saligna*) |
| **101** | TRINITY_DN5338_c1_g1 | 12 | 127 | -3.486 | down | chitinase (*Artemisia annua*) |
| **102** | TRINITY_DN15133_c1_g1 | 464 | 13445 | -4.858 | down | flavanone 3-hydroxylase (*Gynura bicolor*) |
| **103** | TRINITY_DN7406_c0_g1 | 14 | 154 | -3.547 | down | protein RADIALIS-like 3 (*Cynara cardunculus* var. *scolymus*) |
| **104** | TRINITY_DN8906_c1_g5 | 7 | 292 | -5.538 | down | primary amine oxidase-like (*Lactuca sativa*) |
| **105** | TRINITY_DN10203_c2_g2 | 108 | 1029 | -3.335 | down | putative transcription factor ZF-HD family (*Helianthus annuus*) |
| **106** | TRINITY_DN9757_c1_g3 | 52 | 549 | -3.482 | down | putative fatty acid amide hydrolase (*Helianthus annuus*) |
| **107** | TRINITY_DN17856_c2_g6 | 3 | 42 | -3.880 | down | unnamed protein product  (*Helianthus annuus*) |
| **108** | TRINITY_DN8262_c1_g3 | 6 | 69 | -3.522 | down | hypothetical protein E3N88_30373 (*Mikania micrantha*) |
| **109** | TRINITY_DN5294_c0_g1 | 9 | 106 | -3.616 | down | protein DOG1-like 3 (*Lactuca sativa*) |
| **110** | TRINITY_DN16246_c1_g2 | 3 | 40 | -3.962 | down | receptor-like protein kinase-related family protein (*Artemisia annua*) |
| **111** | TRINITY_DN9277_c0_g2 | 62 | 725 | -3.603 | down | protein trichome birefringence-like 19 (*Helianthus annuus*) |
| **112** | TRINITY_DN9081_c1_g1 | 0.0 | 13 | -6.217 | down | 50S ribosomal protein L33-like (*Helianthus annuus*) |
| **113** | TRINITY_DN12787_c1_g5 | 0.0 | 16 | -6.503 | down | polynucleotidyl transferase, ribonuclease H-like superfamily protein (*Artemisia annua*) |
| **114** | TRINITY_DN7056_c0_g2 | 6 | 60 | -3.479 | down | high affinity nitrate transporter 2.5 (*Cynara cardunculus* var. *scolymus*) |
| **115** | TRINITY_DN6358_c0_g1 | 0.0 | 12 | -6.086 | down | guard cell S-type anion channel SLAC1 (*Lactuca sativa*) |
| **116** | TRINITY_DN9621_c1_g4 | 110 | 4748 | -5.493 | down | unnamed protein product (*Lactuca saligna*) |
| **117** | TRINITY_DN17410_c0_g2 | 0.0 | 10 | -5.886 | down | hypothetical protein E3N88_21025 (*Mikania micrantha*) |
| **118** | TRINITY_DN6698_c0_g1 | 0.0 | 11 | -6.017 | down | non-specific lipid-transfer protein-like protein At2g13820 (*Cynara cardunculus* var. *scolymus*) |
| **119** | TRINITY_DN4749_c0_g1 | 0.0 | 7 | -5.391 | down | acid phosphatase, class B-like protein (*Artemisia annua*) |
| **120** | TRINITY_DN10742_c2_g4 | 0.0 | 25 | -7.135 | down | expansin-A10 (*Helianthus annuus*) |
| **121** | TRINITY_DN16253_c0_g4 | 0.0 | 12 | -6.131 | down | CASP-like protein 1F1 (*Cynara cardunculus* var. *scolymus*) |
| **122** | TRINITY_DN3850_c0_g1 | 0.0 | 8 | -5.507 | down | hypothetical protein CTI12_AA293710 (*Artemisia annua*) |
| **123** | TRINITY_DN16951_c1_g3 | 0.0 | 9 | -5.679 | down | Zinc finger, Dof-type (*Artemisia annua*) |
| **124** | TRINITY_DN14576_c1_g2 | 0.0 | 9 | -5.616 | down | uncharacterized protein LOC110928405 (*Helianthus annuus*) |
| **125** | TRINITY_DN5035_c0_g4 | 0.0 | 9 | -5.673 | down | Multi antimicrobial extrusion protein (*Artemisia annua*) |
| **126** | TRINITY_DN6358_c0_g2 | 0.0 | 15 | -6.380 | down | C4-dicarboxylate transporter/malic acid transport protein (*Artemisia annua*) |
| **127** | TRINITY_DN9703_c3_g1 | 0.0 | 16 | -6.521 | down | transcription factor IBH1-like (*Cynara cardunculus* var. *scolymus*) |
| **128** | TRINITY_DN829_c0_g1 | 0.0 | 42 | -7.898 | down | lysine histidine transporter-like 8 (*Cynara cardunculus* var. *scolymus*) |
| **129** | TRINITY_DN11860_c1_g1 | 3 | 56 | -4.132 | down | NB-ARC domains-containing protein (*Artemisia annua*) |
| **130** | TRINITY_DN636_c0_g1 | 0.0 | 16 | -6.495 | down | RING/U-box superfamily protein (*Artemisia annua*) |
| **131** | TRINITY_DN14795_c0_g2 | 2 | 22 | -3.499 | down | hypothetical protein E3N88_40521 (*Mikania micrantha*) |
| **132** | TRINITY_DN6093_c0_g1 | 0.0 | 18 | -6.645 | down | unnamed protein product (*Lactuca saligna*) |
| **133** | TRINITY_DN14666_c1_g1 | 55 | 554 | -3.402 | down | CASP-like protein 1F1 (*Cynara cardunculus* var. *scolymus*) |
| **134** | TRINITY_DN14029_c0_g1 | 9 | 350 | -5.285 | down | zeatin O-glucosyltransferase-like (*Lactuca sativa*) |
| **135** | TRINITY_DN18335_c0_g3 | 0.0 | 9 | -5.606 | down | LETM1-like protein (*Artemisia annua*) |
| **136** | TRINITY_DN9033_c1_g3 | 0.0 | 32 | -7.483 | down | Protein of unknown function DUF617, plant (*Cynara cardunculus* var. *scolymus*) |
| **137** | TRINITY_DN11335_c4_g2 | 121 | 2218 | -4.236 | down | hypothetical protein E3N88_05074 (*Mikania micrantha*) |
| **138** | TRINITY_DN9835_c0_g7 | 1 | 16 | -3.670 | down | putative leucine-rich repeat-containing, plant-type, leucine-rich repeat domain superfamily (*Helianthus annuus*) |
| **139** | TRINITY_DN4487_c0_g1 | 0.0 | 20 | -6.785 | down | RING-H2 finger protein ATL70 (*Helianthus annuus*) |
| **140** | TRINITY_DN15108_c2_g2 | 0.0 | 11 | -5.946 | down | PREDICTED: NAC transcription factor 25 (*Nicotiana sylvestris*) |
| **141** | TRINITY_DN10480_c2_g2 | 0.0 | 27 | -7.256 | down | protein PELOTA 1 isoform X2 (*Cynara cardunculus* var. *scolymus*) |
| **142** | TRINITY_DN21675_c0_g1 | 0.0 | 44 | -7.937 | down | stigma-specific STIG1-like protein 1 (*Helianthus annuus*) |
| **143** | TRINITY_DN9482_c0_g1 | 0.0 | 40 | -7.790 | down | Uncharacterized protein family UPF0220 (*Cynara cardunculus* var. *scolymus*) |
| **144** | TRINITY_DN14392_c1_g2 | 0.0 | 33 | -7.540 | down | hypothetical protein CTI12_AA619560 (*Artemisia annua*) |
| **145** | TRINITY_DN18848_c0_g1 | 0.0 | 61 | -8.404 | down | hypothetical protein E3N88_42881 (*Mikania micrantha*) |
| **146** | TRINITY_DN2210_c0_g1 | 0.0 | 25 | -7.101 | down | hypothetical protein CTI12_AA052670 (*Artemisia annua*) |
| **147** | TRINITY_DN9123_c0_g7 | 0.0 | 13 | -6.217 | down | hypothetical protein CTI12_AA343190 (*Artemisia annua*) |
| **148** | TRINITY_DN16127_c1_g6 | 0.0 | 27 | -7.252 | down | uncharacterized protein LOC111913972 (*Lactuca sativa*) |
| **149** | TRINITY_DN319_c0_g1 | 0.0 | 25 | -7.099 | down | probable receptor-like protein kinase At1g49730 (*Helianthus annuus*) |
| **150** | TRINITY_DN14334_c0_g2 | 0.0 | 31 | -7.414 | down | uncharacterized protein LOC112502092 (*Cynara cardunculus* var. *scolymus*) |
| **151** | TRINITY_DN5320_c0_g1 | 0.0 | 14 | -6.315 | down | glu S.griseus protease inhibitor (*Helianthus annuus*) |
| **152** | TRINITY_DN13652_c3_g2 | 181 | 1843 | -3.401 | down | expansin-like A1 (*Artemisia annua*) |
| **153** | TRINITY_DN11254_c1_g1 | 10 | 155 | -3.979 | down | plasma membrane ATPase 1 (*Helianthus annuus*) |
| **154** | TRINITY_DN4990_c0_g2 | 7 | 68 | -3.356 | down | uncharacterized protein LOC110912556 (*Helianthus annuus*) |
| **155** | TRINITY_DN22587_c0_g1 | 3 | 62 | -4.439 | down | anthocyanidin 3-O-glucosyltransferase 5-like (*Cynara cardunculus* var. *scolymus*) |
| **156** | TRINITY_DN9464_c3_g2 | 1 | 29 | -4.934 | down | hypothetical protein L484_006110 (*Morus notabilis*) |
| **157** | TRINITY_DN19504_c3_g6 | 2 | 20 | -3.658 | down | BTB/POZ and TAZ domain-containing protein 1-like (*Lactuca sativa*) |
| **158** | TRINITY_DN6324_c0_g1 | 3 | 29 | -3.521 | down | uncharacterized protein LOC112523565 (*Cynara cardunculus* var. *scolymus*) |
| **159** | TRINITY_DN11372_c1_g1 | 2 | 32 | -3.841 | down | monothiol glutaredoxin-S10 (*Lactuca sativa*) |
| **160** | TRINITY_DN13383_c0_g3 | 5 | 83 | -4.125 | down | probable carotenoid cleavage dioxygenase 4, chloroplastic (*Lactuca sativa*) |
| **161** | TRINITY_DN21047_c0_g1 | 3 | 28 | -3.480 | down | uncharacterized GPI-anchored protein At3g06035 (*Helianthus annuus*) |
| **162** | TRINITY_DN12150_c1_g2 | 4 | 41 | -3.547 | down | serine hydrolase FSH (*Artemisia annua*) |
| **163** | TRINITY_DN5339_c0_g2 | 1 | 20 | -3.941 | down | unnamed protein product (*Lactuca saligna*) |
| **164** | TRINITY_DN5710_c0_g1 | 2 | 21 | -3.445 | down | unnamed protein product (*Lactuca saligna*) |
| **165** | TRINITY_DN8059_c0_g1 | 5 | 51 | -3.536 | down | vegetative storage protein, VSP (*Artemisia annua*) |
| **166** | TRINITY_DN15108_c2_g3 | 0.7 | 33 | -5.697 | down | unnamed protein product (*Lactuca saligna*) |
| **167** | TRINITY_DN7056_c0_g1 | 2 | 21 | -3.764 | down | nitrate transporter 2.5, partial (*Chrysanthemum* × *morifolium*) |
| **168** | TRINITY_DN7216_c0_g1 | 6 | 68 | -3.600 | down | late embryogenesis abundant (LEA) hydroxyproline-rich glycoprotein family (*Artemisia annua*) |
| **169** | TRINITY_DN18251_c0_g1 | 3 | 39 | -3.765 | down | leucine-rich repeat domain, L domain-like protein (*Artemisia annua*) |
| **170** | TRINITY_DN12998_c1_g1 | 2 | 32 | -4.312 | down | hypothetical protein E3N88_42913 (*Mikania micrantha*) |
| **171** | TRINITY_DN18722_c1_g1 | 2 | 32 | -4.312 | down | hypothetical protein E3N88_06793 (*Mikania micrantha*) |
| **172** | TRINITY_DN16563_c0_g2 | 1 | 66 | -5.709 | down | sugar transporter ERD6-like 7 (*Helianthus annuus*) |
| **173** | TRINITY_DN14649_c1_g3 | 1 | 51 | -5.321 | down | putative nucleotidyltransferase, Ribonuclease H (*Helianthus annuus*) |
| **174** | TRINITY_DN5441_c0_g1 | 0.7 | 26 | -5.367 | down | small auxin-up RNA (*Artemisia annua*) |
| **175** | TRINITY_DN17432_c2_g2 | 1 | 32 | -5.057 | down | hypothetical protein E3N88_42168 (*Mikania micrantha*) |
| **176** | TRINITY_DN6435_c0_g1 | 1 | 30 | -4.974 | down | hypothetical protein CTI12_AA055170 (*Artemisia annua*) |
| **177** | TRINITY_DN1404_c0_g1 | 1 | 40 | -4.985 | down | START-like domain-containing protein (*Artemisia annua*) |
| **178** | TRINITY_DN6522_c0_g1 | 2 | 24 | -3.961 | down | G-type lectin S-receptor-like serine/threonine-protein kinase At5g35370 (*Helianthus annuus*) |
| **179** | TRINITY_DN4466_c0_g1 | 0.7 | 22 | -5.106 | down | hypothetical protein E3N88_18982 (*Mikania micrantha*) |
| **180** | TRINITY_DN18601_c1_g6 | 1 | 14 | -3.444 | down | cysteine-rich receptor-like protein kinase 2 (*Helianthus annuus*) |
| **181** | TRINITY_DN9500_c2_g1 | 1 | 16 | -4.075 | down | unnamed protein product (*Lactuca saligna*) |
| **182** | TRINITY_DN3504_c0_g1 | 2 | 24 | -3.926 | down | uncharacterized protein LOC111903592 (*Lactuca sativa*) |
| **183** | TRINITY_DN7883_c0_g1 | 3 | 60 | -4.252 | down | hypothetical protein CTI12_AA028920 (*Artemisia annua*) |
| **184** | TRINITY_DN7216_c0_g2 | 2 | 20 | -3.425 | down | late embryogenesis abundant (LEA) hydroxyproline-rich glycoprotein family (*Artemisia annua*) |
| **185** | TRINITY_DN8262_c1_g1 | 7 | 78 | -3.623 | down | hypothetical protein E3N88_07486 (*Mikania micrantha*) |
| **186** | TRINITY_DN9151_c1_g1 | 3 | 119 | -5.228 | down | hypothetical protein MIMGU_mgv1a017316mg (*Erythranthe guttata*) |
| **187** | TRINITY_DN16134_c0_g1 | 6 | 121 | -4.354 | down | cytochrome P450 (*Artemisia annua*) |
| **188** | TRINITY_DN13493_c0_g4 | 3 | 39 | -3.620 | down | very-long-chain 3-ketoacyl-CoA synthase, Thiolase-like protein (*Artemisia annua*) |
| **189** | TRINITY_DN16629_c1_g4 | 18 | 181 | -3.382 | down | uncharacterized protein LOC110926659 (*Helianthus annuus*) |
| **190** | TRINITY_DN7469_c0_g1 | 3 | 38 | -3.911 | down | unnamed protein product (*Lactuca saligna*) |
| **191** | TRINITY_DN5506_c0_g1 | 1 | 39 | -4.967 | down | Barwin-like endoglucanase (*Cynara cardunculus* var. *scolymus*) |
| **192** | TRINITY_DN10295_c1_g1 | 143 | 1630 | -3.616 | down | pectinesterase 2 (*Helianthus annuus*) |
| **193** | TRINITY_DN6959_c0_g1 | 1 | 58 | -5.871 | down | putative EH domain, EF-hand domain pair protein (*Helianthus annuus*) |
| **194** | TRINITY_DN11608_c0_g2 | 0.7 | 14 | -4.423 | down | heme peroxidase (*Artemisia annua*) |
| **195** | TRINITY_DN10852_c1_g4 | 2 | 41 | -4.692 | down | hypothetical protein LSAT_9X55841 (*Lactuca sativa*) |
| **196** | TRINITY_DN5386_c0_g1 | 4 | 48 | -3.547 | down | ammonium transporter 3 member 1-like (*Lactuca sativa*) |
| **197** | TRINITY_DN19604_c2_g3 | 32 | 374 | -3.604 | down | unnamed protein product (*Lactuca saligna*) |
| **198** | TRINITY_DN19604_c2_g1 | 14 | 154 | -3.495 | down | PREDICTED: uncharacterized protein LOC104752275 (*Camelina sativa*) |
| **199** | TRINITY_DN11052_c3_g2 | 9 | 160 | -4.167 | down | unnamed protein product (*Microthlaspi erraticum*) |
| **200** | TRINITY_DN10742_c2_g5 | 34 | 635 | -4.301 | down | Barwin-like endoglucanase (*Artemisia annua*) |
| **201** | TRINITY_DN11762_c5_g1 | 3 | 106 | -5.217 | down | MLO-like protein 12 (*Cynara cardunculus* var. *scolymus*) |
| **202** | TRINITY_DN6367_c0_g1 | 13 | 153 | -3.659 | down | potassium transporter (*Artemisia annua*) |
| **203** | TRINITY_DN9332_c0_g10 | 14 | 156 | -3.539 | down | beta-lactamase domain-containing protein 2-like (*Lactuca sativa*) |
| **204** | TRINITY_DN6995_c0_g1 | 4 | 63 | -4.010 | down | major intrinsic protein, Aquaporin-like protein (*Artemisia annua*) |
| **205** | TRINITY_DN18717_c2_g4 | 2 | 55 | -4.830 | down | hypothetical protein BHE74_00037478 (*Ensete ventricosum*) |
| **206** | TRINITY_DN14333_c1_g1 | 6 | 153 | -4.693 | down | 3-beta hydroxysteroid dehydrogenase/isomerase (*Cynara cardunculus* var. *scolymus*) |
| **207** | TRINITY_DN13210_c1_g1 | 105 | 2054 | -4.336 | down | basic helix-loop-helix protein A (*Cynara cardunculus* var. *scolymus*) |
| **208** | TRINITY_DN13846_c1_g3 | 295 | 5272 | -4.205 | down | unnamed protein product (*Lactuca saligna*) |
| **209** | TRINITY_DN11108_c2_g3 | 159 | 3762 | -4.606 | down | peptidase S10, serine carboxypeptidase, Alpha/Beta hydrolase fold protein (*Artemisia annua*) |
| **210** | TRINITY_DN18717_c2_g1 | 477 | 9831 | -4.409 | down | beta-glucosidase 11-like (*Malus domestica*) |
| **211** | TRINITY_DN11108_c2_g2 | 44 | 995 | -4.538 | down | peptidase S10, serine carboxypeptidase, Alpha/Beta hydrolase fold protein (*Artemisia annua*) |
| **212** | TRINITY_DN12893_c1_g1 | 4 | 55 | -3.726 | down | probable polygalacturonase (*Helianthus annuus*) |
| **213** | TRINITY_DN7816_c0_g1 | 42 | 4 | 3.434 | up | homeobox-leucine zipper protein GLABRA 2 isoform X1 (*Cynara cardunculus* var. *scolymus*) |
| **214** | TRINITY_DN4915_c0_g1 | 32 | 2 | 3.681 | up | auxin-induced protein 22A (*Artemisia annua*) |
| **215** | TRINITY_DN17042_c0_g3 | 444 | 18 | 4.506 | up | hypothetical protein E3N88_05002 (*Mikania micrantha*) |
| **216** | TRINITY_DN11038_c1_g1 | 118 | 3 | 5.392 | up | hypothetical protein LSAT_5X130221 (*Lactuca sativa*) |
| **217** | TRINITY_DN11936_c2_g1 | 434 | 37 | 3.484 | up | GDSL esterase/lipase At1g71691-like (*Cynara cardunculus* var. *scolymus*) |
| **218** | TRINITY_DN10998_c0_g2 | 11 | 0 | 5.854 | up | protein WVD2-like 5 (*Cynara cardunculus* var. *scolymus*) |
| **219** | TRINITY_DN15017_c1_g3 | 13 | 0 | 6.077 | up | acyl-protein thioesterase 1 homolog 1-like (*Cynara cardunculus* var. *scolymus*) |
| **220** | TRINITY_DN19576_c0_g1 | 11 | 0 | 5.836 | up | G2/mitotic-specific cyclin-2 (*Cynara cardunculus* var. *scolymus*) |
| **221** | TRINITY_DN10268_c1_g2 | 9 | 0 | 5.501 | up | ethylene-responsive transcription factor ERF038-like, partial (*Cynara cardunculus* var. *scolymus*) |
| **222** | TRINITY_DN12978_c2_g4 | 10 | 0 | 5.782 | up | unnamed protein product (*Lactuca saligna*) |
| **223** | TRINITY_DN17423_c4_g6 | 10 | 0 | 5.759 | up | mitotic spindle checkpoint protein BUBR1-like (*Cynara cardunculus* var. *scolymus*) |
| **224** | TRINITY_DN3256_c0_g1 | 21 | 1 | 4.368 | up | putative transcription factor C2H2 family (*Helianthus annuus*) |
| **225** | TRINITY_DN13664_c0_g2 | 11 | 0 | 5.882 | up | phosphoinositide phospholipase C 4-like (*Cynara cardunculus* var. *scolymus*) |
| **226** | TRINITY_DN9890_c0_g1 | 30 | 0 | 7.368 | up | SGNH hydrolase-type esterase domain-containing protein (*Artemisia annua*) |
| **227** | TRINITY_DN14951_c0_g2 | 1546 | 75 | 4.310 | up | beta-fructofuranosidase, soluble isoenzyme I-like (*Cynara cardunculus* var. *scolymus*) |
| **228** | TRINITY_DN10705_c0_g3 | 51 | 4 | 3.506 | up | hypothetical protein E3N88_21016 (*Mikania micrantha*) |
| **229** | TRINITY_DN15869_c2_g3 | 46 | 3 | 3.880 | up | uncharacterized protein LOC110879139 (*Helianthus annuus*) |
| **230** | TRINITY_DN13012_c0_g1 | 119 | 4 | 4.730 | up | homeobox-leucine zipper protein HDG5 (*Helianthus annuus*) |
| **231** | TRINITY_DN10796_c1_g3 | 62 | 4 | 3.922 | up | GRF1-interacting factor 1-like isoform X1 (*Lactuca sativa*) |
| **232** | TRINITY_DN9178_c0_g1 | 88 | 8 | 3.407 | up | galactose oxidase/kelch, beta-propeller (*Artemisia annua*) |
| **233** | TRINITY_DN9233_c1_g1 | 206 | 14 | 3.813 | up | beta-fructofuranosidase, soluble isoenzyme I (*Helianthus annuus*) |
| **234** | TRINITY_DN11335_c1_g1 | 25 | 0 | 6.992 | up | unnamed protein product (*Lactuca saligna*) |
| **235** | TRINITY_DN16587_c0_g3 | 22 | 0 | 6.835 | up | transcription factor ORG2-like (*Cynara cardunculus* var. *scolymus*) |
| **236** | TRINITY_DN10959_c0_g3 | 1725 | 0 | 13.100 | up | stigma-specific peroxidase precursor (*Senecio squalidus*) |
| **237** | TRINITY_DN9461_c0_g1 | 407 | 0 | 11.006 | up | unnamed protein product (*Lactuca saligna*) |
| **238** | TRINITY_DN19483_c0_g2 | 21 | 0 | 6.768 | up | protein SHOOT GRAVITROPISM 5-like (*Cynara cardunculus* var. *scolymus*) |
| **239** | TRINITY_DN13240_c1_g1 | 330 | 0 | 10.711 | up | mavicyanin (*Helianthus annuus*) |
| **240** | TRINITY_DN4801_c0_g2 | 18 | 0 | 6.545 | up | hypothetical protein CTI12_AA030440 (*Artemisia annua*) |
| **241** | TRINITY_DN8297_c0_g1 | 658 | 0 | 11.709 | up | cytochrome P450 (*Artemisia annua*) |
| **242** | TRINITY_DN3464_c0_g1 | 19 | 0 | 6.644 | up | proton pump-interactor 1-like (*Cynara cardunculus* var. *scolymus*) |
| **243** | TRINITY_DN7607_c0_g1 | 25 | 0 | 7.025 | up | 21 kDa protein (*Helianthus annuus*) |
| **244** | TRINITY_DN9759_c1_g4 | 20 | 0 | 6.649 | up | E-beta-Farnesene synthase 1, partial (*Tanacetum cinerariifolium*) |
| **245** | TRINITY_DN8635_c0_g1 | 52 | 0 | 8.032 | up | protein trichome birefringence-like 34 (*Lactuca sativa*) |
| **246** | TRINITY_DN9626_c0_g2 | 12 | 0 | 5.962 | up | hypothetical protein LSAT_9X49640 (*Lactuca sativa*) |
| **247** | TRINITY_DN15047_c1_g1 | 422 | 28 | 3.828 | up | GDSL esterase/lipase At5g33370 (*Helianthus annuus*) |
| **248** | TRINITY_DN9461_c0_g2 | 600 | 1 | 9.117 | up | cupredoxin, Blue (type 1) copper protein, binding site (*Artemisia annua*) |
| **249** | TRINITY_DN21042_c0_g1 | 18 | 2 | 3.376 | up | PawS-like protein 1b (*Jacobaea abrotanifolia*) |
| **250** | TRINITY_DN6252_c0_g1 | 45 | 2 | 4.668 | up | hypothetical protein E3N88_21187 (*Mikania micrantha*) |
| **251** | TRINITY_DN13203_c0_g2 | 294 | 21 | 3.733 | up | unnamed protein product (*Lactuca saligna*) |
| **252** | TRINITY_DN9152_c0_g1 | 394 | 5 | 6.201 | up | F-box/LRR-repeat protein At3g59190-like isoform X1 (*Cynara cardunculus* var. *scolymus*) |
| **253** | TRINITY_DN16476_c0_g1 | 50 | 4 | 3.545 | up | unnamed protein product (*Lactuca saligna*) |
| **254** | TRINITY_DN7073_c0_g1 | 66 | 3 | 4.191 | up | steroid 5-alpha-reductase DET2 (*Helianthus annuus*) |
| **255** | TRINITY_DN10166_c1_g2 | 53 | 3 | 3.935 | up | ribonuclease H-like domain-containing protein (*Artemisia annua*) |
| **256** | TRINITY_DN18223_c2_g2 | 192 | 13 | 3.850 | up | protein IQ-DOMAIN 1 (*Helianthus annuus*) |
| **257** | TRINITY_DN12381_c0_g6 | 107 | 5 | 4.309 | up | PREDICTED: probable 18S rRNA (guanine-N(7))-methyltransferase (*Vitis vinifera*) |
| **258** | TRINITY_DN13740_c2_g1 | 666 | 21 | 4.955 | up | Retrovirus-related Pol polyprotein from transposon TNT 1-94 (*Cajanus cajan*) |
| **259** | TRINITY_DN18458_c1_g1 | 254 | 14 | 4.089 | up | Double-stranded RNA-binding (*Cynara cardunculus* var. *scolymus*) |
| **260** | TRINITY_DN17034_c0_g1 | 166 | 8 | 4.401 | up | glycerophosphodiester phosphodiesterase GDPDL3-like (*Cynara cardunculus* var. *scolymus*) |
| **261** | TRINITY_DN8423_c0_g1 | 52 | 3 | 4.234 | up | hypothetical protein CTI12_AA471830 (*Artemisia annua*) |
| **262** | TRINITY_DN11032_c1_g2 | 88 | 4 | 4.342 | up | dynein light chain LC6, flagellar outer arm (*Helianthus annuus*) |
| **263** | TRINITY_DN12994_c0_g2 | 29 | 1 | 4.839 | up | unnamed protein product (*Prunus armeniaca*) |
| **264** | TRINITY_DN11284_c0_g1 | 2289 | 55 | 5.378 | up | hypothetical protein E3N88_20623 (*Mikania micrantha*) |
| **265** | TRINITY_DN9129_c0_g6 | 97 | 8 | 3.518 | up | histone H2A.1-like (*Cynara cardunculus* var. *scolymus*) |
| **266** | TRINITY_DN10242_c0_g2 | 870 | 53 | 3.925 | up | 4-hydroxyphenylpyruvate dioxygenase (*Lactuca sativa*) |
| **267** | TRINITY_DN12099_c0_g5 | 26 | 2 | 3.610 | up | triose phosphate/phosphate translocator, non-green plastid, chloroplastic-like (*Olea europaea* var. *sylvestris*) |
| **268** | TRINITY_DN10547_c0_g1 | 67 | 5 | 3.809 | up | protein PLASTID TRANSCRIPTIONALLY ACTIVE 10 isoform X1 (*Lactuca sativa*) |
| **269** | TRINITY_DN11764_c1_g2 | 122 | 4 | 4.755 | up | unnamed protein product (*Lactuca saligna*) |
| **270** | TRINITY_DN8279_c1_g2 | 32 | 2 | 3.877 | up | truncated transcription factor CAULIFLOWER A-like (*Cynara cardunculus* var. *scolymus*) |
| **271** | TRINITY_DN18165_c2_g3 | 570 | 1 | 8.645 | up | uncharacterized protein LOC112524742 (*Cynara cardunculus* var. *scolymus*) |
| **272** | TRINITY_DN11585_c2_g2 | 89 | 3 | 4.997 | up | linoleate 13S-lipoxygenase 2-1, chloroplastic-like (*Cynara cardunculus* var. *scolymus*) |
| **273** | TRINITY_DN12532_c0_g3 | 22 | 1 | 4.996 | up | RecName: Full=Lipoxygenase 2, chloroplastic; Short=TcLOX2; Flags: Precursor (*Tanacetum cinerariifolium*) |
| **274** | TRINITY_DN10453_c1_g2 | 43 | 3 | 3.994 | up | G2/mitotic-specific cyclin C13-1-like (*Cynara cardunculus* var. *scolymus*) |
| **275** | TRINITY_DN11636_c0_g1 | 50 | 2 | 4.352 | up | UDP-glycosyltransferase 85C1 isoform X1 (*Helianthus annuus*) |
| **276** | TRINITY_DN14537_c1_g1 | 20 | 1 | 3.826 | up | root phototropism protein 3-like (*Cynara cardunculus* var. *scolymus*) |
| **277** | TRINITY_DN17291_c0_g1 | 17 | 1 | 3.990 | up | hypothetical protein E3N88_39489 (*Mikania micrantha*) |
| **278** | TRINITY_DN3053_c0_g1 | 61 | 1 | 5.471 | up | UPF0553 protein (*Artemisia annua*) |
| **279** | TRINITY_DN19780_c1_g1 | 185 | 3 | 5.872 | up | wall-associated receptor kinase 2 isoform X1 (*Helianthus annuus*) |
| **280** | TRINITY_DN19726_c8_g2 | 24 | 2 | 3.784 | up | uncharacterized protein LOC112501480 (*Cynara cardunculus* var. *scolymus*) |
| **281** | TRINITY_DN6375_c0_g2 | 17 | 1 | 3.657 | up | Pathogenesis-related protein PR-1 precursor (*Artemisia argyi*) |
| **282** | TRINITY_DN10499_c3_g1 | 47 | 4 | 3.407 | up | protein CUP-SHAPED COTYLEDON 2 (*Helianthus annuus*) |
| **283** | TRINITY_DN14665_c2_g2 | 14 | 1 | 3.397 | up | monocopper oxidase-like protein SKU5 (*Cynara cardunculus* var. *scolymus*) |
| **284** | TRINITY_DN13886_c0_g2 | 22 | 1 | 4.050 | up | protein MIZU-KUSSEI 1-like (*Cynara cardunculus* var. *scolymus*) |
| **285** | TRINITY_DN11223_c1_g1 | 88 | 2 | 5.231 | up | putative glycosyltransferase 7 (*Helianthus annuus*) |
| **286** | TRINITY_DN14923_c0_g3 | 18 | 1 | 4.121 | up | tRNA-dihydrouridine(16/17) synthase (*NAD(P)(+)*)-like (*Cynara cardunculus* var. *scolymus*) |
| **287** | TRINITY_DN19695_c2_g1 | 38 | 2 | 4.214 | up | NB-ARC domain-containing disease resistance protein (*Prunus dulcis*) |
| **288** | TRINITY_DN13045_c0_g5 | 49 | 2 | 4.321 | up | hypothetical protein E3N88_24035 (*Mikania micrantha*) |
| **289** | TRINITY_DN12926_c4_g2 | 84 | 1 | 6.370 | up | cytochrome P450 71A4-like (*Cynara cardunculus* var. *scolymus*) |
| **290** | TRINITY_DN14648_c0_g4 | 57 | 1 | 5.415 | up | F-box/kelch-repeat protein At3g06240 (*Helianthus annuus*) |
| **291** | TRINITY_DN9688_c2_g2 | 26 | 1 | 4.715 | up | hypothetical protein CTI12_AA181850 (*Artemisia annua*) |
| **292** | TRINITY_DN13823_c0_g3 | 25 | 2 | 3.941 | up | uncharacterized protein LOC112524241 (*Cynara cardunculus* var. *scolymus*) |
| **293** | TRINITY_DN14511_c1_g5 | 185 | 12 | 3.822 | up | C2 domain, Synaptotagmin-like mitochondrial-lipid-binding domain-containing protein (*Artemisia annua*) |
| **294** | TRINITY_DN12716_c0_g1 | 165 | 6 | 4.699 | up | hypothetical protein E3N88_27303 (*Mikania micrantha*) |
| **295** | TRINITY_DN9703_c2_g1 | 69 | 0 | 7.490 | up | unnamed protein product (*Lactuca saligna*) |
| **296** | TRINITY_DN11389_c1_g1 | 552 | 27 | 4.296 | up | beta-fructofuranosidase, soluble isoenzyme I (*Helianthus annuus*) |
| **297** | TRINITY_DN8845_c0_g2 | 87 | 3 | 4.788 | up | uncharacterized protein LOC110937549 (*Helianthus annuus*) |
| **298** | TRINITY_DN15937_c1_g1 | 173 | 15 | 3.469 | up | uncharacterized protein LOC112523389 isoform X2 (*Cynara cardunculus* var. *scolymus*) |
| **299** | TRINITY_DN10775_c1_g1 | 182 | 12 | 3.921 | up | hypothetical protein E3N88_19761 (*Mikania micrantha*) |
| **300** | TRINITY_DN4805_c0_g1 | 16 | 1 | 4.623 | up | hypothetical protein CTI12_AA553230 (*Artemisia annua*) |
| **301** | TRINITY_DN16093_c2_g1 | 18 | 0 | 5.638 | up | putative serine carboxypeptidase-like 23 (*Helianthus annuus*) |
| **302** | TRINITY_DN17290_c1_g3 | 13 | 0 | 5.137 | up | uncharacterized protein LOC109830820 (*Asparagus officinalis*) |
| **303** | TRINITY_DN13681_c3_g1 | 8698 | 785 | 3.467 | up | miraculin-like (*Lactuca sativa*) |
| **304** | TRINITY_DN10627_c1_g2 | 150 | 10 | 3.893 | up | glycosylphosphatidylinositol-anchored lipid protein transfer 1 (*Artemisia annua*) |
| **305** | TRINITY_DN17905_c0_g1 | 190 | 17 | 3.457 | up | alanine aminotransferase 2-like (*Cynara cardunculus* var. *scolymus*) |
| **306** | TRINITY_DN7950_c0_g1 | 227 | 17 | 3.676 | up | cation exchanger 3 (*Artemisia annua*) |
| **307** | TRINITY_DN10920_c3_g2 | 18 | 0 | 5.603 | up | SHI-related sequence 5 (*Artemisia annua*) |
| **308** | TRINITY_DN8103_c0_g3 | 28 | 0 | 6.215 | up | hypothetical protein E3N88_20909 (*Mikania micrantha*) |
| **309** | TRINITY_DN5101_c0_g2 | 24 | 0 | 6.009 | up | nucleotide-binding alpha-beta plait domain-containing protein (*Artemisia annua*) |
| **310** | TRINITY_DN19483_c0_g1 | 12 | 0 | 4.978 | up | SSXT-like protein (*Cynara cardunculus* var. *scolymus*) |
| **311** | TRINITY_DN7921_c0_g1 | 14 | 0 | 5.227 | up | zinc finger protein WIP2-like (*Lactuca sativa*) |
| **312** | TRINITY_DN17927_c0_g1 | 19 | 0 | 5.735 | up | uncharacterized protein LOC110906459 (*Helianthus annuus*) |
| **313** | TRINITY_DN5595_c0_g1 | 15 | 0 | 5.324 | up | hypothetical protein CTI12_AA355210 (*Artemisia annua*) |
| **314** | TRINITY_DN21582_c0_g1 | 44 | 0 | 6.947 | up | proteinase inhibitor, propeptide (*Artemisia annua*) |
| **315** | TRINITY_DN4956_c0_g1 | 40 | 1 | 5.300 | up | F-box protein At1g67340 (*Helianthus annuus*) |
| **316** | TRINITY_DN15801_c1_g2 | 153 | 8 | 4.229 | up | ferric reduction oxidase 8 (*Artemisia annua*) |
| **317** | TRINITY_DN19104_c0_g4 | 39 | 4 | 3.348 | up | acyl-CoA N-acyltransferase (*Artemisia annua*) |
| **318** | TRINITY_DN20481_c0_g1 | 62 | 3 | 4.476 | up | peptide-N4-(N-acetyl-beta-glucosaminyl)asparagine amidase A-like (*Cynara cardunculus* var. *scolymus*) |
| **319** | TRINITY_DN8945_c1_g3 | 69 | 3 | 4.465 | up | BURP domain protein RD22-like (*Lactuca sativa*) |
| **320** | TRINITY_DN9661_c0_g2 | 113 | 5 | 4.541 | up | double-stranded RNA-binding protein 4 isoform X2 (*Helianthus annuus*) |
| **321** | TRINITY_DN13424_c1_g4 | 22 | 0 | 5.842 | up | hypothetical protein E3N88_39963 (*Mikania micrantha*) |
| **322** | TRINITY_DN10306_c0_g1 | 67 | 2 | 4.981 | up | 3-ketoacyl-CoA synthase 5 (*Helianthus annuus*) |
| **323** | TRINITY_DN15945_c0_g2 | 21 | 1 | 4.929 | up | hypothetical protein LSAT_3X36141 (*Lactuca sativa*) |
| **324** | TRINITY_DN6475_c0_g1 | 18 | 1 | 4.682 | up | AAA+ ATPase domain-containing protein (*Cynara cardunculus* var. *scolymus*) |
| **325** | TRINITY_DN5564_c0_g1 | 17 | 1 | 4.624 | up | triacylglycerol lipase 2-like (*Cynara cardunculus* var. *scolymus*) |
| **326** | TRINITY_DN9329_c1_g5 | 10 | 0 | 4.768 | up | hypothetical protein HanXRQr2_Chr15g0699501 (*Helianthus annuus*) |
| **327** | TRINITY_DN10974_c0_g1 | 16 | 1 | 4.583 | up | DnaJ domain-containing protein (*Cynara cardunculus* var. *scolymus*) |
| **328** | TRINITY_DN17211_c0_g2 | 14 | 0 | 5.240 | up | unnamed protein product, partial (*Vitis vinifera*) |
| **329** | TRINITY_DN14174_c1_g1 | 34 | 1 | 5.649 | up | Retrovirus-related Pol polyprotein from transposon RE1 (*Vitis vinifera*) |
| **330** | TRINITY_DN15748_c0_g1 | 22 | 1 | 5.002 | up | Ankyrin repeat-containing protein (*Artemisia annua*) |
| **331** | TRINITY_DN14525_c0_g5 | 133 | 2 | 5.814 | up | Retrovirus-related Pol polyprotein from transposon TNT 1-94 (*Vitis vinifera*) |
| **332** | TRINITY_DN12808_c3_g3 | 36 | 1 | 5.155 | up | hypothetical protein CTI12_AA560990 (*Artemisia annua*) |
| **333** | TRINITY_DN8611_c1_g2 | 23 | 1 | 4.504 | up | hypothetical protein LSAT_4X34220 (*Lactuca sativa*) |
| **334** | TRINITY_DN13434_c1_g2 | 31 | 1 | 5.564 | up | uncharacterized protein LOC113271997 (*Papaver somniferum*) |
| **335** | TRINITY_DN14263_c1_g2 | 17 | 0 | 5.533 | up | hypothetical protein CTI12_AA233810 (*Artemisia annua*) |
| **336** | TRINITY_DN13292_c3_g2 | 40 | 3 | 3.701 | up | Tubulin alpha-4 chain (*Zea mays*) |
| **337** | TRINITY_DN14261_c0_g1 | 89 | 8 | 3.483 | up | AP2-like ethylene-responsive transcription factor ANT (*Helianthus annuus*) |
| **338** | TRINITY_DN801_c0_g1 | 123 | 9 | 3.663 | up | delta(12)-fatty-acid desaturase-like (*Cynara cardunculus* var. *scolymus*) |
| **339** | TRINITY_DN10501_c0_g5 | 180 | 1 | 8.057 | up | cytochrome P450 77A3 (*Helianthus annuus*) |
| **340** | TRINITY_DN5003_c0_g1 | 29 | 1 | 4.789 | up | lamin-like protein (*Helianthus annuus*) |
| **341** | TRINITY_DN18674_c1_g1 | 265 | 1 | 8.050 | up | floral homeotic protein AGAMOUS (*Lactuca sativa*) |
| **342** | TRINITY_DN6065_c0_g1 | 75 | 1 | 6.127 | up | pectinesterase inhibitor domain-containing protein (*Artemisia annua*) |
| **343** | TRINITY_DN9815_c0_g1 | 62 | 1 | 5.437 | up | homeobox protein knotted-1-like 6 (*Lactuca sativa*) |
| **344** | TRINITY_DN13606_c1_g3 | 42 | 2 | 4.623 | up | alpha-L-fucosidase 3 (*Helianthus annuus*) |
| **345** | TRINITY_DN12385_c0_g2 | 120 | 2 | 6.146 | up | salutaridine reductase-like (*Lactuca sativa*) |
| **346** | TRINITY_DN5979_c0_g2 | 66 | 2 | 4.782 | up | isoprenoid synthase domain, Polyprenyl synthetase-related protein (*Artemisia annua*) |
| **347** | TRINITY_DN15278_c0_g2 | 26 | 2 | 3.605 | up | cytosolic purine 5'-nucleotidase (*Brassica rapa*) |
| **348** | TRINITY_DN17425_c0_g1 | 20 | 0.7 | 4.858 | up | hypothetical protein CTI12_AA453600 (*Artemisia annua*) |
| **349** | TRINITY_DN2255_c0_g1 | 54 | 2 | 4.741 | up | hypothetical protein HanXRQr2_Chr02g0047661 (*Helianthus annuus*) |
| **350** | TRINITY_DN15927_c2_g3 | 14 | 0.7 | 4.294 | up | putative UDP-glucuronosyl/UDP-glucosyltransferase (*Helianthus annuus*) |
| **351** | TRINITY_DN6052_c0_g1 | 21 | 1 | 3.893 | up | zinc finger, NHR/GATA-type (*Artemisia annua*) |
| **352** | TRINITY_DN6198_c0_g1 | 14 | 1 | 3.743 | up | PC-Esterase (*Artemisia annua*) |
| **353** | TRINITY_DN5458_c0_g1 | 60 | 5 | 3.444 | up | hypothetical protein LSAT_6X2881 (*Lactuca sativa*) |
| **354** | TRINITY_DN7794_c0_g2 | 171 | 15 | 3.434 | up | glycine-rich protein 5-like (*Helianthus annuus*) |
| **355** | TRINITY_DN12721_c2_g2 | 78 | 3 | 4.824 | up | growth-regulating factor 3 (*Helianthus annuus*) |
| **356** | TRINITY_DN7792_c0_g1 | 338 | 16 | 4.345 | up | aldehyde oxidase GLOX1 (*Helianthus annuus*) |
| **357** | TRINITY_DN9321_c1_g1 | 1235 | 96 | 3.627 | up | hypothetical protein E3N88_13027 (*Mikania micrantha*) |
| **358** | TRINITY_DN6277_c0_g1 | 44 | 2 | 4.723 | up | thionin-like protein 2 (*Helianthus annuus*) |
| **359** | TRINITY_DN15216_c0_g1 | 116 | 11 | 3.368 | up | Zinc finger, RanBP2-type (*Artemisia annua*) |
| **360** | TRINITY_DN17461_c1_g7 | 91 | 6 | 3.880 | up | leucine-rich repeat protein 1 (*Helianthus annuus*) |
| **361** | TRINITY_DN10627_c1_g4 | 1224 | 65 | 4.228 | up | non-specific lipid transfer protein GPI-anchored 1 (*Helianthus annuus*) |
| **362** | TRINITY_DN11753_c1_g1 | 74 | 6 | 3.599 | up | UDP-glucuronic acid decarboxylase 2-like (*Pyrus ussuriensis* × *Pyrus communis*) |
| **363** | TRINITY_DN8770_c0_g1 | 59 | 5 | 3.455 | up | protein SMAX1-LIKE 4-like (*Lactuca sativa*) |
| **364** | TRINITY_DN13221_c4_g1 | 67 | 5 | 3.615 | up | rho GTPase-activating protein 2 isoform X1 (*Helianthus annuus*) |
| **365** | TRINITY_DN8207_c0_g1 | 62 | 6 | 3.431 | up | uncharacterized protein LOC111883581 isoform X1 (*Lactuca sativa*) |
| **366** | TRINITY_DN18823_c2_g1 | 206 | 5 | 5.348 | up | non-classical arabinogalactan protein 31-like (*Cynara cardunculus* var. *scolymus*) |
| **367** | TRINITY_DN6606_c0_g1 | 50 | 4 | 3.487 | up | hypothetical protein LSAT_7X13860 (*Lactuca sativa*) |
| **368** | TRINITY_DN7923_c0_g1 | 102 | 5 | 4.415 | up | unnamed protein product (*Lactuca saligna*) |
| **369** | TRINITY_DN14992_c0_g1 | 435 | 5 | 6.331 | up | cytochrome P450 CYP736A12-like (*Cynara cardunculus* var. *scolymus*) |
| **370** | TRINITY_DN14389_c1_g2 | 3787 | 19 | 7.605 | up | RecName: Full=Germacrene A synthase short form; Short=CiGASsh (*Cichorium intybus*) |
| **371** | TRINITY_DN9686_c0_g1 | 151 | 14 | 3.378 | up | hypothetical protein AQUCO_00900205v1, partial (*Aquilegia coerulea*) |
| **372** | TRINITY_DN13940_c2_g1 | 337 | 24 | 3.798 | up | hypothetical protein E3N88_06037 (*Mikania micrantha*) |
| **373** | TRINITY_DN11099_c0_g1 | 2684 | 76 | 5.105 | up | GDSL esterase/lipase At5g45950 (*Helianthus annuus*) |
| **374** | TRINITY_DN4522_c0_g1 | 14 | 0 | 6.294 | up | hypothetical protein CTI12_AA022750 (*Artemisia annua*) |
| **375** | TRINITY_DN9130_c0_g1 | 69 | 6 | 3.574 | up | hypothetical protein CTI12_AA005090 (*Artemisia annua*) |
| **376** | TRINITY_DN14932_c2_g1 | 801 | 21 | 5.232 | up | lysine histidine transporter-like 8 (*Lactuca sativa*) |
| **377** | TRINITY_DN8830_c7_g2 | 166 | 6 | 4.691 | up | patatin-like protein 2 (*Cynara cardunculus* var. *scolymus*) |
| **378** | TRINITY_DN8524_c0_g1 | 1042 | 78 | 3.718 | up | putative cytochrome P450 (*Helianthus annuus*) |
| **379** | TRINITY_DN17091_c2_g7 | 283 | 19 | 3.846 | up | 1-deoxy-D-xylulose-5-phosphate synthase precursor (*Eschenbachia blinii*) |
| **380** | TRINITY_DN8926_c2_g3 | 682 | 42 | 3.975 | up | unnamed protein product (*Lactuca saligna*) |
| **381** | TRINITY_DN11255_c0_g1 | 79 | 7 | 3.445 | up | xyloglucan endotransglucosylase/hydrolase protein 31-like (*Cynara cardunculus* var. *scolymus*) |
| **382** | TRINITY_DN19720_c1_g1 | 2692 | 169 | 3.939 | up | cellulose synthase-like protein G2 (*Lactuca sativa*) |
| **383** | TRINITY_DN14533_c5_g1 | 2053 | 0 | 13.458 | up | geranylgeranyl diphosphate reductase, chloroplastic-like (*Cynara cardunculus* var. *scolymus*) |
| **384** | TRINITY_DN9829_c4_g1 | 98 | 0 | 9.044 | up | inactive protein RESTRICTED TEV MOVEMENT 1 (*Helianthus annuus*) |
| **385** | TRINITY_DN12045_c1_g1 | 87 | 8 | 3.465 | up | Alpha/beta hydrolase fold-1 (*Artemisia annua*) |
| **386** | TRINITY_DN20125_c0_g1 | 33 | 0.1 | 8.688 | up | agamous-like MADS-box protein AGL11 (*Lactuca sativa*) |

JePB-W: colourless region of JePB, JePB-C: coloured region of JePB. The expression levels of the above listed genes were at least ten times different between colourless and coloued regions (|Log_2_FoldChange|≥Log_2_10). FoldChange: The radio of gene FPKM values in JePB-W compared to JePB-C. All data are presented as the mean from three biological replicates.

**Table S6. MIKC type MADS-box TFs are shown in the gene tree.**

| Amino acid sequence | Genomic Locus | Genbank number |
| --- | --- | --- |
| AtAP3 | [At3g54340](http://arabidopsis.org/servlets/TairObject?type=locus&name=At3g54340) | [M86357](http://www.ncbi.nlm.nih.gov/entrez/query.fcgi?cmd=Search&db=Nucleotide&term=M86357&doptcmdl=GenBank) |
| AtPISTILLATA | [At5g20240](http://arabidopsis.org/servlets/TairObject?type=locus&name=At5g20240) | [D30807](http://www.ncbi.nlm.nih.gov/entrez/query.fcgi?cmd=Search&db=Nucleotide&term=D30807&doptcmdl=GenBank) |
| AtAGAMOUS | [At4g18960](http://arabidopsis.org/servlets/TairObject?type=locus&name=At4g18960) | [X53579](http://www.ncbi.nlm.nih.gov/entrez/query.fcgi?cmd=Search&db=Nucleotide&term=X53579&doptcmdl=GenBank) |
| AtSHP1 | [At3g58780](http://arabidopsis.org/servlets/TairObject?type=locus&name=At3g58780) | [M55550](http://www.ncbi.nlm.nih.gov/entrez/query.fcgi?cmd=Search&db=Nucleotide&term=M55550&doptcmdl=GenBank) |
| AtSEP1 | [At5g15800](http://arabidopsis.org/servlets/TairObject?type=locus&name=At5g15800) | [M55551](http://www.ncbi.nlm.nih.gov/entrez/query.fcgi?cmd=Search&db=Nucleotide&term=M55551&doptcmdl=GenBank) |
| AtAGL3 | [At2g03710](http://arabidopsis.org/servlets/TairObject?type=locus&name=At2g03710) | U81369 |
| AtSEP2 | [At3g02310](http://arabidopsis.org/servlets/TairObject?type=locus&name=At3g02310) | M55552 |
| AtSHP2 | [At2g42830](http://arabidopsis.org/servlets/TairObject?type=locus&name=At2g42830) | M55553 |
| AtAGL6 | [At2g45650](http://arabidopsis.org/servlets/TairObject?type=locus&name=At2g45650) | M55554 |
| AtAP1 | [At1g69120](http://arabidopsis.org/servlets/TairObject?type=locus&name=At1g69120) | [Z16421](http://www.ncbi.nlm.nih.gov/entrez/query.fcgi?cmd=Search&db=Nucleotide&term=Z16421&doptcmdl=GenBank) |
| AtFUL | [At5g60910](http://arabidopsis.org/servlets/TairObject?type=locus&name=At5g60910) | [U33473](http://www.ncbi.nlm.nih.gov/entrez/query.fcgi?cmd=Search&db=Nucleotide&term=U33473&doptcmdl=GenBank) |
| AtSEP3 | [At1g24260](http://arabidopsis.org/servlets/TairObject?type=locus&name=At1g24260) | [AF015552](http://www.ncbi.nlm.nih.gov/entrez/query.fcgi?cmd=Search&db=Nucleotide&term=AF015552&doptcmdl=GenBank) |
| AtCAL | [At1g26310](http://arabidopsis.org/servlets/TairObject?type=locus&name=At1g26310) | [L36925](http://www.ncbi.nlm.nih.gov/entrez/query.fcgi?cmd=Search&db=Nucleotide&term=L36925&doptcmdl=GenBank) |
| AtAGL11 | [At4g09960](http://arabidopsis.org/servlets/TairObject?type=locus&name=At4g09960) | [U20182](http://www.ncbi.nlm.nih.gov/entrez/query.fcgi?cmd=Search&db=Nucleotide&term=U20182&doptcmdl=GenBank) |
| AtAGL12 | [At1g71692](http://arabidopsis.org/servlets/TairObject?type=locus&name=At1g71692) | [U20193](http://www.ncbi.nlm.nih.gov/entrez/query.fcgi?cmd=Search&db=Nucleotide&term=U20193&doptcmdl=GenBank) |
| AtAGL13 | [At3g61120](http://arabidopsis.org/servlets/TairObject?type=locus&name=At3g61120) | [U20183](http://www.ncbi.nlm.nih.gov/entrez/query.fcgi?cmd=Search&db=Nucleotide&term=U20183&doptcmdl=GenBank) |
| AtAGL14 | [At4g11880](http://arabidopsis.org/servlets/TairObject?type=locus&name=At4g11880) | [U20184](http://www.ncbi.nlm.nih.gov/entrez/query.fcgi?cmd=Search&db=Nucleotide&term=U20184&doptcmdl=GenBank) |
| AtAGL15 | [At5g13790](http://arabidopsis.org/servlets/TairObject?type=locus&name=At5g13790) | [U20185](http://www.ncbi.nlm.nih.gov/entrez/query.fcgi?cmd=Search&db=Nucleotide&term=U20185&doptcmdl=GenBank) |
| AtAGL16 | [At3g57230](http://arabidopsis.org/servlets/TairObject?type=locus&name=At3g57230) | NM_115583 |
| AtAGL17 | [At2g22630](http://arabidopsis.org/servlets/TairObject?type=locus&name=At2g22630) | NM_127828 |
| AtAGL18 | [At3g57390](http://arabidopsis.org/servlets/TairObject?type=locus&name=At3g57390) | [AF312663](http://www.ncbi.nlm.nih.gov/entrez/query.fcgi?cmd=Search&db=Nucleotide&term=AF312663&doptcmdl=GenBank) |
| AtAGL19 | [At4g22950](http://arabidopsis.org/servlets/TairObject?type=locus&name=At4g22950) | [AF312664](http://www.ncbi.nlm.nih.gov/entrez/query.fcgi?cmd=Search&db=Nucleotide&term=AF312664&doptcmdl=GenBank) |
| AtSOC1 | [At2g45660](http://arabidopsis.org/servlets/TairObject?type=locus&name=At2g45660) | [AY007726](http://www.ncbi.nlm.nih.gov/entrez/query.fcgi?cmd=Search&db=Nucleotide&term=AY007726&doptcmdl=GenBank) |
| AtAGL21 | [At4g37940](http://arabidopsis.org/servlets/TairObject?type=locus&name=At4g37940) | [AF336979](http://www.ncbi.nlm.nih.gov/entrez/query.fcgi?cmd=Search&db=Nucleotide&term=AF336979&doptcmdl=GenBank) |
| AtSVP | [At2g22540](http://arabidopsis.org/servlets/TairObject?type=locus&name=At2g22540) | [AF211171](http://www.ncbi.nlm.nih.gov/entrez/query.fcgi?cmd=Search&db=Nucleotide&term=AF211171&doptcmdl=GenBank) |
| AtAGL24 | [At4g24540](http://arabidopsis.org/servlets/TairObject?type=locus&name=At4g24540) | [AF005158](http://www.ncbi.nlm.nih.gov/entrez/query.fcgi?cmd=Search&db=Nucleotide&term=AF005158&doptcmdl=GenBank) |
| AtFLC | [At5g10140](http://arabidopsis.org/servlets/TairObject?type=locus&name=At5g10140) | [AF537203](http://www.ncbi.nlm.nih.gov/entrez/query.fcgi?cmd=Search&db=Nucleotide&term=AF537203&doptcmdl=GenBank) |
| AtFLM | [At1g77080](http://arabidopsis.org/servlets/TairObject?type=locus&name=At1g77080) | [AF342808](http://www.ncbi.nlm.nih.gov/entrez/query.fcgi?cmd=Search&db=Nucleotide&term=AF342808&doptcmdl=GenBank) |
| AtAGL31 | [At5g65050](http://arabidopsis.org/servlets/TairObject?type=locus&name=At5g65050) | [AF312667](http://www.ncbi.nlm.nih.gov/entrez/query.fcgi?cmd=Search&db=Nucleotide&term=AF312667&doptcmdl=GenBank) |
| AtTT16 | [At5g23260](http://arabidopsis.org/servlets/TairObject?type=locus&name=At5g23260) | [AJ318098](http://www.ncbi.nlm.nih.gov/entrez/query.fcgi?cmd=Search&db=Nucleotide&term=AJ318098&doptcmdl=GenBank) |
| AtAGL42 | [At5g62165](http://arabidopsis.org/servlets/TairObject?type=locus&name=At5g62165) | [AY141213](http://www.ncbi.nlm.nih.gov/entrez/query.fcgi?cmd=Search&db=Nucleotide&term=AY141213&doptcmdl=GenBank) |
| AtANR1 | [At2g14210](http://arabidopsis.org/servlets/TairObject?type=locus&name=At2g14210) | [Z97057](http://www.ncbi.nlm.nih.gov/entrez/query.fcgi?cmd=Search&db=Nucleotide&term=Z97057&doptcmdl=GenBank) |
| AtAGL63 | [At1g31140](http://arabidopsis.org/servlets/TairObject?type=locus&name=At1g31140) | [AY141243](http://www.ncbi.nlm.nih.gov/entrez/query.fcgi?cmd=Search&db=Nucleotide&term=AY141243&doptcmdl=GenBank) |
| AtAGL68 | [At5g65080](http://arabidopsis.org/servlets/TairObject?type=locus&name=At5g65080) | [AY231455](http://www.ncbi.nlm.nih.gov/entrez/query.fcgi?cmd=Search&db=Nucleotide&term=AY231455&doptcmdl=GenBank) |
| AtAGL69 | [At5g65070](http://arabidopsis.org/servlets/TairObject?type=locus&name=At5g65070) | [AY231450](http://www.ncbi.nlm.nih.gov/entrez/query.fcgi?cmd=Search&db=Nucleotide&term=AY231450&doptcmdl=GenBank) |
| AtAGL70 | [At5g65060](http://arabidopsis.org/servlets/TairObject?type=locus&name=At5g65060) | [AY231445](http://www.ncbi.nlm.nih.gov/entrez/query.fcgi?cmd=Search&db=Nucleotide&term=AY231445&doptcmdl=GenBank) |
| AtAGL71 | [At5g51870](http://arabidopsis.org/servlets/TairObject?type=locus&name=At5g51870) | [AY141220](http://www.ncbi.nlm.nih.gov/entrez/query.fcgi?cmd=Search&db=Nucleotide&term=AY141220&doptcmdl=GenBank) |
| AtAGL72 | [At5g51860](http://arabidopsis.org/servlets/TairObject?type=locus&name=At5g51860) | [AY141221](http://www.ncbi.nlm.nih.gov/entrez/query.fcgi?cmd=Search&db=Nucleotide&term=AY141221&doptcmdl=GenBank) |
| AtAGL79 | [At3g30260](http://arabidopsis.org/servlets/TairObject?type=locus&name=At3g30260) | [AY141238](http://www.ncbi.nlm.nih.gov/entrez/query.fcgi?cmd=Search&db=Nucleotide&term=AY141238&doptcmdl=GenBank) |
| LsAGAMOUS |  | XP_023732805.1 |
| CmCDM37 |  | AAO22984.1 |
| GhGAGA1 |  | CAA08800.1 |
| HaAGAMOUS isoform X1 |  | XP_022038382.1 |
| ClAG1 |  | AIC33049.1 |
| HaHAM45 |  | AAO18228.1 |
| TeAG2 |  | QOJ53907.1 |
| AaAG2 |  | PWA74414.1 |
| LsAGL11 |  | XP_023760420.1 |
| CeAGL11 |  | XP_027150164.1 |
| CaAGL11 |  | XP_027091158.1 |
| HaAGL11 |  | XP_021983447.1 |
| ItAGL11 |  | XP_031123124.1 |
| SiAGL11 |  | XP_011095997.1 |
| NtAGL11 |  | XP_009599024.1 |

**Table S7. Sequences of *ScCHS2*, *ScF3H1*, and *ScANS* promoters cloned from genomic DNA of JeCB ray florets.**

| **Promoter** | **Sequence** | **Length** |
| --- | --- | --- |
| *ScCHS2* | TCGGTACCCGGGGATCCTCTAGAGATTAGTGGAGTATCAGAGACAGTTACACCAATGCGAGACATGCTTATGTCTGTTGGTTTTTATTTGAAAATTTTTGATACATCTCCCGACAGACCTTCCAATTTTATGGTCACAATCTTCTCATCAACTAATTAAAATTTCAAAAAAATAAAAATAAAAATCACGGAGTCTCCTTTTCTAATTAAATCGTTGTAATTTTTTTGATTTTTTATTGTTTTTAAATAACATATAATGTACACGTTTATTAATACGAAAAAATATAAAAACAATAACATACAAACATTTTTAAAAGCTGAAAAAAAAATATATTTGCAAATATTGTATTATACATAACTTACCATTATAATTTGCCGATTATACTATCAATATTATATAAGCATTAACTGTTTTGAGTTTTTTTATTTATAAATACATATATAACAAATCATATATCAAAATTCTTATTTTTATTATTTATCGGACTTGAATCCATAACGACACAATTTAATGTTACTTGGTGAACTAACATACGATTAGTATGAAGTCATTTGACGTGTTTTAAGTTAACATGCCTCACGAATGAAATTGAATTCGAAATTGGTCTCGTCTGTGTGTTTCGCCAGATATACTGTTTATATCGTACAAGACAATATGAGACTTACAAAAAGCAATAACGATCTTCTTGTTTGTGAGTGTTAAAATGAATTTAGGTTGTTTGATGGGCAAGTGAGGGTCACGTGCCATCTATCAAGTTGTGACTGGGGAGTTATGCATGTGATACCCATCCACCATTCTACGTACCAAACGTTTACGTTTATTATAAATTGTAACCACACAAAGACTACTCAACAAACAAATTCAATTACAAGTTATTAATTCCCATCAACATACTCTAACTTCATCCCGCA **ATG** | 911bp |
| *ScF3H1* | ACGCGTGGTCGACGGCCCGGGTTGGTACGAACTTGAAAAGTCAAACGAAGTTGAAAGTCAAACGAATGTGAAAAGTCAACGAACTTGAAAATTCAAACGAACTTGAAAAGTCAACGAACTTGAAAAGTTAAATGAACATGAAAAATCAAACGAATTTGAAAAAGTCAAACGAACTTAAAAAAAGTCAAACGAACTTGAAAAATCATACGAACTTCGACTTTGACCAGCGAACTTTGATTTTGACTAGTGAACCTTTGACCAGCTAACTTGAACTTTGACCAGCGAACTTTGACTTTGACTACCGAACTTTAACTTTGAATTAGAGTTTGCTAGGTTTTCTGTGCCTCTCTAACGTTCACCATTAGATGAAAAAATTGAATTTTTGTAAAAAAGTGCACAAATAAATGACCGGAAAAATTCCCAAAAATGACCGGAATAATTTAAAAAAAACGAAAAACATAAGATAAAGAACATGAAGATGAACGTGTGAATGGTATCCCAAAATTGAAACTAAAAAATGGTGAAAAAATCTTCTGAAAAACAGAAGAAGAAACCCGAAAATGTGTAGAAATTCGTAAAAAAAGAATGAAACGGTAGAAACAGAGCAGCTATGGATCAGAAAAAGCCGCTCTGATACAATGAGAAATTTATGAGCTCGAATAAAATGGAAAGAGAATTAAAGGAAGAAAATTATTTCATTAACTTTCAAATGAAACTATTACAAACTATTTATAAAAAATTAAATTACATAAATAGAACAAACTATCTAATAAATACGTAAGCTACCCTACACTTTATATAAATATATATATCTCTATAAATAGTAAGTGTGACACTTATTTTAGAATAATTATGTTTTTTATTAATTAATATAAATGATTACGTAAAAACAACAAATAGGTTTTTGTTGGATGAAGGATGGTCTAACTACCATTCAATAAGTTATATGTTGGACGAGGATGGTCTAACTACCATTCAATAAGTTATATGTTGGACTTCATTACTACGTACGTGTGGTAGTTGAAGTTGCATTCTAACCCCATATGCAACCTACTATTCAATTCTCTCCCCTACATATATACTTCAAAGGGAATTCCATTGATCCTACTACTAAACACACACATAACACATGAAA **ATG** | 1135bp |
| *ScDFR3* | AGGAGAAGCATAGGTAACAAAAAGTAAACACATGTTGAAACAATAGTTTCCCTCACTCACCAACATGTGCTACCCAACGGTCAAACTTCCACTCCCACCGGTCTCCTTTTATAAGCTAATAACTCAAATCCAACATCCTGGCTTGTGGGTGCGGATATCTCTACCCGTCATTCATATCTGTATATTTTTCTTTACCCATCAATCTGCGTGTATTTATTGATGGATTTTTTTTCCTACTCATACCCGGGAGCCGCAGGTACACGTGATTTGCGGATAACCAACAGGTTATATAAATTTATATCAAATTTTTGAACTTGTCAACGATTTAGTGAAAAATGCTAACAAATTACAAAAAAAAAATAAAAATAAATTAAATTATAAGTAAATAGGTTTAAAGTAAATGCGTAACATACTTTTCCAAAAAACATAAAAGATATTAATTTTTATTATTGCCGAGAATAAAAATTTAAACCTTAACAAACATTCATATTTTTTGGGTATATAATACATAGGTACCTCAAATGAGTATTATTGGTTAACGGGACGAATATACCCGTGACGGATATAACATATCTATTATTCGCTAACGGGTACAATTATTTTACCAATAATCGTGTCCGCAGATGAAAATTCACAAATTTATTTACCCATCACCGGTAGATATCCGCGAGGTGCGGTTTTTTTTTCTATTCCCATCCCTACTCTAATCTTAATCTACGTGGAGTTGAATGGTGGGTTTGGCACGTTCTTTTTTTAACACTAAATCCTTCTATAAAAAAAACACATCCTAATTCTTATTTTACTATCAAAATAATCCAGAGCTCTCATAAACCAG **ATG** | 835bp |

**Table S8. *Cis*-elements identified in the *ScCHS2* promoter.**

| **Cis-element** | **Core sequence(5’-3’)** | **Location** |
| --- | --- | --- |
| ABRE | CACGTG/ACGTG | 260(-)/554(+)/737(-)/738(+) |
| AT-TATA-box | TATATA | 393/437(+) |
| BOX 4 | ATTAAT | 267(+)/874(-) |
| CAAT-box | CAAT/CAAAT | 51/52(+)/85(-)/121/122/136(+)/234(-)/290(+）/331(-）/336(+）/341/369(-）/387/444/503(+）/549/587/599(-）/650/671(+）/826(-）/856/862/909(+) |
| CGTCA-motif | CGTCA | 552(-) |
| ARE | AAACCA | 77(-) |
| G-box | CACGAC | 260(+)//553/737(-) |
| MRE | AACCTAA | 709(-) |
| MYB | CAACAG | 43(-)/404(+)73(-)/830(+) |
| Myb-like sequence | TAACCA | 830(+) |
| E-box | CATTTG/CATGTG | 548/774(+) |
| TATA-Box | ATATAT/TATA/TATAAATA | 240(-)/250/251/281/282/327/328/346(+)/347(-)/348/363(+)/364(-)/365/377(+)/378(-)/379/391(+)/392(-)/393/394/395(+)/423/424/425/426(-)/427/436/437/438/439/450/451(+)/627/634/635/636(-)/662/819(+)/820/821(-) |
| TGA-element | AACGAC | 496(+) |
| TGACG-motif | TGACG | 552(+) |
| GARE-motif | TCTGTTG | 72(+) |
| as-1 | TGACG | 552(+) |
| CArG | C(A/T)_8_G | 399/790(+) |

**Table S9. *Cis*-elements identified in the *ScF3H1* promoter.**

| **Cis-element** | **Core sequence(5’-3’)** | **Location** |
| --- | --- | --- |
| ABRE | AACCCGG/GACACGTACGT/ACGTG | 16/1005(-)、1009/482(+) |
| ABRE3a | TACGTG | 1008(+) |
| ABRE4 | CACGTA | 1008(-) |
| ACE | CTAACGTATT | 772(-) |
| AT-TATA-box | TATATA | 793/795/1075/803/805(-) |
| BOX 4 | ATTAAT | 861/865(-) |
| CAAT-box | CAAT/CAAAT | 893/636/1058/706/935/397/976(+)/153/1097/373/503(-) |
| G-box | CACGAC | 481(-) |
| LTR | CCGAAA | 554(+) |
| E-box | CATTTG | 706/1126(-) |
| STRE | AGGGG | 1067(-) |
| TATA-Box | ATATAT/TATA/TATAAATA | 745/869/813/796(+)/380/806/793/943/729/815/802/1075/727/807/795/984/870/1077/805/803/726/983/794/730/1074/797/728/942/792/804(-) |
| TATC-box | TATCCCA | 494(+) |
| TCA | TCATCTTCAT | 131/472(-) |
| W box | TTGACC | 219/274/254(+) |
| CArG | C(A/T)_8_G | 291/616/729(+) |

**Table S10. *Cis*-elements identified in the *ScDFR3* promoter.**

| **Cis-element** | **Core sequence(5’-3’)** | **Location** |
| --- | --- | --- |
| AAGAA-motif | gGTAAAGAAA | 186/638(-) |
| ABRE | CACGTG/ACGTG | 146/148(-) |
| ABRE3a | TACGTG | 715(-) |
| ABRE4 | CACGTA | 715(-) |
| ARE | AAACCA | 828（+） |
| AT~TATA-box | TATATA | 286(+)/498(-) |
| Box 4 | ATTAAT | 435(-) |
| CAAT-box | CAAT/CAAAT/CCAAT | 40/124/199/301/342/518/593/604/605/634(+)/215/265/448/530(-) |
| CGTCA-motif | CGTCA | 165(+)/556(-) |
| CCAAT-box | CAACGG | 74(+) |
| G-box | CACGTG/TACGTG/CACGTT | 259/715/741(+) |
| MYB | CAACAG | 274/278(+)/532(-) |
| MYB recognition site | CCGTTG | 74(-) |
| MSA-like | (T/C)C(T/C)AACGG(T/C)(T/C)A | 72(+) |
| Myb-binding site | CAACAG | 278(+) |
| MYB-like sequence | TAACCA | 274(+)/532(-) |
| MYC | CATGTG | 63(+)/28/518(-) |
| TGACG-motif | TGACG | 165(-)/556(+) |
| TATA-box | TATAAAA/TATAAA/TATAA/TATA/TATACA/ATATAA/TATAAAT/TACAAAA/ccTATAAAa | 106/107/108/178/285/293/294/295/375/498/500/548/563/770(-)/109/180/286/287/288/296/347/374/376/499/562/768(+) |
| TC-rich repeats | ATTCTCTAAC | 578(+) |
| GT1-motif | GGTTAA | 533(+) |
| as-1 | TGACG | 165(-)/556(+) |
| W box | TTGACC | 78(-) |
| CArG | C(A/T)_8_G | 525/720(+) |

**Table S11. The primers used in this study**

| Primers | Sequences (5’-3’) | Reverses（5’-3’） |
| --- | --- | --- |
| *ScCHS2* | CCAGATAGCGAGGGAGCAA | CCACGAATCCAACATCAAAT |
| *ScCHS3* | CCGATTGGAACTCGCTCTT | ATACGCATGTTGGTTGGGA |
| *ScCHI2* | AATGAGAAAGCAGCAGATGC | CGATTAGGTGAATAGGTTGTAGG |
| *ScF3H1* | TGACTGGTGGGAAGAAGG | TTAGGTCAGGATGAGGACACTT |
| *ScF3H5* | CTTTCTGGATTGATAAACGAGG | CCGATGTCATTACCACCGA |
| *ScF3H6* | CTGGTAACGGCTACGGAAAG | TTGTGGGAGGAGTGGGAAT |
| *ScF3H7* | TTCAGTTGCCGACAGAGGA | TGGACAGCCGACTTGTATTT |
| *ScF3’H1* | GTTGGAAACCTGCCACACCT | TCTCTTGCCTAACATCACTCG |
| *ScF3’H2* | ATTTAACTAGTGATGGGAGCCG | TAAACGAGGACTAGCAACAGCC |
| *ScDFR3* | ATCACACCCTCGTTCCCACCAA | TTCGGGTTCTCGTAGAGGGATA |
| *ScDFR5* | ACCTCTTGAACTCGCCTACG | CACCTGGTTGACCTTTGACTA |
| *ScANS* | GACATTACCTCCGATGACCC | TTCCCACCGACCTCTTTCT |
| *ScGST3* | TACTGAGTCACCGAGCCGTTGG | TACCTTCACAAACTCGCACCGC |
| *ScMYB7* | TTCATAGCCACTGAACTTCCA | ATTTCGCTTTCGTCGTCTT |
| *ScMYB8* | AAGGTTGTTGGCGTTCCC | TGATGATACGGCTTCGTTTTC |
| *ScMYB10* | TTGATTGCGGGAAGAATACC | GCCTCCTCGTTTGCTACAGA |
| *ScMYB22* | GGTTTACACCGTGGTTCATG | GGCGTTCCAGTGGTTCTT |
| *ScMYB23* | GGTAACAGGTGGTCTTTGATAG | GGTCACTTTCATTTTTCTTGGC |
| *ScMYB28* | AAAAGGAACAGTTGCATCGA | CACTAAACCGTCATTACCACCC |
| *ScMYB31* | TAGAACAGTGGTTGGCAGAA | AAAAAAGCGAAGTCGGGAAT |
| *ScMYB48* | TTGCGAACCATCCAAACAC | AATCCCTCCACATCCATCAC |
| *ScbHLH1* | AATCTTTTCCCTACAACGG | CCTTCTTCAACCATCCTTT |
| *ScbHLH6* | AAAACTTAGTCAGTGGTCCGTG | GCTTTTCTTTTGTTGGCGTA |
| *ScbHLH17* | CACCACCTTCCTACCAAAACC | CCCCTTCCTTCTGTTATTC |
| *ScbHLH21* | CAACTGAGCATTTGGAGGG | CGAGCAGTCAATGATAAGGAG |
| *ScAG* | TTGTAAGCGGCGTAATGGG | CGAAGGGTTGGTGTTGTGTC |
| *ScAGL11* | CTCTACTAGGGGTCGGGTCT | AGTAATCTTTGAGCGAAGGAAC |
| *Scβ-actin* | TCCACATGCCATTCTTCGTCT | CAAAGCGGTAATTTCCTTGCT |
| *NtCHS* | TTGTTCGAGCTTGTCTCTGC | AGCCCAGGAACATCTTTGAG |
| *NtCHI* | GTCAGGCCATTGAAAAGCTC | CTAATCGTCAATGCCCCAAC |
| *NtF3H* | CAAGGCATGTGTGGATATGG | TGTGTCGTTTCAGTCCAAGG |
| *NtF3‘H* | AGGCTCAACACTTCTCGT | CATCAACTTTGGGCTTCT |
| *NtDFR* | AACCAACAGTCAGGGGAATG | TTGGACATCGACAGTTCCAG |
| *NtANS* | TGGCGTTGAAGCTCATACTG | GGAATTAGGCACACACTTTGC |
| *NtAN1a* | ACCATTCTCGAACACCGAAG | TGCTAGGGCACAATGTGAAG |
| *NtAN1b* | CTTGAACACTTCTCAAACCGA | TGCTAGGGCACAATGTGAAG |
| *NtACT* | AATGGAACTGGAATGGTCAAGGC | TGCCAGATCTTCTCCATGTCATCCCA |
| *ScCHS2*-qRT | TGCGACACTCTTCAGCTACAGAC | ACACTATGTAGGACCACGGTCTC |
| *ScF3H1*-qRT | TGGCTCGTCTCAAGAAACTAGC | TAATGGCATTTTCTGACCTGAG |
| *ScDFR3-*qRT | ATCACACCCTCGTTCCCACCAA | TTCGGGTTCTCGTAGAGGGATA |
| *ScANS*-qRT | TTGGCGACACGATTGAGATTCTAAG | TGTCACCGTTCTTCCTAAACAACTT |
| *ScbHLH17*-qRT | TCTCCTAAACAAACTCATTCGGCTC | TCTTCGTCCATAGAATACATGGTGC |
| *ScAG*-qRT | GAGAACACAACGAATCGCCAA | TAAGAGCAACCTCAGCATCACAA |
| *ScAGL11*-qRT | TCTTATGGGTGACGGGTTG | TCTATCGCATTGTATTGCTGAC |
| *Scβ-actin*-qRT | TCCACATGCCATTCTTCGTCT | CAAAGCGGTAATTTCCTTGCT |
| *ScAG*-CDS | ATGGAAAATTCTGATGCCATTG | TTACACCAACTGGAGAGGGG |
| *ScAGL11*-CDS | ATGGGAAGAGGAAAGATCGAG | TCACCCAAGGTGAAGAGACTTG |
| *ScbHLH17*-CDS | ATGGTTGGAGTTGATCCACATTATG | TTAGTGCAAGGGAGATATTATTTG |
| *ScAG*-pBI121/AD/BD | CCGGAATTCATGGAAAATTCTGATGCC | CGCGGATCCTTACACCAACTGGAGAGG |
| *ScAGL11* -pBI121/AD/BD/SK | CCGGAATTCATGGGAAGAGGAAAGATC | CGCGGATCCTCACCCAAGGTGAAGAGA |
| AD-vector | AGATGGTGCACGATGCACAG | |
| BD-vector | TTTTCGTTTTAAAACCTAAGAGTC | |
| 35S | GACGCACAATCCCACTATCC | |
| NOS | GCCAAATGTTTGAACGATCGG | |
| *ScAG*-*TRV* | CGACGACAAGACCCTAGATAGCTGAAAATGAAAGAGCTCA | GAGGAGAAGAGCCCTTTACACCAACTGGAGAGGGGTTTGG |
| *ScAGL11*-*TRV* | CGACGACAAGACCCTCAAAGATTACTGAAAATGAGAGGGT | GAGGAGAAGAGCCCTTCACCCAAGGTGAAGAGACTTGTTT |
| *TRV1* | GATGTGGCTGCCGTATTG | ATCGTCCTCTTTCACAGTTTCA |
| *TRV2* | CTGTTTGAGGGAAAAGTAG | CAAAAGACTTACCGATCAATC |
| *ScAG*-YCE/YNE | GAGAACACGGGGGACTCTAGAATGGAAAATTCTGATGCCATTG | GACAGTACTATCGATGGATCCCACCAACTGGAGAGGGG |
| *ScAG*-GFP | CATTTACGAACGATACTCGAGATGGAAAATTCTGATGCCATTG | TCACCATCACTAGTACGTCGACCACCAACTGGAGAGGGG |
| *ScAGL11-*GFP | CATTTACGAACGATACTCGAGATGGGAAGAGGAAAGATCGAG | TCACCATCACTAGTACGTCGACCCCAAGGTGAAGAGACTTG |
| *ScAGL11-*YCE/YNE | GAGAACACGGGGGACTCTAGAATGGGAAGAGGAAAGATCGAG | GACAGTACTATCGATGGATCCCCCAAGGTGAAGAGACTTG |
| *ScCHS2*-Pro-SP1/2/3 | TCATTCGTGAGGCATGTTAA/ATGGATTCAAGTCCGATAAA/TTGATAGTATAATCGGCAAA | |
| *ScF3H1*-Pro-SP1/2/3 | CGCCTTTCTTCCCACCAGT/CAAATATCTGCCCTACGACC/GCTGCTCTGTTTCTACCGTTTC | |
| *ScDFR*3-Pro-SP1/2/3 | GATTAGAGTAGGGATGGGAATAGAAA/ATTGTACCCGTTAGCGAATAATAGAT/TACCTATGCTTCTCCACTAATCTCTA | |
| *ScCHS2*-Pro | GACAGTTACACCAATGCGAGAC | CATTGCGGGATGAAGTTAGA |
| *ScF3H1*-Pro | GTCAAACGAATGTGAAAAGTC | ATATGGGGTTAGAATGCA |
| *ScDFR*3-Pro | AGGAGAAGCATAGGTAACAAAAAGTAAAC | CTGGTTTATGAGAGCTCTGGATTATTT |
| pAbAi-*ScCHS2*-pro-1/2 | AAGCTTGAATTCGAGCTTTCCAATTTTATGGTCACAATCTTC | GAGCACATGCCTCGAGGTCGAC+AAGTAACATTAAATTGTGTCGTTAT |
| pAbAi-*ScF3H1*-pro-1 | AAGCTTGAATTCGAGCTGCGAACTTTGACTTTGACTACCGAA | GAGCACATGCCTCGAGGTCGACAATTTTTCCGGTCATTTATTTGTGC |
| pAbAi-*ScF3H1*-pro-2 | AAGCTTGAATTCGAGCTTTGAAACTAAAAAATGGTGAAAAAA | GAGCACATGCCTCGAGGTCGACCTCATTGTATCAGAGCGGCTTTTTC |
| pAbAi-*ScF3H1*-pro-3 | AAGCTTGAATTCGAGCT+GTAAGTGTGACACTTATTTTAGAAT | GAGCACATGCCTCGAGGTCGACTCGTCCAACATATAACTTATTGAAT |
| pAbAi-*ScDFR3*-pro-1 | AAGCTTGAATTCGAGCTAGGAGAAGCATAGGTAACAAAAAG | GAGCACATGCCTCGAGGTCGACTATTAGCTTATAAAAGGAGACCGGT |
| pAbAi-*ScDFR3*-pro-2 | AAGCTTGAATTCGAGCTTCAAATTTTTGAACTTGTCAACGAT | GAGCACATGCCTCGAGGTCGACATGTTTTTTGGAAAAGTATGTTACG |
| pAbAi | GTTCCTTATATGTAGCTTTCGACA | CCATCTCGAAAAAGGGTTTGCC |
| *ScDFR*-Luc | CTATAGGGCGAATTGGGTACCAGGAGAAGCATAGGTAACAAAAAG | CAGGAATTCGATATCAAGCTTCTGGTTTATGAGAGCTCTGGAT |
| *ScF3H1*-Luc | CTATAGGGCGAATTGGGTACCGTCAAACGAATGTGAAAAGTC | CAGGAATTCGATATCAAGCTTATATGGGGTTAGAATGCA |
